# FNR-like unit interacts with C-terminal related residues trigger nNOS reductase domain conformational flexibility change

**DOI:** 10.3389/fnins.2026.1751011

**Published:** 2026-02-11

**Authors:** Yubo Wang, Nan Wang

**Affiliations:** 1 Research Center for Biochemistry and Molecular Biology, Jiangsu Key Laboratory of Brain Disease Bioinformation, Xuzhou Medical University, Xuzhou, Jiangsu, China; 2 National Demonstration Center of Experimental Basic Medical Science Education, Xuzhou Medical University, Xuzhou, Jiangsu, China

**Keywords:** FAD, FMN, FNR-like unit, molecular dynamic simulation, NADP(H), nNOS reductase domain

## Abstract

**Introduction:**

The reductase domain of neuronal nitric oxide synthase (nNOS) is essential for nitric oxide (NO) production in the mammalian nervous system. Excessive NO contributes to neurological disorders, including ischemic stroke, highlighting the need to better understand the structural dynamics of this domain. The FNR- like unit within the reductase domain stabilizes the cofactors NADP(H), FAD, and FMN, which are critical for NO synthesis. However, the dynamic interactions between these cofactors and key residues remain poorly characterized, limiting the ability of current nNOS inhibitors to normalize enzyme activity in cerebral ischemia–reperfusion injury.

**Methods:**

In this study, we evaluated the effects of widely used nNOS inhibitors, including spermidine (Spe) and L-NMMA, on nNOS activity using cellular NO assays and Western blot analysis. To investigate the structural basis of this resistance, we constructed three molecular models representing distinct redox states of NADP(H), FAD, and FMN, and derived new parameters for these cofactors (oxidized and reduced forms) using the def2-TZVP basis set.

**Results:**

While those inhibitors showed some therapeutic benefit in ischemia–reperfusion injury, they failed to suppress nNOS activity to physiological levels. RMSD analysis confirmed conformational stability after ~1.0 μs of simulation. Hydrogen bond and polar contact analyses identified R1400, R1284, R1173, and F1395 as key residues stabilizing cofactor binding in the FNR-like unit. RMSF analysis revealed low flexibility of these residues, supporting structural integrity. Correlation and free energy calculations further demonstrated their critical contributions to the energy landscape. In silico site-directed mutagenesis of these residues induced significant free energy changes, confirming their role in modulating domain dynamics. Notably, R1400 and F1395, previously associated with the calmodulin-binding domain, influence conformational flexibility, while R1173 represents a novel interaction hotspot distinct from R1284.

**Discussion:**

These results provide detailed insights into the dynamic mechanisms of the FNR-like unit and identify promising targets for the development of improved nNOS inhibitors to control excessive NO production in neurological disorders such as ischemic stroke.

## Introduction

Nitric oxide (NO), a key signaling molecule in the nervous system, exerts neuroprotective or neurotoxic effects depending on the nitric oxide synthase (NOS) subtype, disease stage, and cellular source (Calabrese et al., 2007). Overactivation of neuronal NOS (nNOS) and induction of inducible NOS (iNOS) contribute to brain damage post-cerebral ischemia (Wieronska et al., 2021). Excessive NO production is linked to neurotoxicity, particularly through interactions with N-methyl-D-aspartate receptors (NMDARs), a critical focus for ischemic stroke research. Our prior studies confirmed that global ischemia–reperfusion activates NMDARs (Zhou et al., 2008; Brito et al., 2010). Additionally, the NOD-like receptor pyrin domain-containing protein 3 (NLRP3) inflammasome is implicated in anti-NMDAR encephalitis, with our recent work highlighting the N-lobe of thioredoxin-interacting protein (TXNIP) as key for allosteric NLRP3 regulation, suggesting potential TXNIP/NLRP3 inhibitors (Cheng and Wang, 2022; Chiarini et al., 2023). NO also modulates signaling via S-nitrosylation, a modification linked to neurodegenerative diseases (Nakamura et al., 2013). Regulating NO levels is thus vital for studying neurological disorders, necessitating a deeper understanding of nNOS structure to control its activity and elucidate its roles in health and disease.

The nNOS enzyme exists as a homodimer, with each monomer comprising a PDZ domain, an oxygenase (heme) domain, a calmodulin-binding domain (CaMBD), and a reductase domain (Zhou and Zhu, 2009). As the oxidation process occurs in the heme domain, the reductase domain is essential for transferring electrons to sustain the reaction. Consequently, the reductase domain is of paramount importance, yet it has received less attention compared to other domains. The reductase domain of nNOS, located at the C-terminal region, is a modular structure containing subdomains that bind flavin mononucleotide (FMN), flavin adenine dinucleotide (FAD), and nicotinamide adenine dinucleotide phosphate (NADP(H)), analogous to that observed in cytochrome P450 (Hubbard et al., 2001). These subdomains are structurally and functionally organized to mediate a sequential electron transfer pathway from NADP(H) to FAD, FMN, and ultimately to the heme in the oxygenase domain of the partner monomer. The reductase domain of nNOS adopts a complex architecture, characterized by two key subdomains: the FMN-binding subdomain (approximately residues 750–948) and the FAD-binding subdomain (approximately residues 949–1,235, including the connection domain), with the latter also encompassing the NADP(H)-binding region (approximately residues 1,200-1,413). The FMN-binding subdomain adopts a flavodoxin-like fold, while the FAD-binding subdomain, often referred to as the FNR-like unit due to its structural homology with ferredoxin-NADP^+^ reductase (FNR), features a Rossmann-like fold that coordinates FAD and facilitates hydride transfer from NADP(H) (Iyanagi, 2022). The active sites of these subdomains are defined by conserved residues that stabilize the cofactors. For instance, the FAD-binding site includes aromatic and polar residues that anchor the isoalloxazine ring, while the NADP(H)-binding site features specific motifs for pyrophosphate and adenine recognition. The FMN-binding site, similarly, relies on hydrogen-bonding networks to secure the flavin cofactor, enabling electron shuttling to the heme. The FNR-like unit within the reductase domain is pivotal for initiating electron transfer by accepting electrons from NADP(H) and transferring them to FAD (Garcin et al., 2004). Structurally, this unit contains a *β*/*α*-barrel fold typical of FNR-like proteins, with key residues coordinating FAD binding and stabilizing its redox-active isoalloxazine ring. Previous studies have shown that electrons are sequentially transferred from NADP(H) to FAD and then to FMN during the reaction (Adak et al., 1999). Additionally, the CaMBD domain may interact with residues near the C-terminal tail, such as F1395, potentially influencing the process by suppressing electron transfer (Adak et al., 2002; Konas et al., 2004). Furthermore, R1284, a residue within the NADP(H) binding domain, helps neutralize the negative charges of NADP(H) phosphates, while R1173 may form a hydrogen bond with FAD (Garcin et al., 2004; Tiso et al., 2005). However, current structural models of nNOS, primarily derived from crystallographic studies, are static and provide limited insight into the dynamic interactions at the amino acid level under physiological conditions, such as in solution at room temperature. Those residues lack detailed characterization of their structural mechanisms, which may hinder further drug design efforts targeting them. Current ligand parameters for NADP(H), FAD, and FMN used in molecular dynamics simulations are often derived from older, smaller basis sets (like 6-31G), limiting their accuracy and necessitating updated parameters for precise modeling. Moreover, the full-length nNOS structure remains incomplete, raising the possibility that other domains, such as the oxygenase or calmodulin-binding regions, may interact with the reductase domain or the FNR-like unit, potentially modulating electron transfer efficiency.

For drug design targeting nNOS, such as in neurodegenerative diseases or conditions involving excessive NO production, elucidating these state-dependent interactions at the residue level is essential. Several inhibitors, such as spermidine (Spe) or N-monomethyl-L-arginine (L-NMMA) which targets NOS or specifically nNOS have yet to achieve clinical approval, indicating the presence of critical unresolved issues that are likely closely associated with unique structural interactions within nNOS (Hu et al., 1994; Reif and McCreedy, 1995). To address these challenges, this study investigates the dynamic interactions between the nNOS reductase domain and its cofactors (NADP(H), FAD, and FMN) in their reduced states. Using the larger def2-TZVP basis set, we updated RESP charge parameters and force constants for NADP(H), FAD, and FMN to enhance simulation accuracy. By constructing three computational models based on the electron transfer sequence, we employed molecular dynamics simulations to explore the equilibrium states of the reductase domain when each cofactor is in its reduced form. Our approach provides details about the key amino acid residues critical for cofactor binding of nNOS, while also examining the influence of nNOS-specific structural elements, such as the calmodulin-binding region or adjacent domains, on the FNR-like unit. These findings aim to provide a comprehensive understanding of the structural mechanisms underlying nNOS function, with implications for targeted therapeutic development.

## Results

### nNOS inhibitors partially inhibit LPS-induced NO release and NLRP3 activation but fail to normalize nNOS activity in cerebral ischemia–reperfusion injury

**Figure 1:**
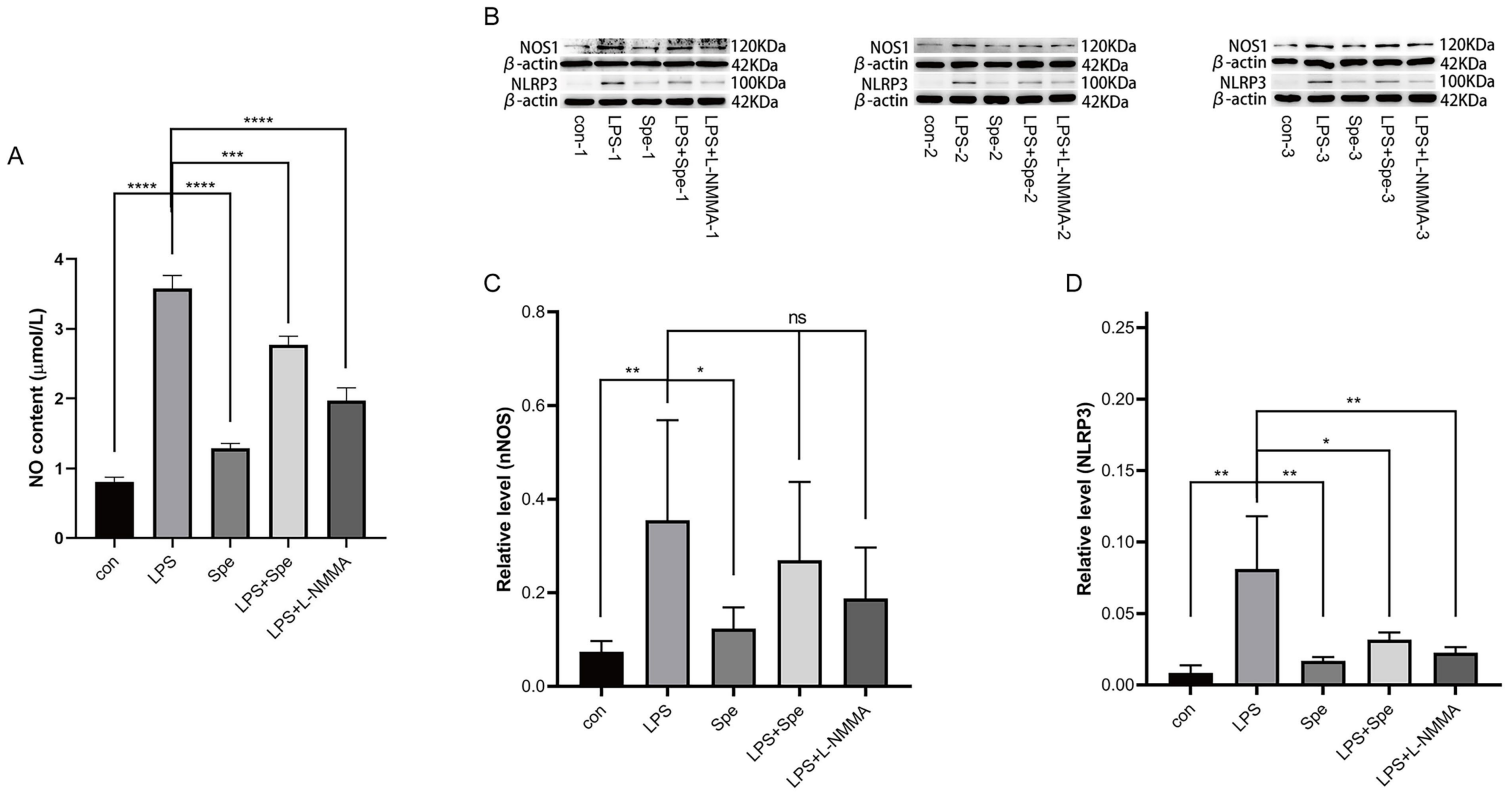
**(A)** Measurement results of NO content in cells of each group after adding Con (normal saline), LPS (lipopolysaccharide), Spe (nNOS specific inhibitor), LPS + Spe (lipopolysaccharide + nNOS specific inhibitor), and LPS + L-NMMA (lipopolysaccharide + Nω-methyl-L-arginine) in mouse neuronal cell line culture. **(B)** Western blot detection of nNOS and NLRP3 protein expression in cells with different treatments. **(C)** Relative gray values of nNOS protein bands in cells with different treatments detected by Image-Pro Plus. **(D)** Relative gray values of NLRP3 protein bands in cells with different treatments detected by Image-Pro Plus; ns: No significance, *p < 0.05, **p < 0.01, ***p < 0.001.

To evaluate the regulatory effect of nNOS protein inhibitors on nNOS activity, we selected widely used nNOS inhibitors, including the specific inhibitor spermidine (Spe) and L-NMMA inhibitor. Experiments were performed on mouse neuronal cell lines to observe their effects on nNOS activation and nitric oxide (NO) release in neurons (Figure 1A). The results showed that, using the LPS group as a benchmark, the addition of the two inhibitors (LPS + Spe/LPS + L-NMMA) significantly reduced cellular NO content after culture (*p* < 0.001). However, compared with the control (con) group under physiological conditions, the NO content in the LPS + Spe/LPS + L-NMMA groups remained excessively high. Western blotting was used to detect the expression levels of nNOS and NLRP3 (Figure 1B) to assess their effects on nNOS activity and downstream NLRP3 production. As observed in Figure 1C, compared with the LPS group, both the LPS + Spe and LPS + L-NMMA groups exhibited certain inhibitory effects on nNOS expression, but these effects were not statistically significant. In contrast to the con group, the addition of the Spe inhibitor unexpectedly increased the expression levels of both nNOS and NLRP3. The results for the expression level of the downstream inflammatory factor NLRP3 (Figure 1D) showed that, compared with the LPS group, both the LPS + Spe and LPS + L-NMMA groups significantly inhibited NLRP3 expression. However, compared with the con group, the NLRP3 expression levels in these two groups did not return to normal levels. In conclusion, nNOS inhibitors exhibit certain positive effects in alleviating cerebral ischemia–reperfusion injury. However, current inhibitors fail to appropriately suppress nNOS activity to a normal level, thus remaining unable to fundamentally relieve neurological damage. Therefore, we expect that studies on the electron transfer process in the reductase domain of the nNOS-mediated NO metabolic pathway will provide new directions and targets for designing nNOS activity inhibitors with excellent specificity and low toxic side effects in the future, and offer insights for developing drugs targeting nNOS hyperactivation for the treatment of neuropsychiatric diseases.

### Molecular dynamics simulations elucidate conformational changes in the nNOS reductase domain across different redox states of small molecules

**Figure 2:**
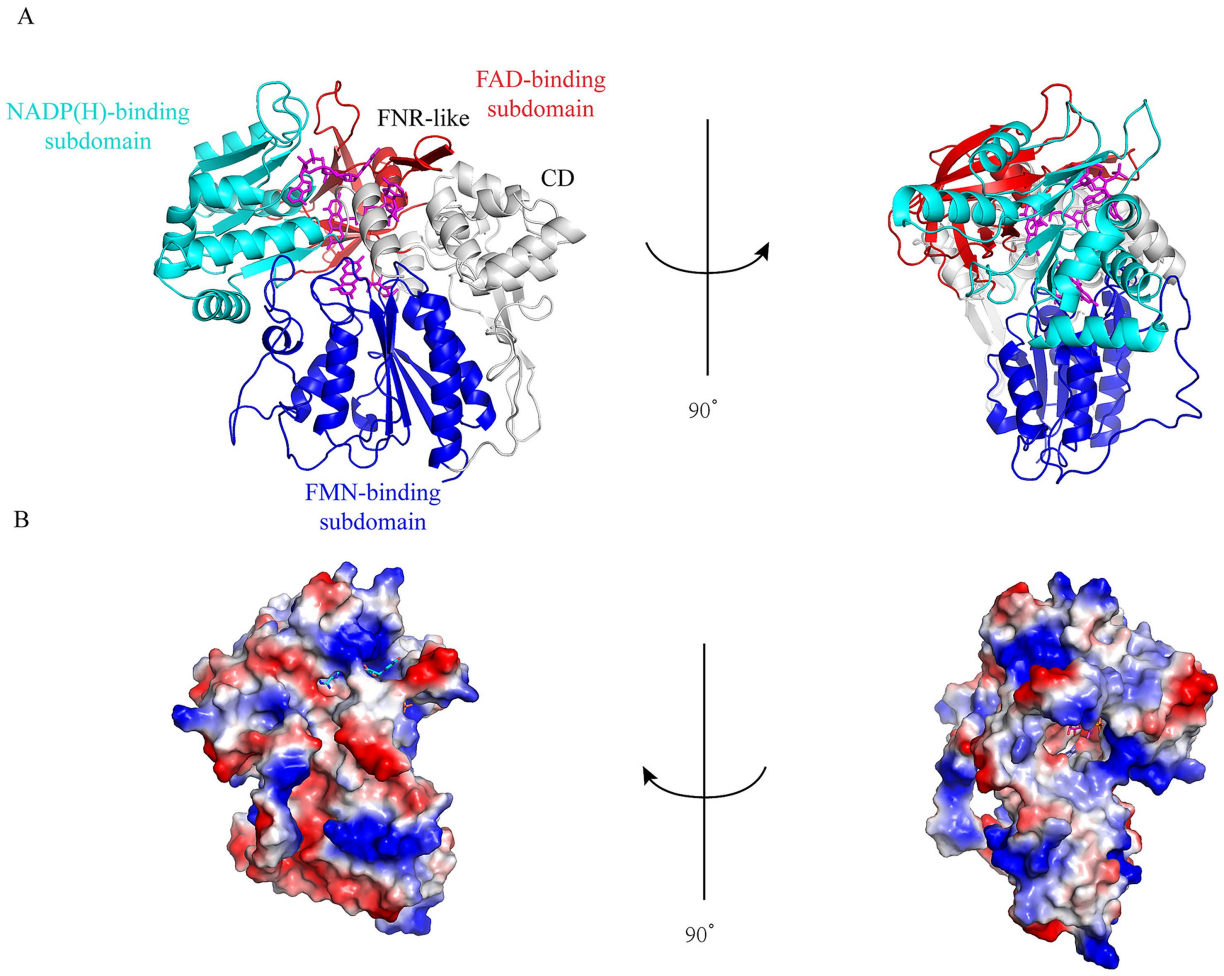
Structural and electrostatic analysis of the nNOS reductase domain. **(A)** Ribbon representation of the rat nNOS reductase domain (PDB ID: 1TLL), depicting front and left views with NADP(H), FAD, and FMN embedded within the protein matrix, highlighting their spatial arrangement critical for redox state transitions. **(B)** Vacuum electrostatic potential surfaces of the protein component (excluding the connection domain) in front and right views, illustrating the encapsulation of cofactors essential for electron transfer processes.

Due to the challenges of simulating electron movement in the reductase domain using molecular dynamics (MD), and considering that the reductase domain comprises three small molecules—each capable of existing in the reduced/oxidized state—we developed MD models representing three distinct states to investigate the behavior of these molecules in different redox states (Figure 2A). The vacuum electrostatic potential of the protein component of the reductase domain, excluding the connection domain (CD), was calculated. As depicted in Figure 2B, the small molecules are encapsulated within the protein structure, resembling a box-like enclosure. The first model (model 1) represents the scenario where NADP(H) is in its reduced state, with FAD and FMN remaining in their oxidized states. The second model corresponds to FAD in its reduced state (FADH₂), while the third model depicts FMN in its reduced state (FMNH₂). The Connection Domain (CD) was incorporated into the FAD-binding domain to ensure analytical continuity. To enhance the accuracy of MD simulations for the nNOS reductase domain, we employed Gaussian to compute the Restrained Electrostatic Potential (RESP) charges for each small molecule using the def2-TZVP basis set. The RESP charges for NADP(H), FAD, and FMN in their oxidized and reduced states were calculated using Multiwfn. Subsequently, VFFDT software was utilized to generate the necessary charge and force constants for parameters.

**Figure 3:**
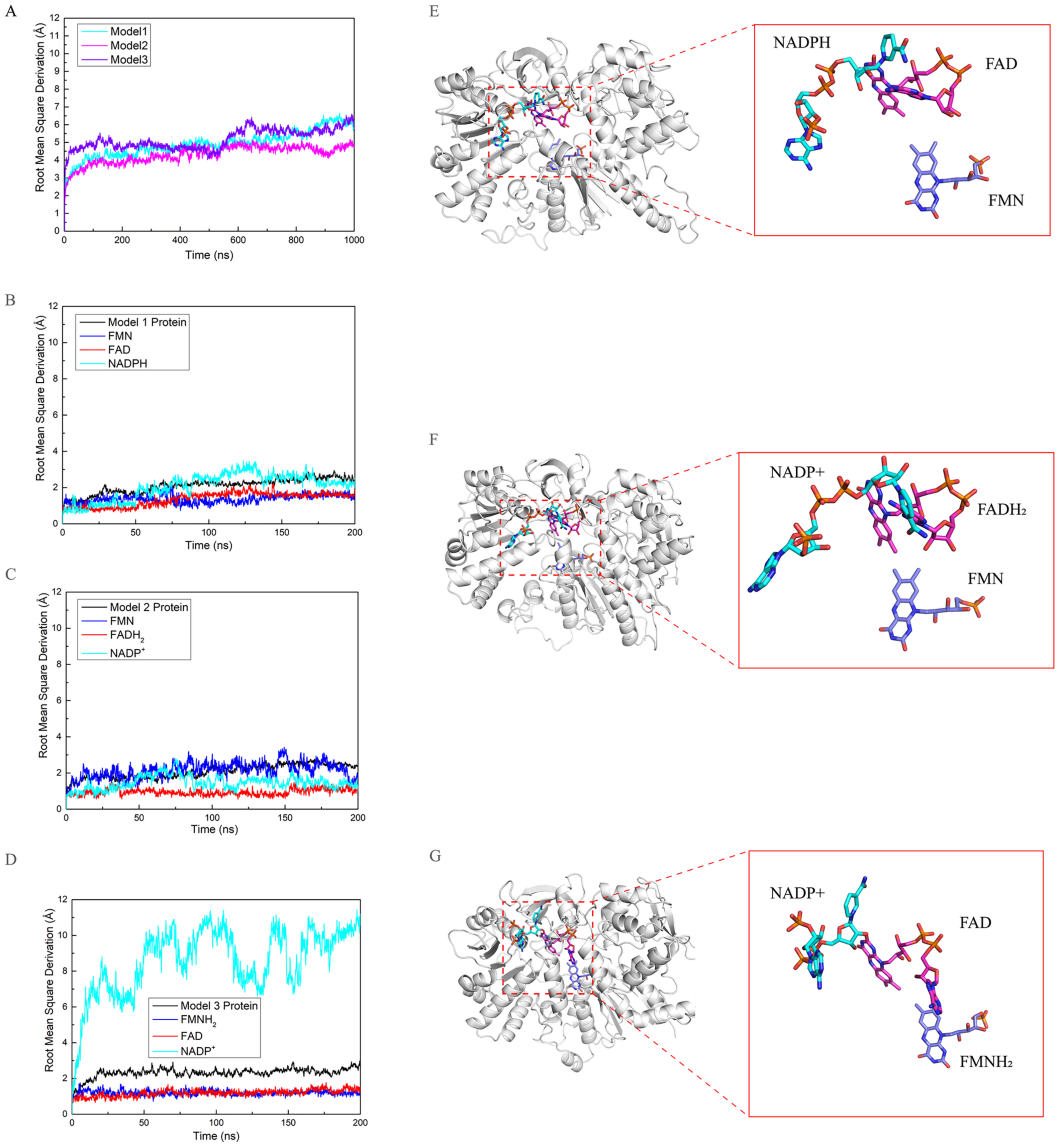
Conformational dynamics of the nNOS reductase domain across different redox states. **(A)** Root-mean-square deviation (RMSD) plots as a function of simulation time showing the global conformational stability of Model 1 (cyan), Model 2 (magenta), and Model 3 (violet). **(B–D)** RMSD plots as a function of simulation time for the protein backbone (black), FMN (violet), FAD (magenta), and NADP(H)/NADP^+^ (cyan) in Model 1 **(B)**, Model 2 **(C)**, and Model 3 **(D)**, respectively. **(E–G)** Representative equilibrium conformations (left) and superimposed selected trajectory frames (right) of NADP(H)/NADP^+^, FAD, and FMN in Models 1 **(E)**, 2 **(F)**, and 3 **(G)**, illustrating the overall structural organization of the protein and key conformational changes associated with the redox states of the cofactors.

One representative conformation was extracted from each state, designated as Model 1, Model 2, and Model 3, respectively. Molecular dynamics simulations were conducted to evaluate the stability of these models. Root-mean-square deviation (RMSD) analyses (Figures 3A–D) demonstrated that all three models achieved equilibrium after approximately 1.0 μs. Additional 200 ns simulations were employed to ensure the stability of the trajectories utilized for subsequent analysis. In Model 1, the RMSD of NADP(H) exhibited a notable increase from 2 Å to 2.5 Å at approximately 100 ns, maintaining this conformation until the simulation reach to 200 ns. This indicates a minor conformational change in NADP(H), while the RMSD of FMN and FAD remained relatively stable. In Model 2, NADP^+^ showed no increase on the RMSD, while FADH₂ and FMN exhibited almost the same during the production simulation. FMN displayed several fluctuations on the whole simulation, suggesting a some small transitions during its binding. In Model 3, the RMSD curves of the small molecules stabilized after reaching equilibrium, indicating that the system attains a relatively stable state following the transition to the FMN reduced state. However, the RMSD of NADP^+^ exhibits a sharp increase and pronounced fluctuations exceeding approximately 6 Å, indicating a relatively unstable conformational state. Independent molecular dynamics (MD) simulations of the three models revealed that, in Model 3, NADP^+^ exhibits a pronounced tendency toward dissociation from the nNOS reductase domain, as evidenced by unstable RMSD values and progressive displacement in parallel runs. In contrast, the other cofactors (FAD and FMNH₂) remained stably bound throughout the simulations, displaying only minor fluctuations. This differential behavior highlights a clear trend of binding instability for NADP^+^ specifically in the FMN-reduced state (Supplementary Figure S1). This may be associated with the reduced state of FMN, which could lead to the reductase domain exhibiting significant repulsion of the NADP^+^ molecule. Throughout the changes in redox states from NADP(H) to FMN, the distances between the three small molecules, particularly their ring structures, appeared to decrease(Figures 3E–G). We hypothesize that these ring structures are critical for determining the redox states of the molecules, facilitating transitions through closer contact. Additionally, the equilibrium conformation of FAD in Models 1 and 2 at approximately 1.0 μs differed significantly from that in Model 3 at the same time point. Specifically, the adenine and riboflavin moieties of FAD transitioned from an acute angle of approximately 30° to an obtuse angle of about 100° at the 0.5 μs time point. We propose that this structural change facilitates FAD’s role in transitioning between redox states involving NADP(H) and FMN.

### Analysis of key residues and molecular interactions in the nNOS reductase domain

**Figure 4:**
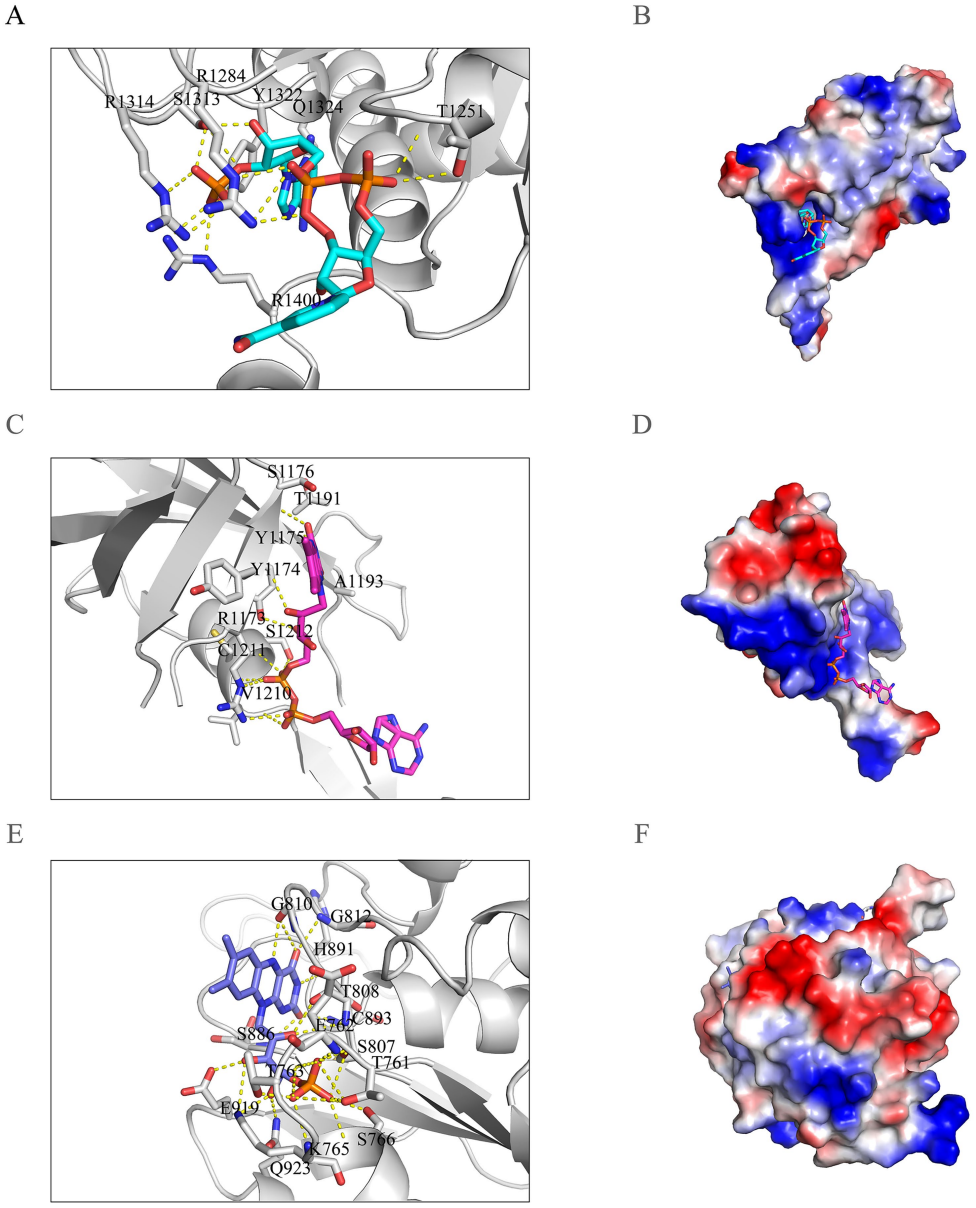
Polar contact analysis in the nNOS reductase domain. **(A,C,E)** Key residues forming intermolecular polar contacts in Model 1 (e.g., R1400, R1284), Model 2 (e.g., R1173, S1176), and Model 3 (e.g., H891, E762), with protein residues labeled in black to highlight their roles in cofactor interactions. **(B,D,F)** Vacuum electrostatic potential surfaces depicting protein interactions with NADP(H), FAD, and FMN in Models 1, 2, and 3, respectively, emphasizing the electrostatic environment facilitating cofactor binding.

To investigate the key residues involved in the transitions between different redox states of the three small molecules and to elucidate the potential causes of variations in the root-mean-square deviation (RMSD) curves, we analyzed polar contacts and hydrogen bond statistics across the models. Polar contacts exhibited notable differences due to variations in the molecular interfaces (Figures 4A–F). In Model 1, residues Q1324, Y1322, S1313, R1314, R1284, T1251, and R1400 were identified as potentially critical for the transitions between redox states. In Model 2, residues S1176, T1191, A1193, S1212, C1211, R1173, Y1174, Y1175, and V1210 were determined to play essential roles in the transitions between redox states. In Model 3, residues H891, G812, C893, G810, S886, T808, S807, Q923, E762, K765, T763, T761, S766, and E919 were found to be significant for the binding of the ligands.

**Figure 5:**
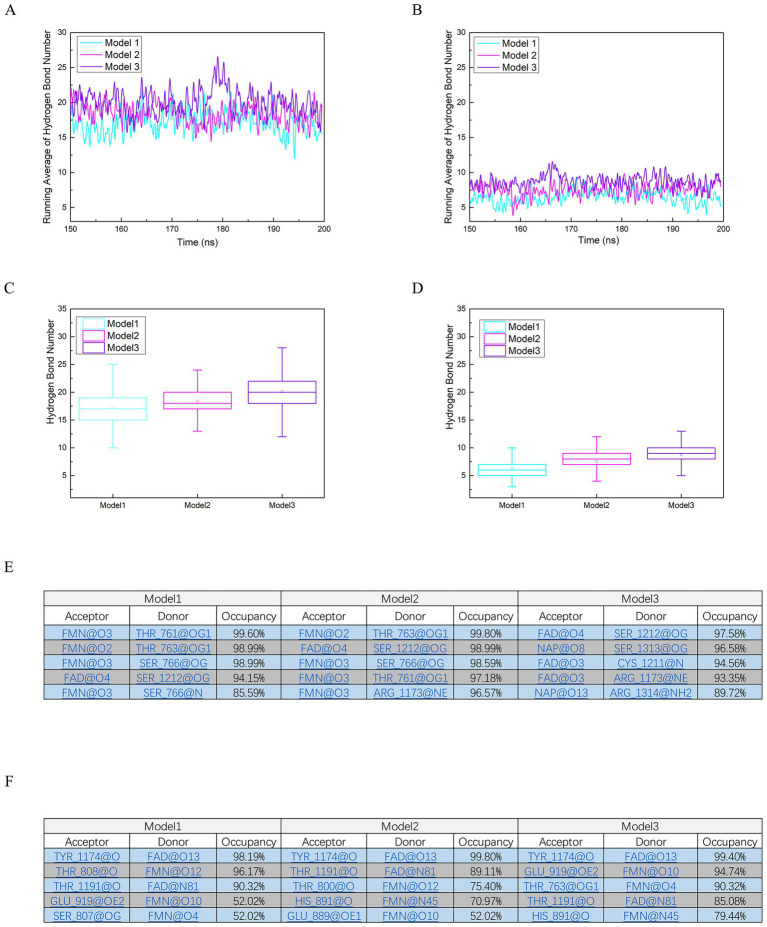
Hydrogen bond interactions in nNOS reductase domain models. **(A)** Running average of hydrogen bond counts with NADP(H), FAD, and FMN as donors and protein residues as acceptors for Models 1, 2, and 3, calculated over 496 frames post-1.0 μs equilibration. **(B)** Running average of hydrogen bond counts with protein residues as donors and cofactors as acceptors for Models 1, 2, and 3, post-1.0 μs equilibration. **(C,D)** Box plots of hydrogen bond counts for configurations in **(A)** and **(B)**, respectively, with whiskers at 1.5 times the interquartile range and boxes spanning the 25th to 75th percentiles, illustrating interaction stability. **(E,F)** Top five hydrogen bond interactions in Models 1, 2, and 3, with **(E)** cofactors as acceptors and **(F)** protein residues as acceptors, highlighting stable hydrogen bond networks critical for cofactor stabilization.

In addition to polar contacts, the three models demonstrated relatively stable hydrogen bond interactions, which are essential for maintaining the structural integrity and facilitating the transitions between different redox states of NADP(H), FAD, and FMN. Hydrogen bond statistics were calculated using a cutoff distance of 3 Å and a cutoff angle of 135°, ensuring a rigorous assessment of these non-covalent interactions. To comprehensively characterize the hydrogen bond networks, two distinct groups were defined: one where NADP(H), FAD, and FMN served as hydrogen bond acceptors and the surrounding protein residues acted as donors (Figures 5A,C,E), and the other where the protein residues were the acceptors and the cofactors were the donors (Figures 5B,D,F). The running average of hydrogen bond counts, computed 495-frame intervals following the initial 1.0 μs of simulation to capture equilibrated dynamics, revealed consistent patterns across the models (Figures 5A,B). Specifically, in the cofactor-to-protein donor-acceptor configuration, Model 3 exhibited the highest average hydrogen bond counts (approximately 20 bonds), reflecting a denser network likely due to the reduced state of FMN, which may enhance donor capacity through additional hydrogen networks. In contrast, Models 1 and 2 showed slightly lower averages (around 15–18 bonds), potentially attributable to the reduced forms of NADP(H) and FADH_2_, respectively, which could alter the availability of donor sites as electrons are redistributed. For the protein-to-cofactor configuration, the averages were comparably stable but marginally lower overall (5–10 bonds), suggesting a directional preference where cofactors more frequently act as acceptors, possibly to stabilize the protein scaffold during redox transitions. Box plot analyses further underscored the robustness and variability of these interactions (Figures 5C,D). In the cofactor-donor group (Figure 5D), the interquartile ranges (25th to 75th percentiles) were narrow across all models (e.g., 5–7 bonds for Model 1), indicating low fluctuation and high stability post-equilibration. This stability implies that hydrogen bonds in this orientation contribute to a resilient framework that resists conformational perturbations during redox state shifts. The protein-donor group (Figure 5C) displayed similar trends but with slightly broader interquartile ranges in Model 3 (e.g., 17–27 bonds), hinting at increased variability near FMNH₂, which may correlate with the elevated root-mean-square fluctuation (RMSF) observed in the FMN-binding region (as noted in subsequent analyses). These distributions highlight the role of hydrogen bonds in buffering dynamic changes, ensuring that redox-active sites remain optimally positioned.

Examination of the top five hydrogen bond interactions in each model provided granular insights into specific residue-cofactor pairings (Figures 5E,F). In the cofactor-donor configuration (Figure 5E), recurrent interactions included FMN@O3 – THR_761@OG1 in Model 1 (occupancy 99.6%) and FMN@O2 – THR_763@OG1 in Model 2 (occupancy 99.8%), underscoring THR_761 and THR_763’s pivotal roles in stabilizing FMN through frequent bonding, which aligns with their identification in polar contact analyses. Similarly, in Model 3, bonds such as FAD@O4 – SER_1212@OG exhibited high occupancy (97.58%), reinforcing SER_1212’s contribution to FAD anchoring. For the protein-donor group (Figure 5F), interactions like TYR_1,174@O – FAD@O13 in Model 2 (occupancy 99.8%) and THR_808@O – FMN@O12 in Model 1 (occupancy 96.17%) were prominent, indicating that polar residues such as tyrosines and threonines frequently donate hydrogens to cofactor oxygens, potentially facilitating electron redistribution by modulating local electrostatics. These high-occupancy bonds, often exceeding 90% across models, suggest a conserved mechanism where hydrogen bonding networks dynamically adapt to redox states of the three ligands, with certain residues (e.g., threonines and serines as donors or acceptors) acting as hubs to enhance cofactor-protein affinity. Overall, the sustained high levels of hydrogen bonds under all conditions emphasize their crucial function in orchestrating the precise alignment necessary for efficient redox state transitions within the nNOS reductase domain.

### The flexibility of critical residues remains low, while a *α*-helix appears to be significant

**Figure 6:**
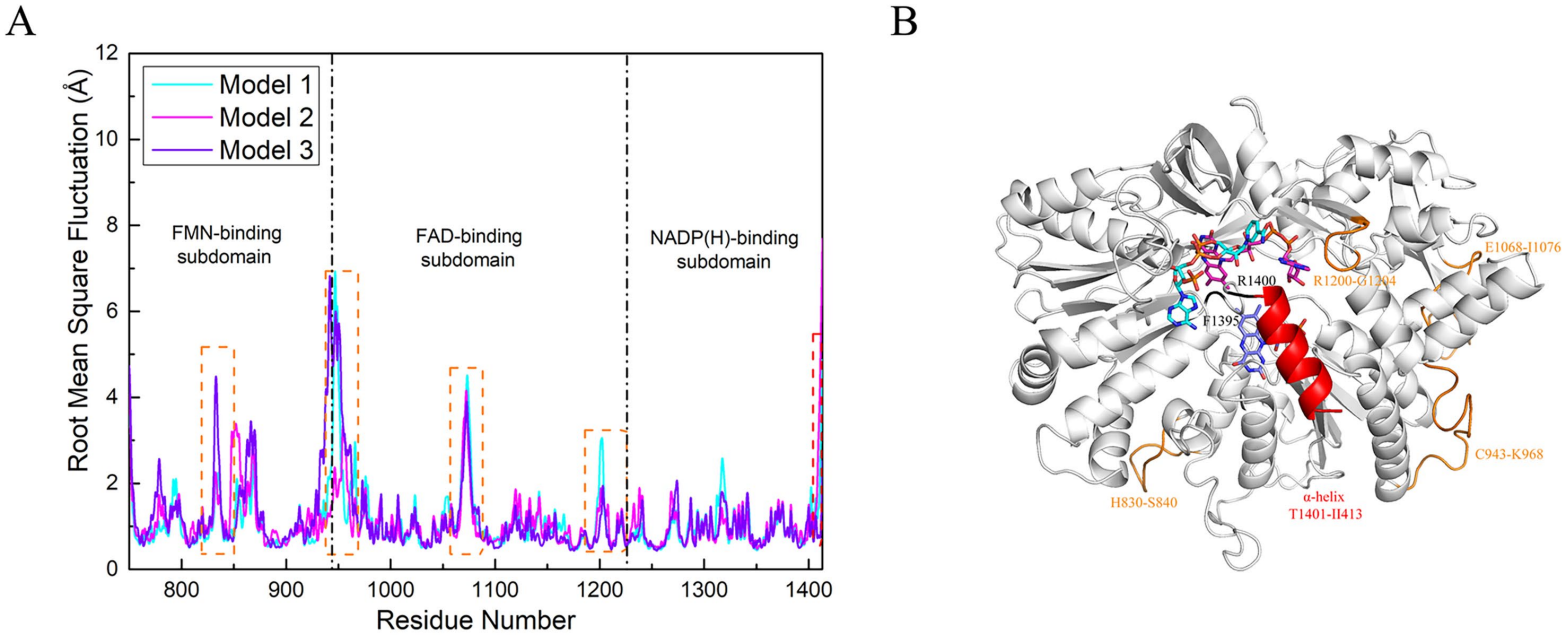
Residue flexibility analysis in the nNOS reductase domain. **(A)** Root-mean-square fluctuation (RMSF) profiles for models 1 (cyan), 2 (magenta), and 3 (violet), with high-flexibility regions (red dash lines) indicating the *α*-helix, and the part enclosed by the orange dash line represents loop and peripheral residues. **(B)** Structural representation of the α-helix residues (red), located proximal to cofactors, suggesting a regulatory role. The structural representation of R1400 and F1395 is marked in black. The structural representation of loop and peripheral residues is marked in orange.

To further investigate the structural dynamics of the nNOS reductase domain during electron transfer among the small molecules, we calculated the root-mean-square fluctuation (RMSF) of the reductase domain (Figure 6A). The RMSF peaks corresponding to the *α*-helix in the C-terminal tail (CTN) segment (residues T1401–I1413) are highlighted with red dashed boxes, while those associated with four segments of loop and peripheral residues are marked with orange dashed boxes (H830–S840, C943–K968, E1068–I1076, and R1200–G1204). The α-helix is colored red, the four loop segments and peripheral residues are colored orange, and residues R1400 and F1395 are highlighted (Figure 6B). Analysis of the RMSF plot reveals prominent peaks at the C-terminal end of the *α*-helix within the CTN segment, indicating that this helix plays a key role in driving conformational changes and regulating electron transfer during the reaction process. Inspection of the complete PDB structure of the reduced domain shows that R1400, located in the CTN segment, is in close proximity to and interacts with the *α*-helix, consistent with prior hypotheses that R1400 modulates the conformation of this domain. The relatively modest RMSF peak for R1400 likely reflects its position near the core of the molecular domain, conferring lower flexibility compared to the distal end of the *α*-helix and suggesting greater conformational stability. Nevertheless, given the extensive polar interactions involving R1400 reported previously, it appears to serve as a critical regulatory site that influences biochemical reactivity by modulating conformational dynamics within the nNOS reductase domain. Similarly, F1395, situated at the C-terminal end of the NADPH-binding subdomain and linking the final *β*-strand of this subdomain to the CTN segment (including its α-helix), exhibits relatively low flexibility in the RMSF plot. Subsequent free energy calculations demonstrate strong van der Waals interactions involving F1395, and like R1400, it resides on the loop adjacent to the α-helix. Thus, F1395 may also regulate the reaction by influencing α-helix conformational changes. Prior studies have additionally implicated interactions between F1395 and the calmodulin-binding domain (CaMBD) in inhibiting electron transfer and modulating the overall reaction process.

Several peripheral loop regions (highlighted in orange) display substantial flexibility in the RMSF plot. Notably, residues R1200–G1204 are structurally proximate to the α-helix in the CTN segment, suggesting that this loop may indirectly modulate α-helix conformational changes and thereby contribute to the overall regulation of the nNOS reductase domain. The regions C943–K968 and E1068–I1076 largely belong to the FNR-like subdomain and are positioned on chain A of the nNOS reductase domain, in close spatial proximity to the corresponding regions on chain B. This arrangement implies potential inter-subunit interactions within the dimeric structure, which could influence the global conformation of the reductase domain and the electron transfer process. In contrast, the H830–S840 segment is distant from the active site, consistent with the general observation that peripheral residues often exhibit high flexibility due to fewer structural constraints. The elevated flexibility of these peripheral regions underscores the relative stability (low flexibility) of residues proximal to the three small molecules. Furthermore, across all three models, the RMSF values for residues adjacent to the reduced-state small molecules are markedly higher than those in the other states. Figure 6A illustrates the approximate distribution of amino acid regions associated with each small molecule. In the FMN-binding subdomain, the RMSF curve for residues surrounding FMN (highlighted in purple) shows a notably elevated peak compared to the other curves. These findings suggest that conformational flexibility is enhanced in residues proximal to charged small molecules, implying that specific residues play a critical role in stabilizing the cofactors. This enhanced flexibility may also account for the increased residue mobility around FMN in its reduced state in model 3, potentially facilitating the displacement and dissociation of NADP.

### Structural dynamics and residue flexibility in the nNOS reductase domain

**Figure 7:**
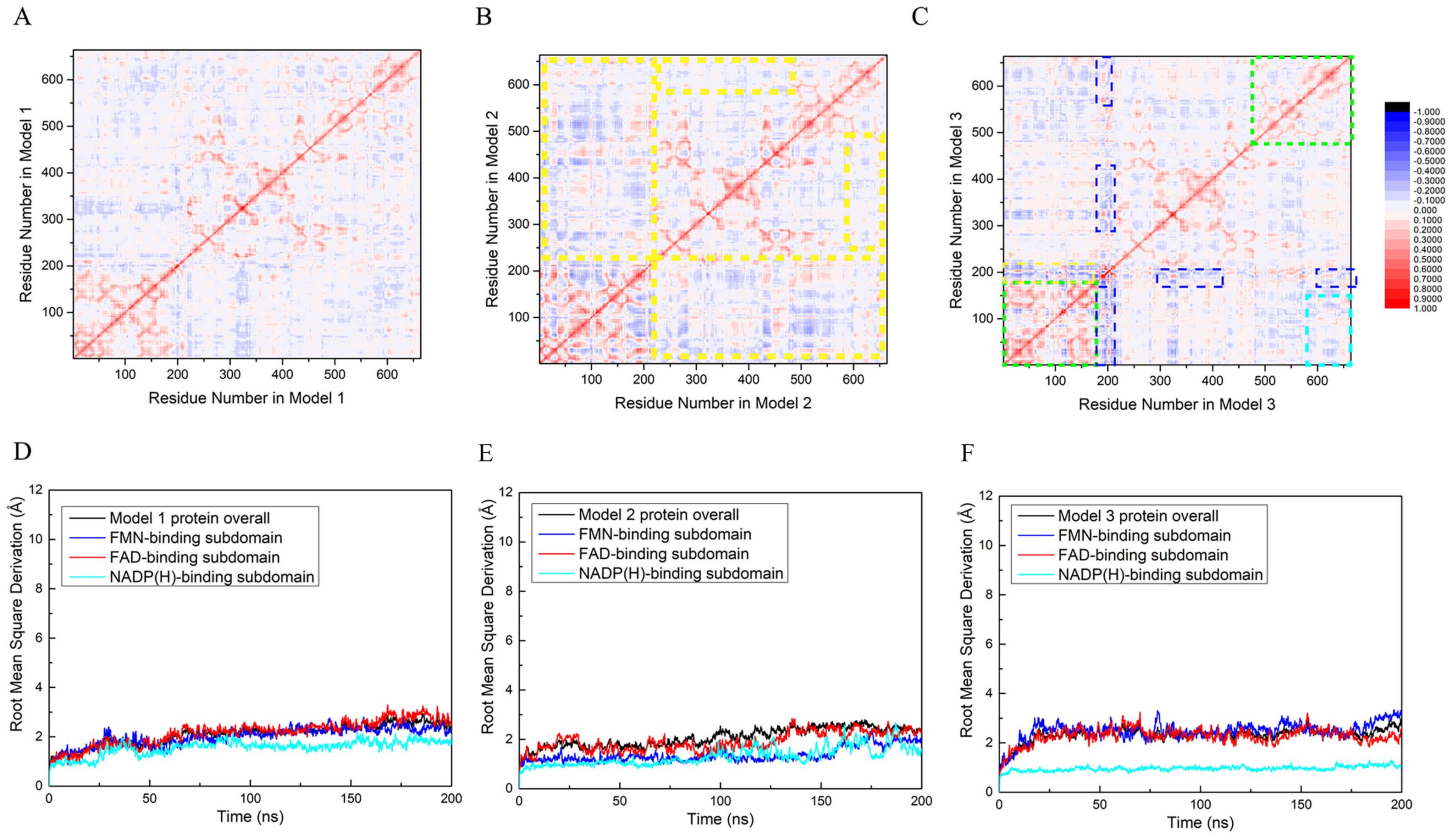
Correlation analysis of residue dynamics and subdomain stability in the nNOS reductase domain. **(A–C)** Correlation factor analysis for 667 residues in models 1, 2, and 3, respectively, illustrating coordinated motions between protein residues and cofactors during redox state transitions. **(D–F)** RMSD curves for the overall protein structure and subdomains in models 1, 2, and 3, demonstrating stable subdomain motions unaffected by internal fluctuations.

Subsequently, we calculated the residue-to-residue correlation factors that reflect the overall motion patterns of amino acid residues surrounding the cofactors in the three models (Figures 7A–C). We focus on residues that are positively and negatively correlated with the three cofactors. To confirm that these positive and negative correlations are not due to internal motions within the subdomain structures, we calculated the RMSD for each subdomain in all models, revealing that their motions exhibit a stable state (Figures 7D–F). Compared with model 1, the red and blue portions are more pronounced in models 2 and 3. This is presumed to be due to progressively more distinct relative motions of surrounding amino acid residues as the redox state transitions between FAD and FMN. From model 3, we can clearly observe that the redox state changes of the cofactors influence the correlations among surrounding amino acids. Residues from about 750 to 940 (1 ~ 191 in the graph) exhibit enhanced positive correlations with each other, while residues at about 1,249 ~ 1,409 (500 ~ 660 in the graph) show slight positive correlation changes, which are boxed by green dashed boxes. Positive correlations of these subsections indicate that the motions of the three cofactors indeed influence corresponding residues. This phenomenon excellently represents the one-to-one correspondence between protein parts and the cofactors in the nNOS reductase domain. However, the area enclosed by the blue dashed line in model 3 shows more pronounced anti-correlation compared to model 1. This part primarily indicates an opposite motion trend between the CD region in the FAD-binding subdomain and the NADP(H), FAD, and FMN binding domains. In model 2, the square areas enclosed by the yellow dashed line show minor negative correlations compared to model 1. This part primarily indicates that the pairwise relationships between the binding domains of the three cofactors exhibit negative correlations. It suggests that protein parts related to different cofactors are relatively independent. The area boxed by cyan dashed rectangles also shows distinct correlation reversals in model 3, with positive correlations in models 1 and 2 shifting to negative correlations in model 3, corresponding to the FMN-binding domain and C-terminal tail residues. This suggests a possible structural interplay between the two protein parts.

### Free energy calculation and several mutation attempts provide a deeper insight into the essential residues in cofactor binding

**Figure 8:**
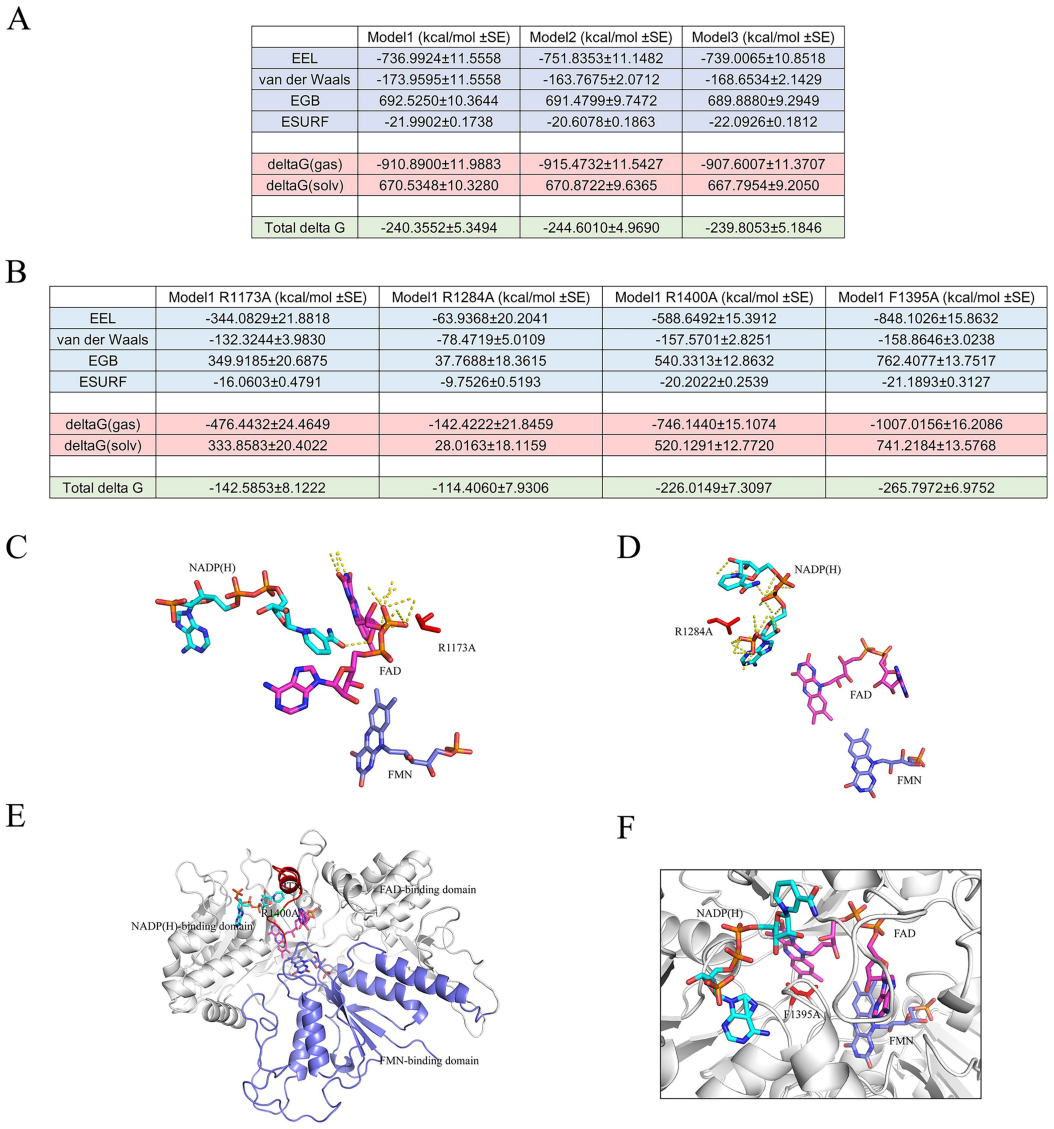
Free energy analysis and mutational effects. **(A)** MM/GBSA free energy calculations for Models 1, 2, and 3, showing a decrease in binding energy with predominant electrostatic contributions across redox states (SE: standard error). **(B)** Free energy decomposition for wild-type and mutant models (R1173A, R1284A, R1400A, F1395A) in Model 1, revealing reductions in electrostatic and van der Waals energies post-mutation. **(C–F)** Final trajectory frames of NADP(H), FAD, and FMN for mutants R1173A, R1284A, R1400A, and F1395A, respectively, illustrating altered cofactor conformations and interactions compared to the wild type. All units are in kcal/mol. All MM/GBSA calculations were performed using 196 snapshots extracted from the last 100 ns of each equilibrated trajectory.

Free energy calculations, employing the MM/GBSA method, offer approximate insights into the contributions of polar contacts and hydrogen bonds for individual residues or residue groups within the nNOS reductase domain during transitions between different redox states of the three small molecules, acknowledging the inherent uncertainties and approximations in MM/GBSA estimates (typically on the order of several kcal/mol due to factors such as solvent modeling and entropy contributions). Several lesser-known residues were tentatively identified as potentially critical to these redox state transitions. We analyzed a 50 ns segment from 150 ns to 199.6 ns in the extended 200 ns production simulations, designating the three small molecules as the ligand and the remaining protein components as the receptor. The results indicated a notable decrease in binding energy across all three models, which may suggest that the redox state transitions play an important role in the overall process, though this interpretation should be viewed cautiously given MM/GBSA’s limitations in capturing dynamic effects (Figure 8A). In all models, both van der Waals (vdW) and electrostatic interaction energy (EEL) appeared to contribute to the binding of cofactors. However, vdW showed a relatively large contribution, potentially implying that nonpolar contacts are a significant driving forces in these bindings.

Model 2 exhibited the highest estimated EEL value, which could indicate that its polar contacts are among the most prominent, albeit with potential over- or underestimation due to MM/GBSA approximations. The apparently greater contribution of vdW compared to EEL in Model 1 may further support the role of nonpolar contacts in facilitating the redox state transitions. As Model 1 represents the initial state in the redox transition, the extensive interaction surface between the molecules might account for the observed higher EEL, though alternative factors such as conformational sampling could also influence these values. Additionally, in the equilibrium state of Model 1, the aromatic rings of the three small molecules showed a tendency for interaction, which may contribute to the elevated vdW interactions. Through analysis of the energy decomposition (see Supplementary Files) for Model 1, we provisionally identified R1173, R1284, R1400, and F1395 as key residues in the molecular dynamics (MD) process. R1173 appeared to contribute the highest total energy, R1284 was a great contributor to EEL, and F1395 was the dominant contributor to vdW, based on the decompositions. R1400 also seemed to make notable contributions to both EEL and total energy and is part of the C-terminal tail residues, which exhibited elevated activity in prior root-mean-square fluctuation (RMSF) analyses. Based on the total energy decomposition analyses across all models, we selected Model 1 for site-directed mutagenesis to investigate the impact of residue alterations on energy profiles, as it also shows the initial state. Specifically, we introduced R1173A (mutation 1), R1284A (mutation 2), R1400A (mutation 3), and F1395A (mutation 4) mutations to elucidate their roles in modulating the energetic contributions to cofactor binding within the nNOS reductase domain. MD simulations of 1.0 μs were conducted on the mutated models, followed by MM/GBSA analysis of their last ~100 ns equilibrium trajectory (Figure 8B). Mutation 1 was associated with a marked reduction in EEL and, to a lesser extent, vdW, which is consistent with the hypothesis that R1173 primarily exerts electrostatic interactions, although MM/GBSA error margins warrant cautious interpretation. In mutation 2, both EEL and vdW appeared to decrease significantly, potentially aligning with R1284’s dominant contribution to EEL and vdW. The presence of multiple amino groups in arginine may explain the substantial vdW contribution. Similarly, mutation 3 showed apparent reductions in EEL but increase in vdW, which might reflect R1400’s strong electrostatic interactions and the opposite vdW contributions of arginine’s amino groups or other residues. In contrast, mutation 4 exhibited no apparent reduction in the total energy if standard errors are considered. Analysis of the energy decomposition of post-mutation revealed increased vdW contributions from other residues, such as TYR488 and TYR663, and from FAD. We hypothesize that the minimal change in total vdW in mutation 4 may result from F1395’s vdW contribution being an approximation of *π*-stacking interactions in the simulation. Examination of the frames of NADP(H), FAD, and FMN trajectories at ~0.5 μs in the mutated models provided additional observations. In mutation 1 (Figure 8C), the FAD conformation deviated from that of the wild type at equilibrium, with R1173A losing its polar contact with FAD, consistent with the wild-type interaction. In mutation 2 (Figure 8D), the NADP(H) conformation underwent significant changes, with an increased distance between NADP(H) and FAD, and R1284A lost its polar contact with NADP(H), aligning with prior findings. In mutation 3 (Figure 8E), the *α*-helix (C-terminal tail) deviated from the FMN-binding domain, suggesting that R1400 may regulate the C-terminal tail’s interaction with the FMN-binding domain. In mutation 4 (Figure 8F), FAD exhibited deformation compared to the wild type, with a reduced barrier between NADP(H) and FAD. This observation is consistent with previous reports indicating that the aromatic side chain of F1395 stacks with the isoalloxazine ring of FAD, potentially hindering the nicotinamide group of NADP(H) from accessing FAD and thus affecting redox state transitions. These findings collectively suggest that the four residues may significantly influence the transitions between different redox states in the nNOS reductase domain, though further experimental validation is recommended to account for MM/GBSA’s computational uncertainties.

## Discussion

**Figure 9:**
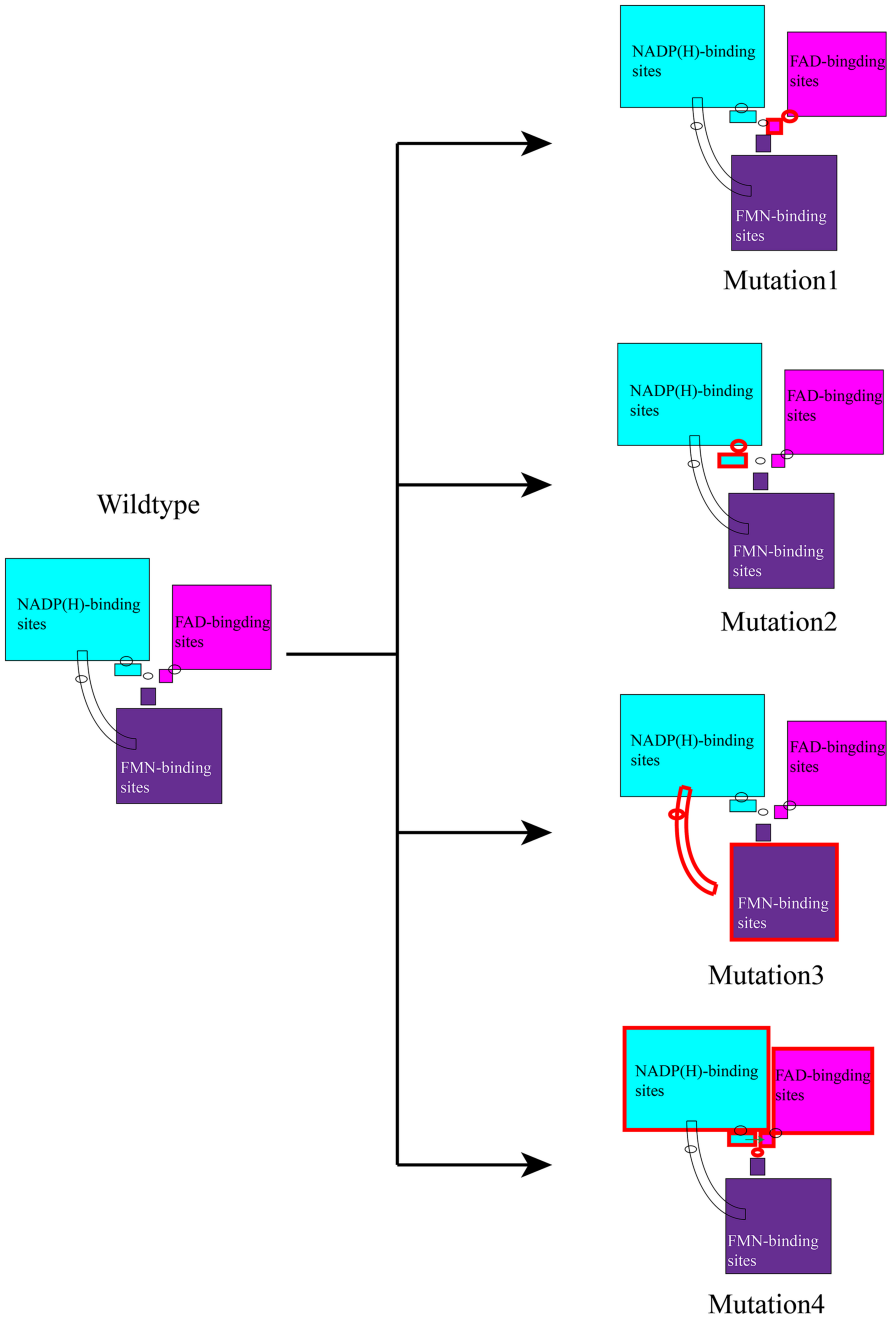
Structural implications of mutational analysis. Schematic representation of equilibrium conformations for mutations 1–4 after 1.0 μs MD simulations, highlighting the effects of mutations (R1173A, R1284A, R1400A, F1395A) on cofactor interactions and their implications for designing nNOS-targeted therapeutics.

This study evaluates the inhibitory capacities of two commonly used nNOS activity inhibitors and observes that although they exhibit certain positive effects in alleviating cerebral ischemia–reperfusion injury, current inhibitors fail to appropriately suppress nNOS activity to a normal level. This leads to increased production of the downstream inflammasome NLRP3, causing a series of side effects, and ultimately failing to fundamentally relieve neurological damage. Therefore, we expect that by investigating the structural characters in the reductase domain of the nNOS-mediated NO metabolic pathway, corresponding drug design targets can be identified. To achieve this, we generated three models of the nNOS reductase domain and conducted vacuum electrostatic calculations, polar contact analyses, hydrogen bond statistics, root-mean-square fluctuation (RMSF) calculations, correlation factor analyses, and free energy analyses on these models. We designed four mutation models and derived conclusions from them (Figure 9). To facilitate molecular dynamics simulations, we employed Gaussian, Multiwfn, and VFFDT programs to derive new parameters for NADP(H), FAD, and FMN, encompassing their oxidized and reduced states. The equilibrium conformations obtained from MD simulations not only represent some of the most stable configurations of the reductase domain under different redox states of the three small molecules but also highlight potentially important residues and their interactions with these cofactors. These findings offer insights for the design of relatively specific nNOS inhibitors and provide further understanding for improved management of nNOS-related neurological disorders.

In the nNOS reductase domain, electrons are generally transferred from NADP(H) to FAD and subsequently to FMN. While numerous studies have explored inter-domain relationships in nNOS, investigations into the processes within the reductase domain remain limited. The nNOS reductase domain, particularly its FNR-like unit, plays a critical role in stabilizing NADP(H) and FAD, facilitating cofactor interactions essential for nNOS function. The FNR-like unit, characterized by a Rossmann-like fold, coordinates FAD binding and supports the structural integrity of the reductase domain, as evidenced by stable equilibrium conformations in our MD simulations. However, the dynamics of electron transfer, occurring on a femtosecond (fs) timescale, are challenging to capture accurately in MD simulations due to their nanosecond resolution (Marcus and Sutin, 1985). Instead, our models reveal conformational adaptations in the reductase domain, such as a significant shift in FAD’s adenine-riboflavin angle from ~30° to ~100°, which likely optimizes the positioning of the FNR-like unit for cofactor interactions. We hypothesize that this alteration occurs because FAD serves as an intermediary in accepting electrons from NADP(H) and transferring them to FMN, which may account for the substantial equilibrium state variations in FAD across the models. We also noted that, in the 30° folded conformation, the adenine moiety of FAD may obstruct the redox-active sites of the three small molecules. RMSF analyses further indicate that residues within the FNR-like unit and adjacent to cofactors exhibit low flexibility, ensuring structural stability, while an *α*-helix in the C-terminal tail, including R1400, shows elevated flexibility, suggesting a regulatory role in the reductase domain’s dynamics.

Prior studies have established that residues R1400, R1284, R1173, and F1395 play significant roles in the catalytic process of the nNOS reductase domain, particularly within the FNR-like unit, but their structural mechanisms remain underexplored, with conclusions drawn from static crystallographic structures rather than dynamic analyses. Through our polar contact analyses and free energy calculations on dynamic structures, residues R1400, R1284, and F1395 were found to play prominent roles, aligning with previous research (Adak et al., 2002; Garcin et al., 2004; Konas et al., 2004; Tiso et al., 2005; Bignon et al., 2019). In particular, R1400 and F1395 have been documented in multiple studies. Previous work has indicated that R1400 interacts with the negatively charged 2′-phosphate group of bound NADP(H) via ionic contacts, subsequently modulating the equilibrium position of the FMN-binding domain through the C-terminal tail (CTN) to influence domain electron transport (Tiso et al., 2005). This is consistent with our energy decomposition results, polar contact findings, and mutant equilibrium conformations. Our simulations validate these prior perspectives and visually depict the potential equilibrium conformation of the reductase domain under the R1400A mutation. Similarly, F1395 has been reported to stack with the isoalloxazine ring of FAD, with the CaMBD domain also contributing to this process. Studies have noted that the activity of the CaM-free mutant resembles that of the wild type upon CaM binding, suggesting that mutant conformations could serve as a foundation for CaMBD-related investigations. Nitric oxide synthesis requires F1395 to swing toward the FMN domain, enabling the nicotinamide group to access FAD (Adak et al., 2002; Garnaud et al., 2004; Daff, 2010). We conducted the F1395A mutation to assess its van der Waals (vdW) energy contribution; post-simulation, a slight decrease in vdW energy decomposition was observed. This aligns with the use of vdW forces in MD simulations to approximate *π*-stacking interactions, confirming the presence of π-stacking for F1395, as evidenced by the proximity of its aromatic ring to the FAD isoalloxazine ring. Following the F1395A mutation, the barrier between NADP(H) and FAD diminished, consistent with the notion that this facilitates nicotinamide access to FAD for reaction progression, while wild-type F1395 represses this process. Prior studies have also indicated that the CaMBD domain alleviates F1395-mediated repression of electron transfer, an effect akin to that of the F1395 mutation. Given that the combined influence of CaMBD with R1400 and F1395 approximates the outcomes of R1400 and F1395 mutations, this reinforces our laboratory’s earlier conclusion that SUMO1 docking to CaMBD impacts residues near the FAD-binding domain (Wang and Hou, 2020). Additionally, R1173 exhibited largest total energy contribution in the decomposition analyses. Further breakdown revealed a substantial role for R1173’s electrostatic interaction energy (EEL). We performed the R1173A mutation, observing that R1173A lacked potential polar contacts with FAD, thereby confirming the polar interaction between R1173 and FAD. The MD and energy decomposition results for this mutant supported our speculation, showing a significant EEL reduction. These outcomes indicate that R1173 exerts a notable effect on the system, offering a novel reference point other than R1284. Previous perspectives have suggested that the positively charged R1173 forms hydrogen bonds with FAD’s pyrophosphate group (Zhang et al., 2001). Our hydrogen bond analyses also revealed frequent hydrogen bonds between ARG_1173@NE and FAD_1415@O3. Furthermore, R1284 assists in balancing the negative charges of NADP(H) phosphates and has been confirmed to interact with NADP(H) (Zhang et al., 2001; Garcin et al., 2004). This is substantiated by the high EEL for R1284 in both total binding energy and energy decompositions. The equilibrium conformation of the R1284A mutant, showing NADP(H) distanced from FAD, underscores its critical role. It is worth noting that the regulation of nNOS activity involves its dimerization and the influence of additional domains on the reductase domain, including its FNR-like unit. These complex processes contribute to intricate effects on ligand binding and chemical reactions. Given that the full-length three-dimensional structure of nNOS has not yet been resolved, such studies remain challenging. Future research may focus on integrating structural biology and molecular dynamics simulations to provide a more detailed elucidation of nNOS activity regulation, accounting for factors such as dimerization.

Our work employs computational chemistry to delineate the dynamic roles of these residues in stabilizing NADP(H), FAD, and FMN within the FNR-like unit and the broader reductase domain. We corroborated prior findings while uncovering novel insights, particularly the significant role of R1173 in FAD interactions. In contrast to studies on inter-domain electron transfer in nNOS, intra-domain processes represent an area requiring urgent exploration, as they hold academic and therapeutic interest for conditions like ischemic stroke. We reconstructed updated RESP charge parameters for NADP(H), FAD, and FMN using the def2-TZVP Gaussian basis set, enhancing the accuracy of future MD studies. Although our Gaussian-derived charges supported MD simulations, the precise pathways for transitions between the three small molecules warrant further exploration due to the limitations of MD in resolving fs-scale electron transfer. Our study identifies R1173/R1284 among R1400, R1284, R1173, and F1395 as promising potential drug targets for NO-related neurological disorders. These findings lay a solid structural foundation for the rational design of next-generation, highly selective nNOS inhibitors capable of more precisely restoring physiological NO levels — a critical step toward developing safer and more effective therapeutics for ischemic stroke, neurodegenerative diseases, and other conditions driven by nNOS overactivation in the coming years.

## Methods

### Model construction

The initial models were constructed based on the X-ray crystallographic structure of the nNOS reductase domain (PDB ID: 1TLL). Using Gaussian 16 (Frisch Frisch et al., 2016), partial atomic charges for three small molecules—NADP(H), FAD, and FMN—in their oxidized and reduced states were calculated at the B3LYP/def2-TZVP level of theory, ensuring convergence for all calculations (Lee et al., 1988; Becke, 1993; Weigend and Ahlrichs, 2005). The RESP (Restrained ElectroStatic Potential) charge parameters and force constants for these molecules were generated using Multiwfn (Lu and Chen, 2012) and VFFDT (Zheng et al., 2016) software. Three distinct models were developed to simulate different redox states of these small molecules by integrating the RESP charge parameters for their oxidized and reduced forms. Specifically, Model 1 (state 1) included NADP(H), FAD, and FMN; Model 2 (state 2) comprised NADP^+^, FADH₂, and FMN; and Model 3 (state 3) consisted of NADP^+^, FAD, and FMNH₂.

### Molecular dynamics simulations

Molecular dynamics (MD) simulations were performed using the AMBER 24 software package (Case et al., 2024). The protein was modeled with the AMBER ff19SB all-atom force field(Tian et al., 2019), while the small molecules (NADP(H), FAD, and FMN) were parameterized using the general AMBER force field (GAFF2) (Wang et al., 2004) combined with the RESP charges derived as described above. To maintain an ionic strength of 100 mmol/L, approximately 55 Na^+^ and Cl^−^ ions were added to each system. The systems were solvated using the OPC water model(Izadi et al., 2018) within a rectangular periodic box, with a minimum solute-to-box boundary distance of 12 Å. Parameters for Ca^2+^ interactions with relevant residues were calculated using the VFFDT program (.off and. frcmod are in the Supplementary Files).

The simulation protocol was standardized across all systems. Initial energy minimization was conducted to eliminate unfavorable interactions through four rounds totaling 2,500 steps. The first two rounds employed the steepest descent (SD) and conjugate gradient (CG) methods, respectively, with positional restraints applied to all atoms except water molecules and ions. The subsequent two rounds allowed the entire system to relax without restraints. Non-bonded interactions were truncated at a cutoff distance of 12 Å, and the SHAKE algorithm was used to constrain bonds involving hydrogen atoms. Following minimization, each system was heated from 0 K to 300 K over 200 ps under constant pressure (1 bar) using a Langevin thermostat, with protein atom positions restrained using a force constant of 10 kcal/(mol·Å^2^). A time step of 2 fs was applied during the heating phase. Subsequently, unrestrained conventional MD simulations were performed for 1.0 μs in the NPT ensemble (constant pressure and temperature).

Free energy calculations were conducted using the MMPBSA.py script in AmberTools 24 (Miller et al., 2012), employing the MM/GBSA implicit solvent model with a fixed ionic strength of 100 mmol/L. Additional analyses, including polar contact identification, hydrogen bond analysis, and correlation analyses, were performed using the cpptraj module (Roe and Cheatham, 2013).

### Cellular NO content experiments

In cell experiments, mouse neuronal cell lines were isolated and purchased from Sangon Bio. Neurons were cultured in CM-ZY003 complete neuronal medium (Pricella). Cells were passaged when reaching over 80% confluence. Initially, the cell culture supernatant was discarded, and cells were washed with 2 mL PBS followed by removal of the wash solution. Then, 700 μL of 0.25% trypsin was added, and the cells were placed in a CO₂ incubator for approximately 1.5 min. Under a microscope, cells were observed to become round, and the culture flask was gently tapped to detach cells. Subsequently, 2 mL of complete neuronal cell medium was added to dissociate all cells. The cell suspension was transferred to a 15 mL sterile centrifuge tube and centrifuged at 1000 rpm for 3 min at room temperature. The supernatant was discarded, and the cell pellet was resuspended in 1 mL of complete culture medium. Ten microliters of the cell suspension was mixed with 1 μL of trypan blue, and cell counting was performed using a hemocytometer. Based on cell counting, it was confirmed that cells were in the logarithmic growth phase. The cell suspension was evenly distributed in cell culture dishes. For experimental groups requiring addition of LPS and inhibitors, the final concentration of reagents were standardized to 145 μg/mL (for Spermidine), 1 μg/mL (for LPS) and 1 mM (for L-NMMA), respectively. All experimental and control groups were cultured for 12–24 h to ensure sufficient NO production. NO content was detected using a Nitric Oxide Content Assay Kit (Solarbio BC1475).

### Western blot analysis

#### Cell lysis and protein extraction

Suspension cells were harvested by centrifugation (1,000 rpm, 5 min) and lysed in ice-cold RIPA buffer (250 μL per 1 × 10^6^ cells) supplemented with 1% PMSF and protease inhibitor cocktail (freshly prepared before use). Adherent cells were washed 3 times with ice-cold PBS, followed by addition of RIPA buffer (250 μL per well for 6-well plates) and gentle agitation on ice for 5 min. Cells were scraped and transferred to microcentrifuge tubes, incubated on ice for 30 min with intermittent vortexing, and centrifuged at 12,000 rpm for 15 min at 4 °C. Supernatants were collected, and protein concentrations were determined using a BCA Protein Assay Kit.

#### SDS-PAGE and transfer

Equal amounts of protein (20–30 μg) were mixed with 5 × reducing loading buffer, denatured at 100 °C for 10 min, and separated by SDS-PAGE on 10–15% polyacrylamide gels (depending on target protein molecular weight). Proteins were transferred to PVDF membranes (0.22 μm) using a wet transfer system at 200 mA for 90 min at 4 °C. Membranes were briefly stained with Ponceau S to verify transfer efficiency and blocked with 5% non-fat milk in TBST (20 mM Tris–HCl, 150 mM NaCl, 0.1% Tween-2, pH 7.4) for 1 h at room temperature.

#### Immunoblotting

Membranes were incubated overnight at 4 °C with primary antibodies diluted in 5% BSA/TBST (antibody concentrations optimized according to manufacturer’s instructions). After 3 washes with TBST (10 min each), membranes were incubated with HRP-conjugated secondary antibodies (1:5,000 dilution in TBST) for 1 h at room temperature. Following 3 additional TBST washes, signals were detected using an enhanced chemiluminescence (ECL) kit. Images were captured using a digital imaging system, and band intensities were quantified using ImageJ software. *β*-actin or GAPDH was used as an internal loading control.

#### Key parameters

Primary Antibodies: Diluted in 5% BSA/TBST (e.g., anti-target protein 1:1,000; anti-β-actin 1:5,000).

Electrophoresis Conditions: Stacking gel at 75 V, resolving gel at 100 V.

Transfer Efficiency: Verified using pre-stained molecular weight markers.

Signal Detection: ECL exposure times adjusted based on target protein abundance (10 s–5 min).

All experiments were repeated independently at least three times. Data were presented as mean ± standard deviation (SD) and analyzed using Student’s t-test or one-way ANOVA.

## Data Availability

The original contributions presented in the study are included in the article/Supplementary material, further inquiries can be directed to the corresponding author.
