# Supplementary material for: FNR-like unit interacts with C-terminal related residues trigger nNOS reductase domain conformational flexibility change
Source: Front Neurosci. 2026 Feb 11;20:1751011. doi: 10.3389/fnins.2026.1751011 (PMC12932508; doi:10.3389/fnins.2026.1751011)
Supplement: Supplementary file 1 [file Data_Sheet_1.zip › Supplementary Figure S1.docx]

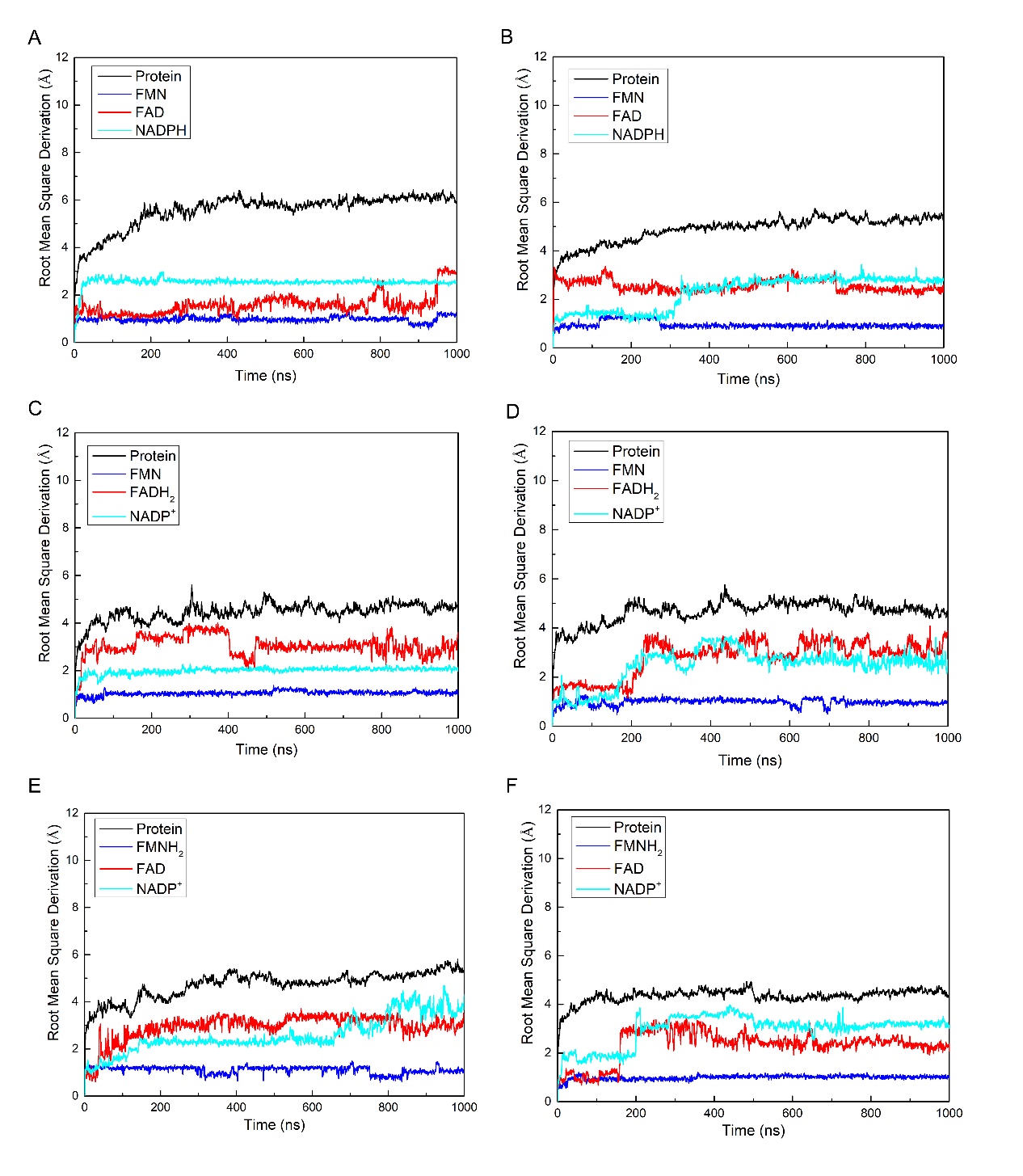


Figure S1. Independent MD runs of the nNOS reductase domain across different redox states. (A–B) Two independent runs of root-mean-square deviation (RMSD) plots as a function of simulation time for the protein backbone (black), FMN (violet), FAD (magenta), and NADP(H)/NADP⁺ (cyan) in Model 1, respectively. (C–D) Two independent runs of root-mean-square deviation (RMSD) plots as a function of simulation time for the protein backbone (black), FMN (violet), FAD (magenta), and NADP(H)/NADP⁺ (cyan) in Model 2, respectively. (E–F) Two independent runs of root-mean-square deviation (RMSD) plots as a function of simulation time for the protein backbone (black), FMN (violet), FAD (magenta), and NADP(H)/NADP⁺ (cyan) in Model 3, respectively. Raw-data and terminal conformations are stored at “Model_repeat” folder in the supplementary file “raw_data.zip”.
