# Supplementary figures and images for: FNR-like unit interacts with C-terminal related residues trigger nNOS reductase domain conformational flexibility change
Source: Front Neurosci. 2026 Feb 11;20:1751011. doi: 10.3389/fnins.2026.1751011 (PMC12932508; doi:10.3389/fnins.2026.1751011)

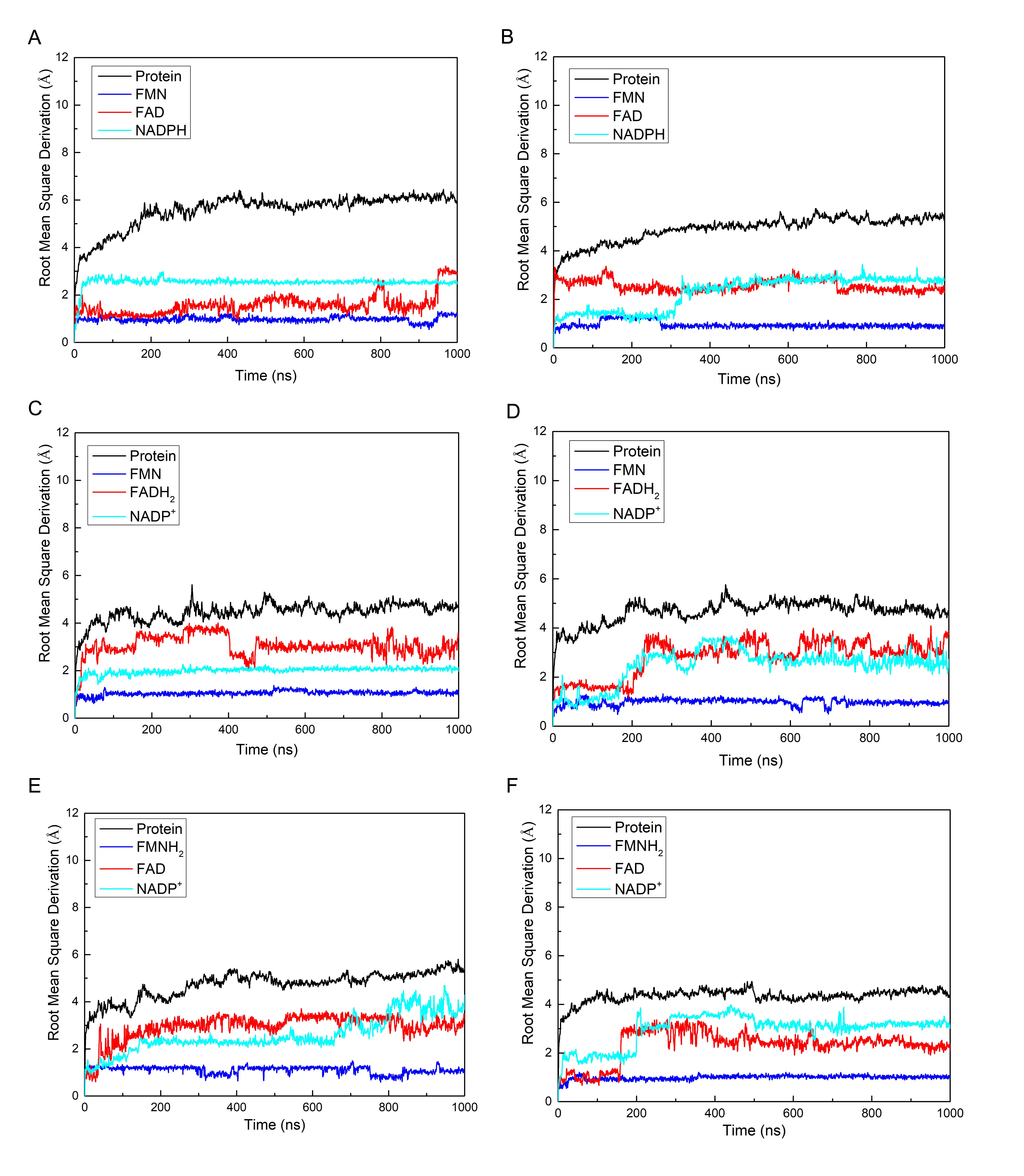

Supplement: Supplementary file 1 [file Data_Sheet_1.zip › FigureS1.jpg]

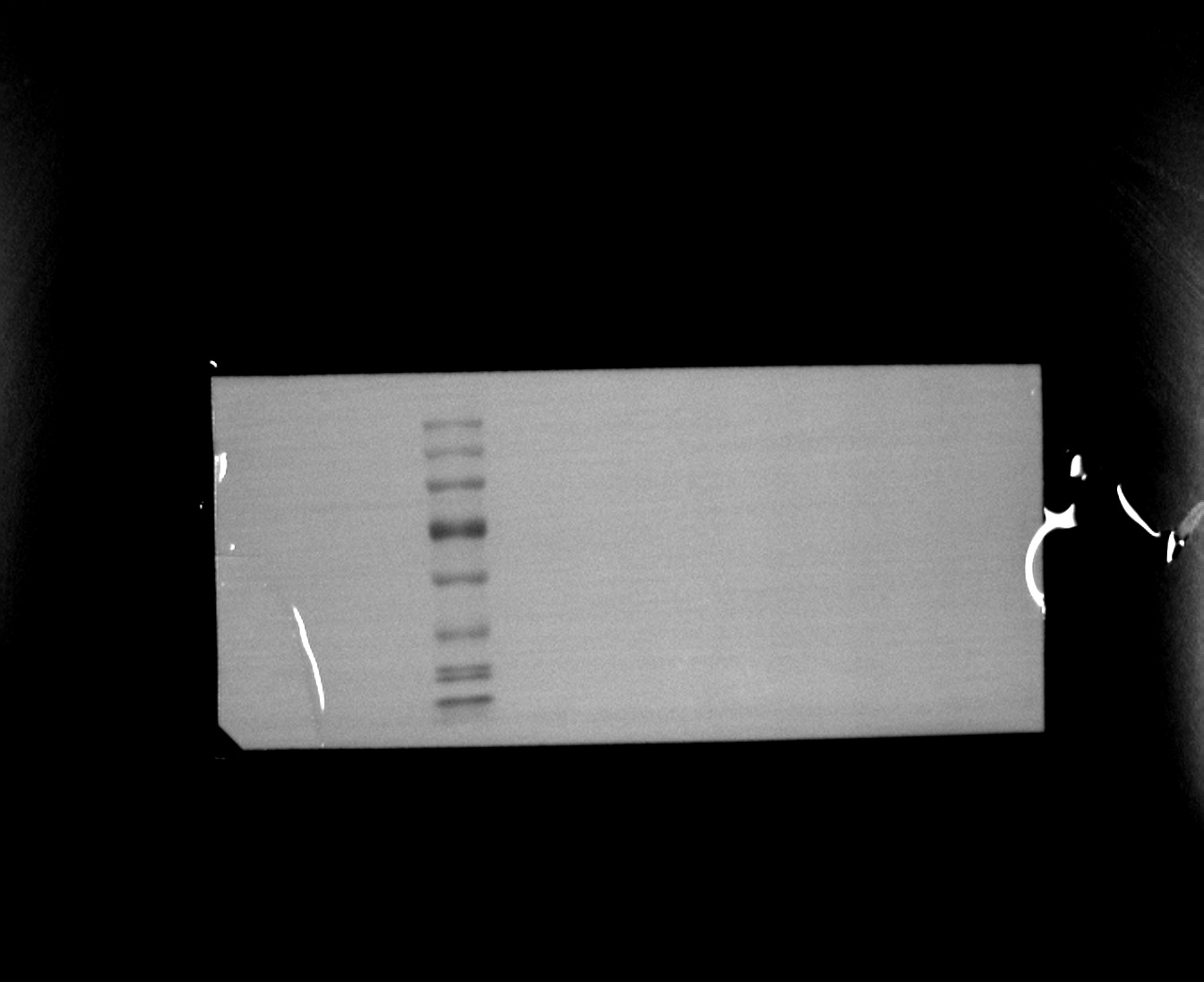

Supplement: Supplementary file 4 [file Data_Sheet_4.zip › uncropped-blot/1/NLRP3 1-1.Tif]

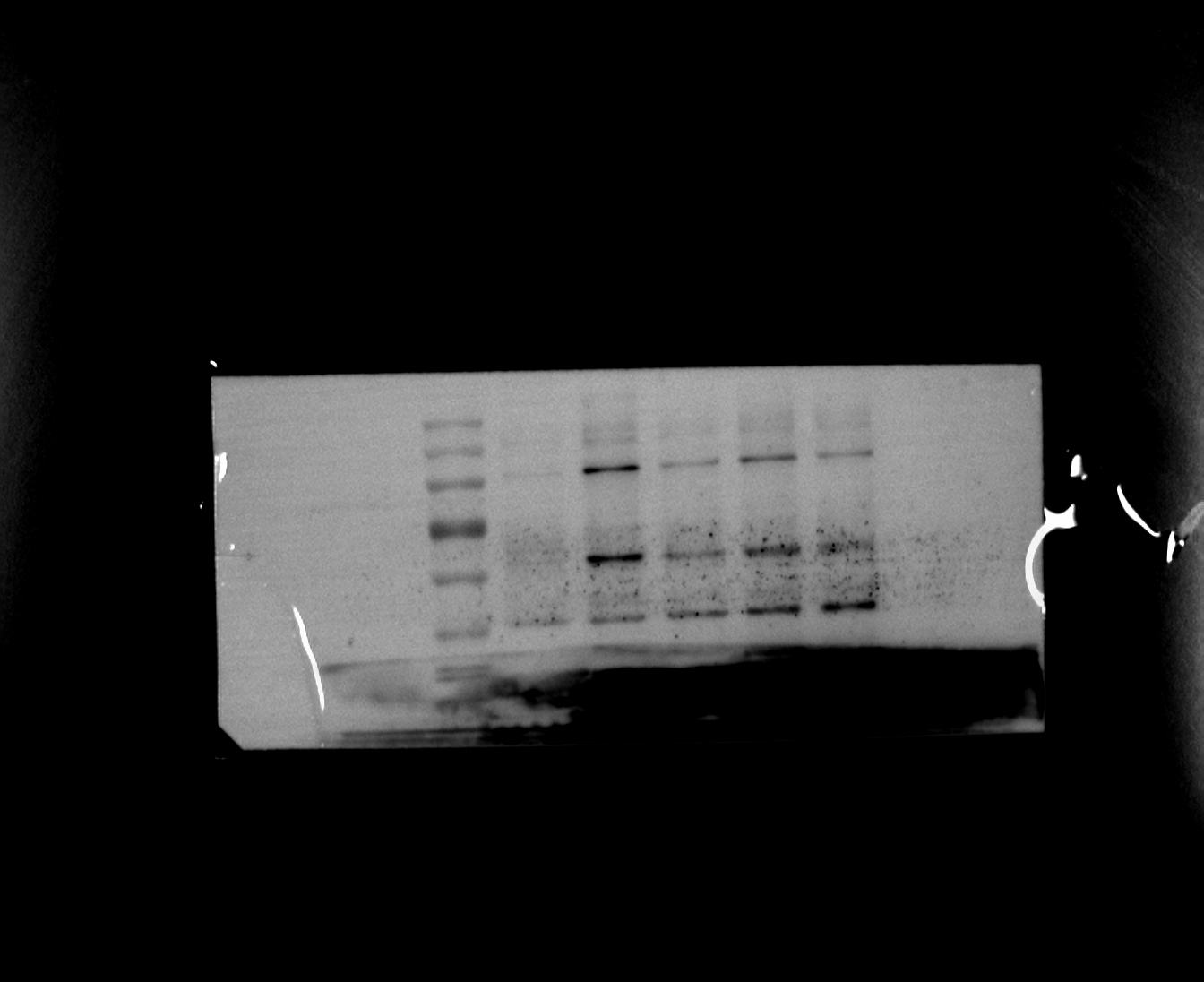

Supplement: Supplementary file 4 [file Data_Sheet_4.zip › uncropped-blot/1/NLRP3 1-2.tif]

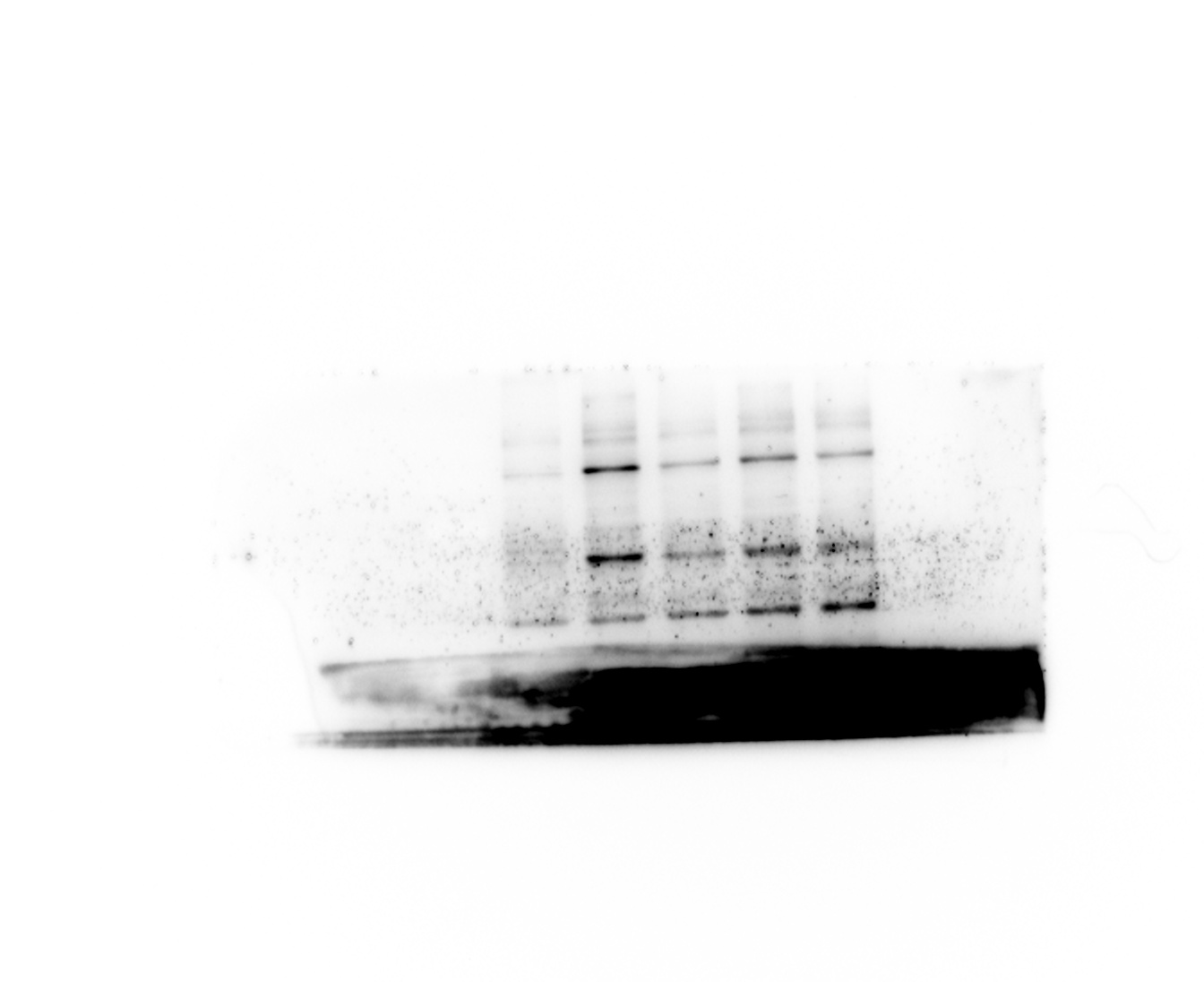

Supplement: Supplementary file 4 [file Data_Sheet_4.zip › uncropped-blot/1/NLRP3 1.Tif]

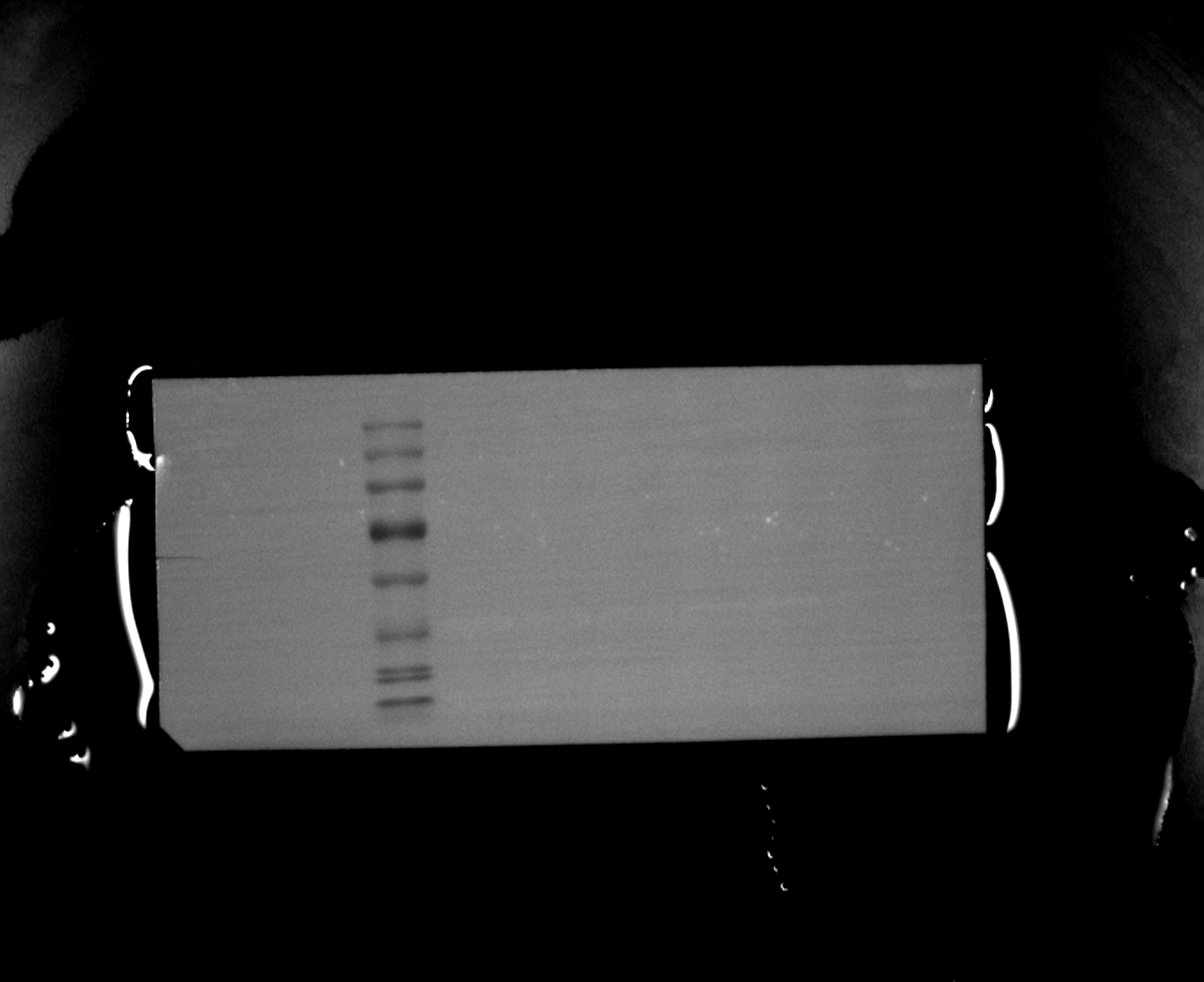

Supplement: Supplementary file 4 [file Data_Sheet_4.zip › uncropped-blot/1/NLRP3 β-actin 1-1.Tif]

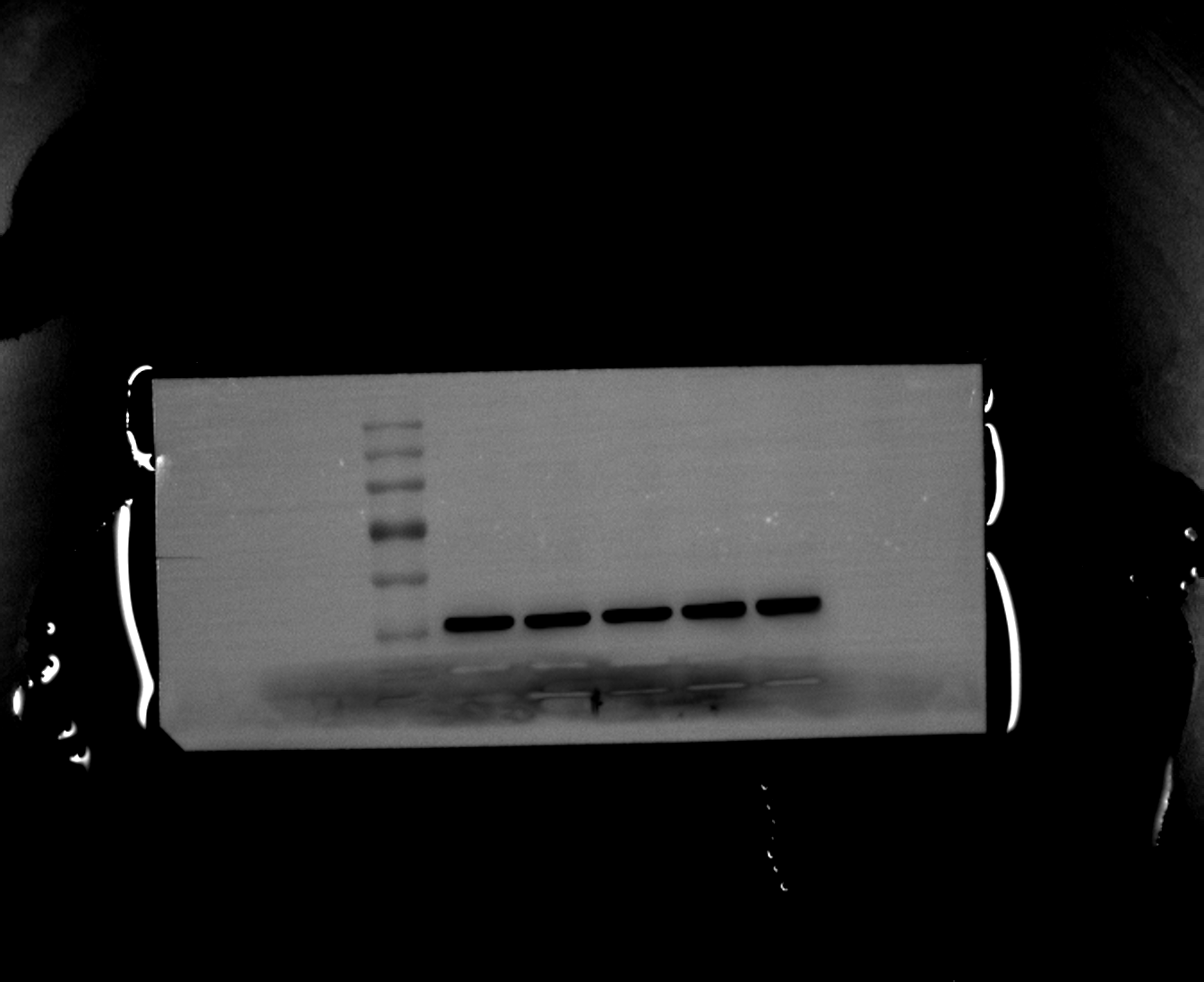

Supplement: Supplementary file 4 [file Data_Sheet_4.zip › uncropped-blot/1/NLRP3 β-actin 1-2.tif]

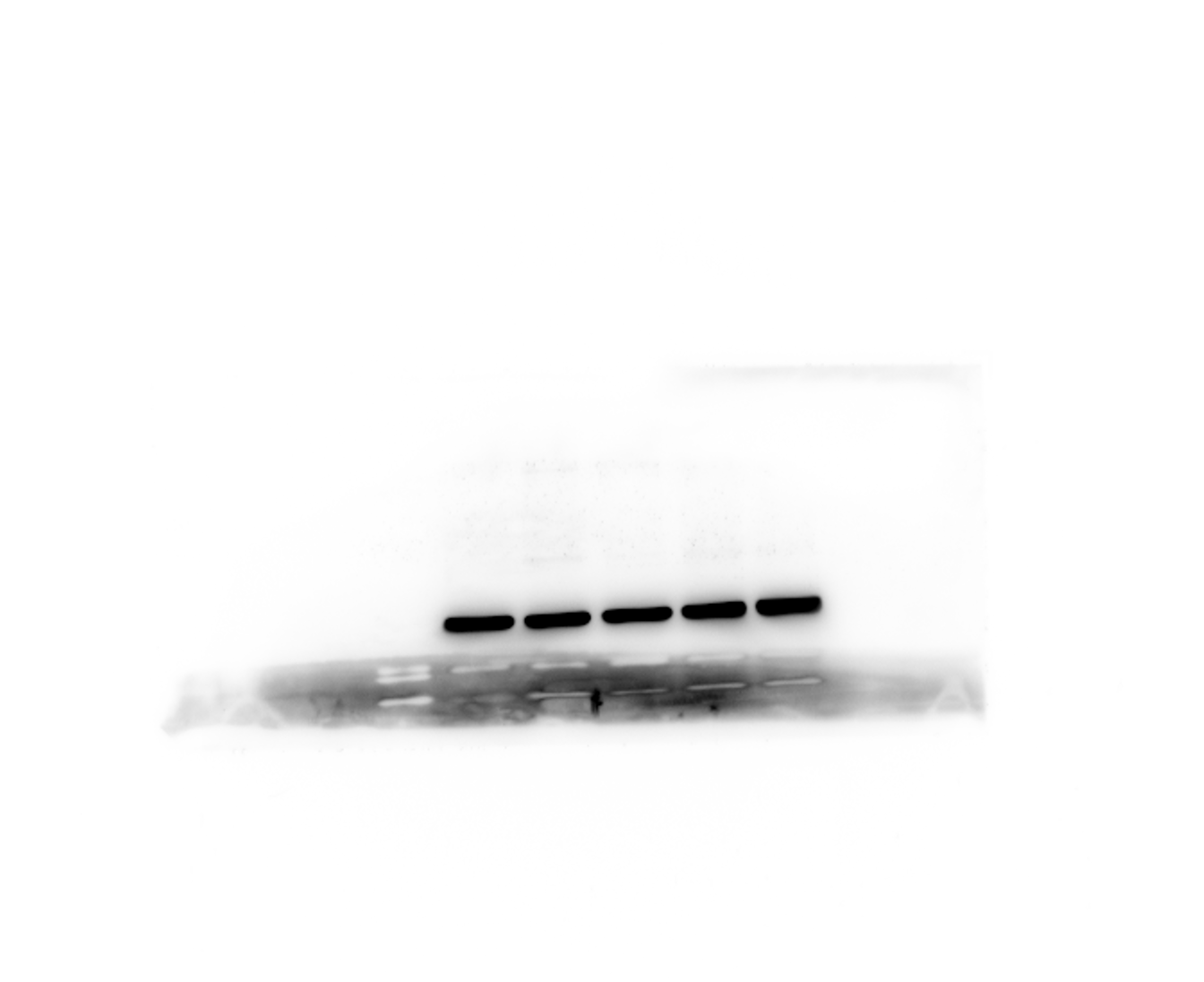

Supplement: Supplementary file 4 [file Data_Sheet_4.zip › uncropped-blot/1/NLRP3 β-actin 1.Tif]

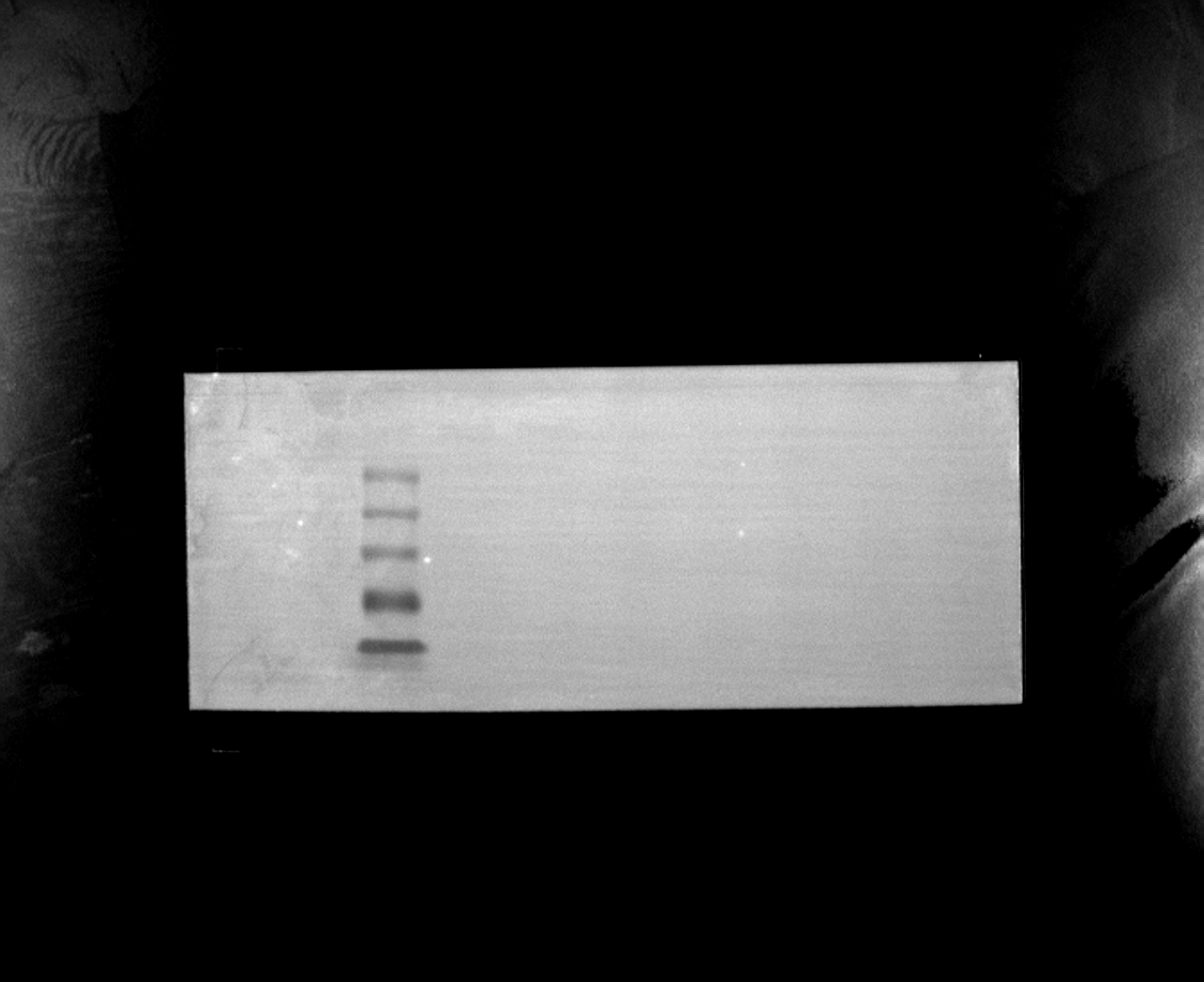

Supplement: Supplementary file 4 [file Data_Sheet_4.zip › uncropped-blot/1/NOS1 1-1.Tif]

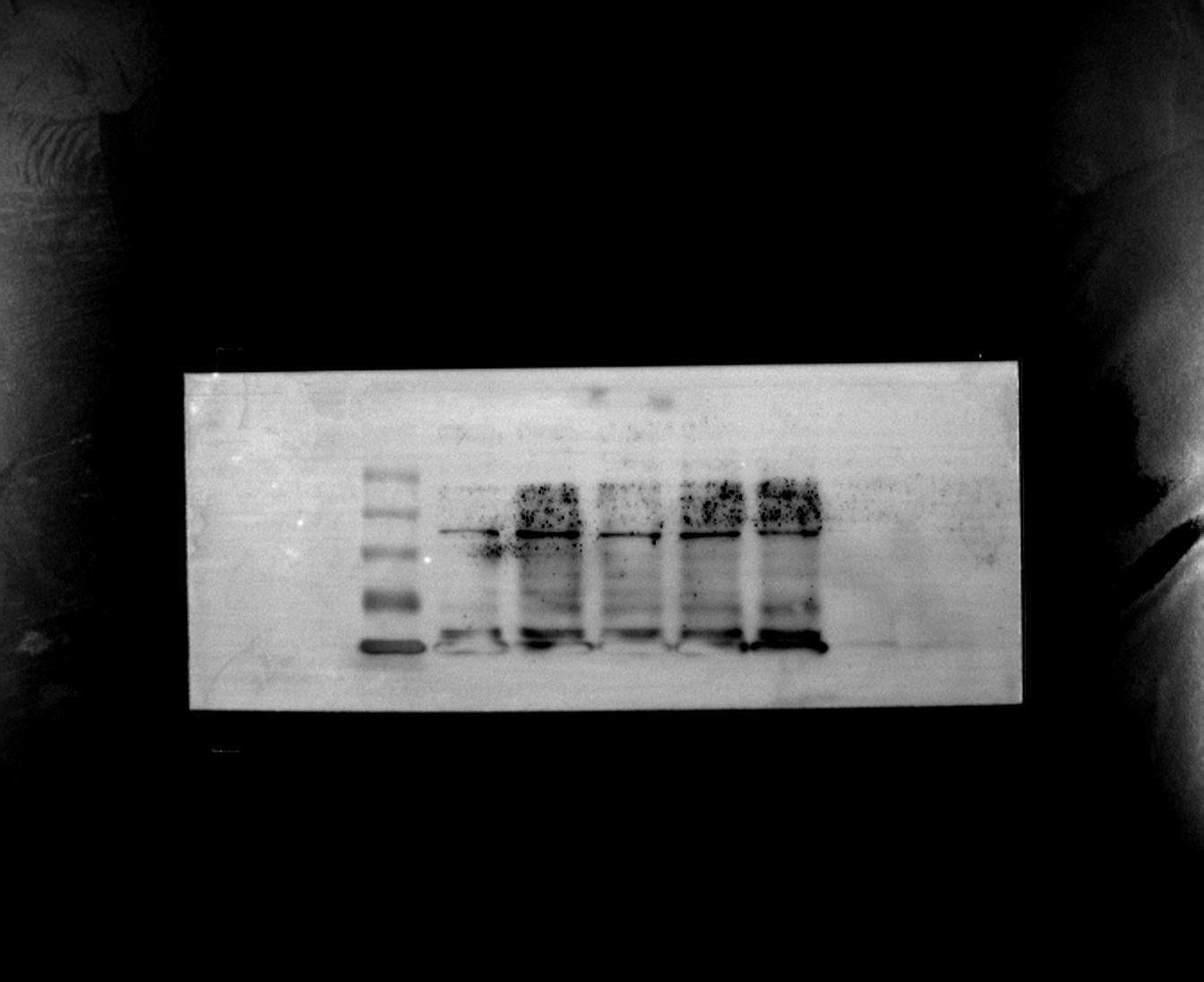

Supplement: Supplementary file 4 [file Data_Sheet_4.zip › uncropped-blot/1/NOS1 1-2.tif]

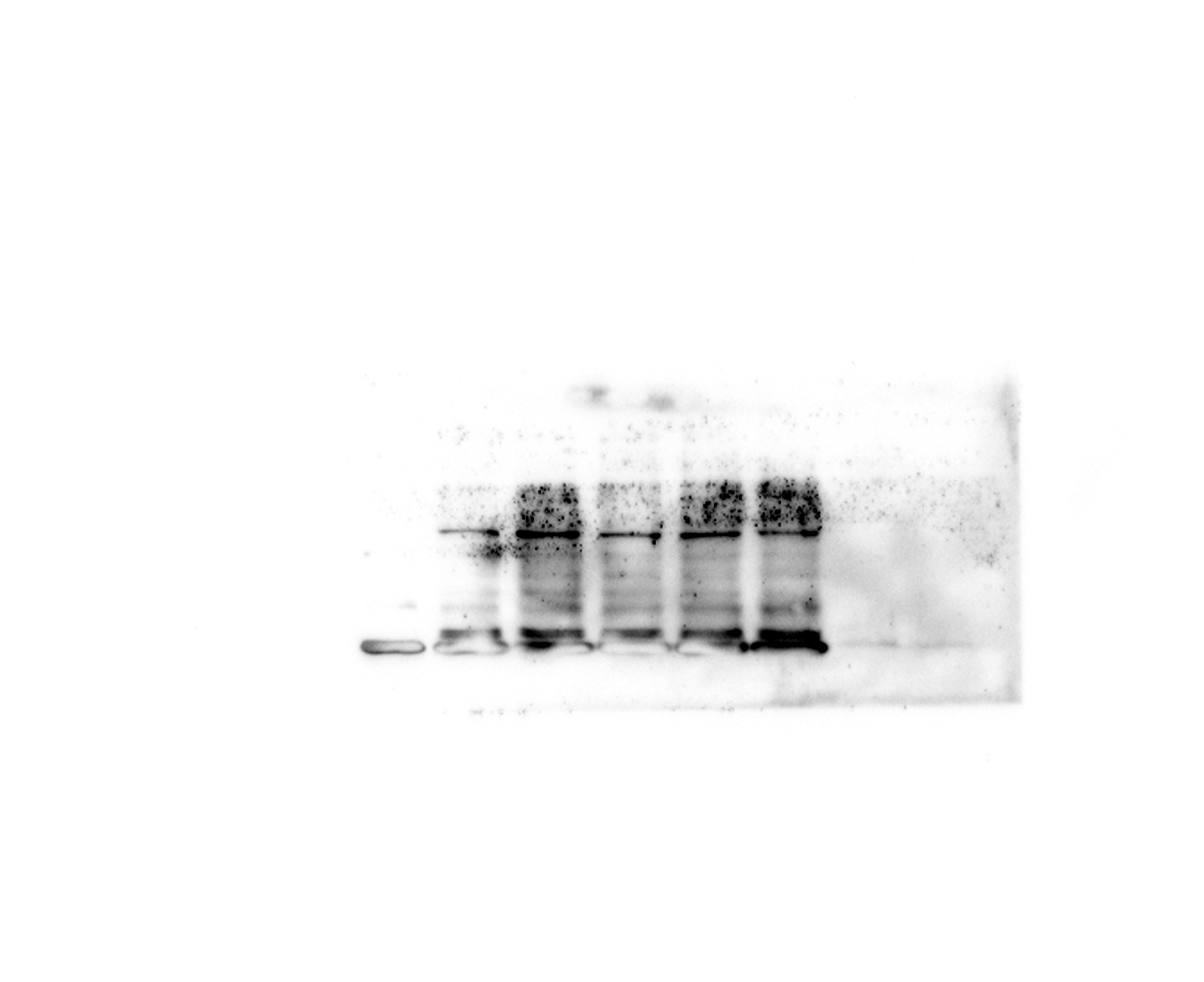

Supplement: Supplementary file 4 [file Data_Sheet_4.zip › uncropped-blot/1/NOS1 1.Tif]

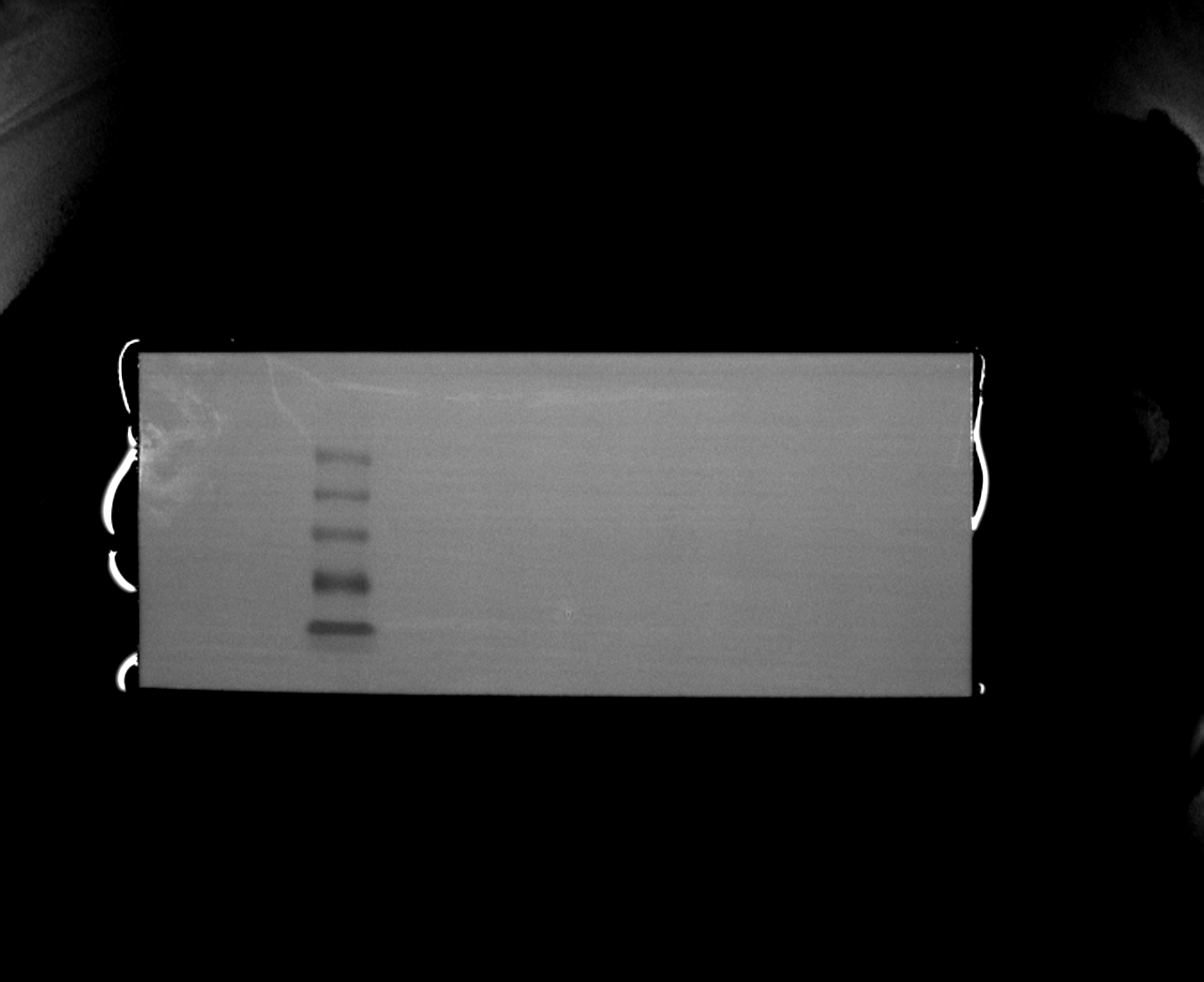

Supplement: Supplementary file 4 [file Data_Sheet_4.zip › uncropped-blot/1/NOS1 β-actin 1-1.Tif]

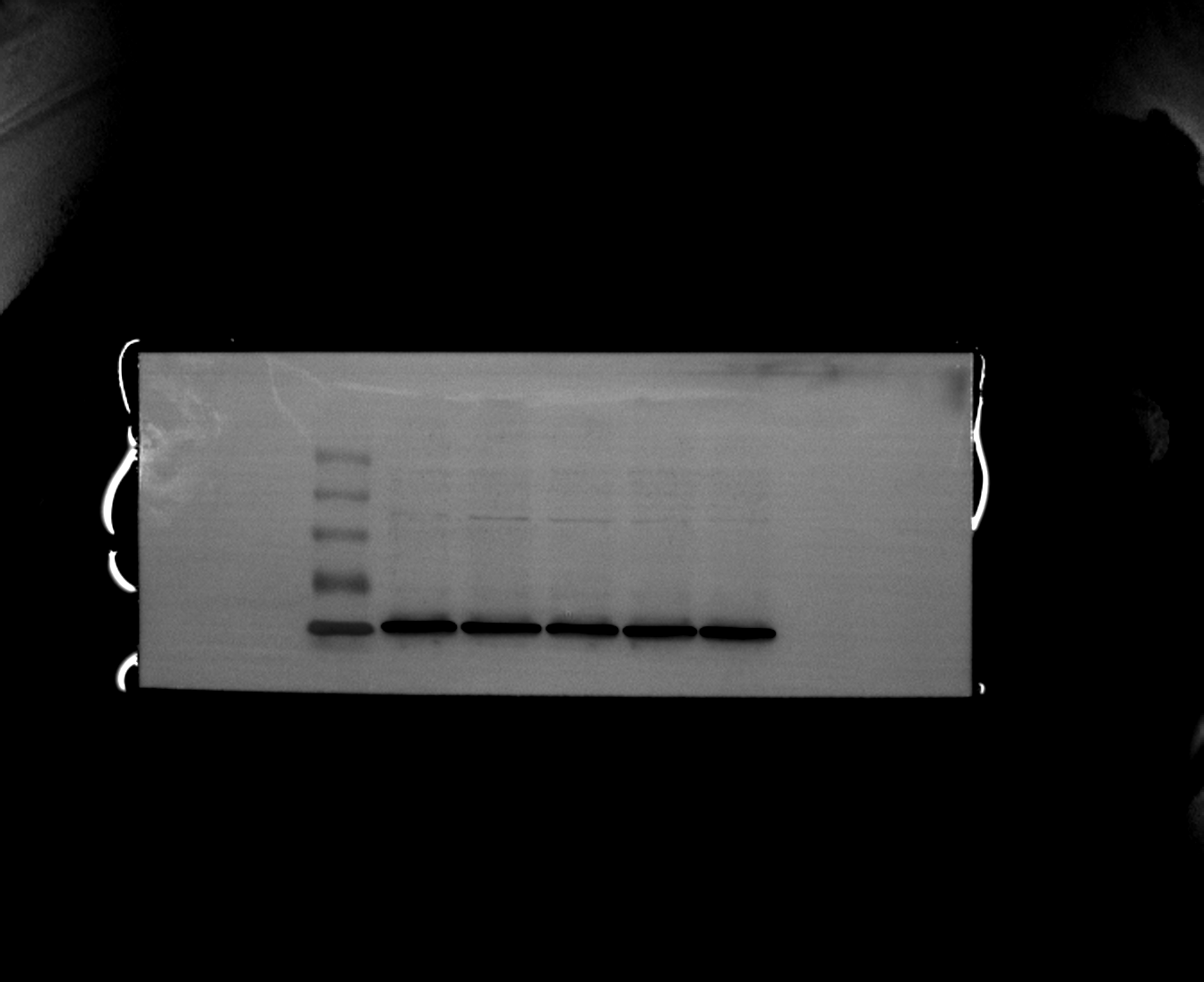

Supplement: Supplementary file 4 [file Data_Sheet_4.zip › uncropped-blot/1/NOS1 β-actin 1-2.tif]

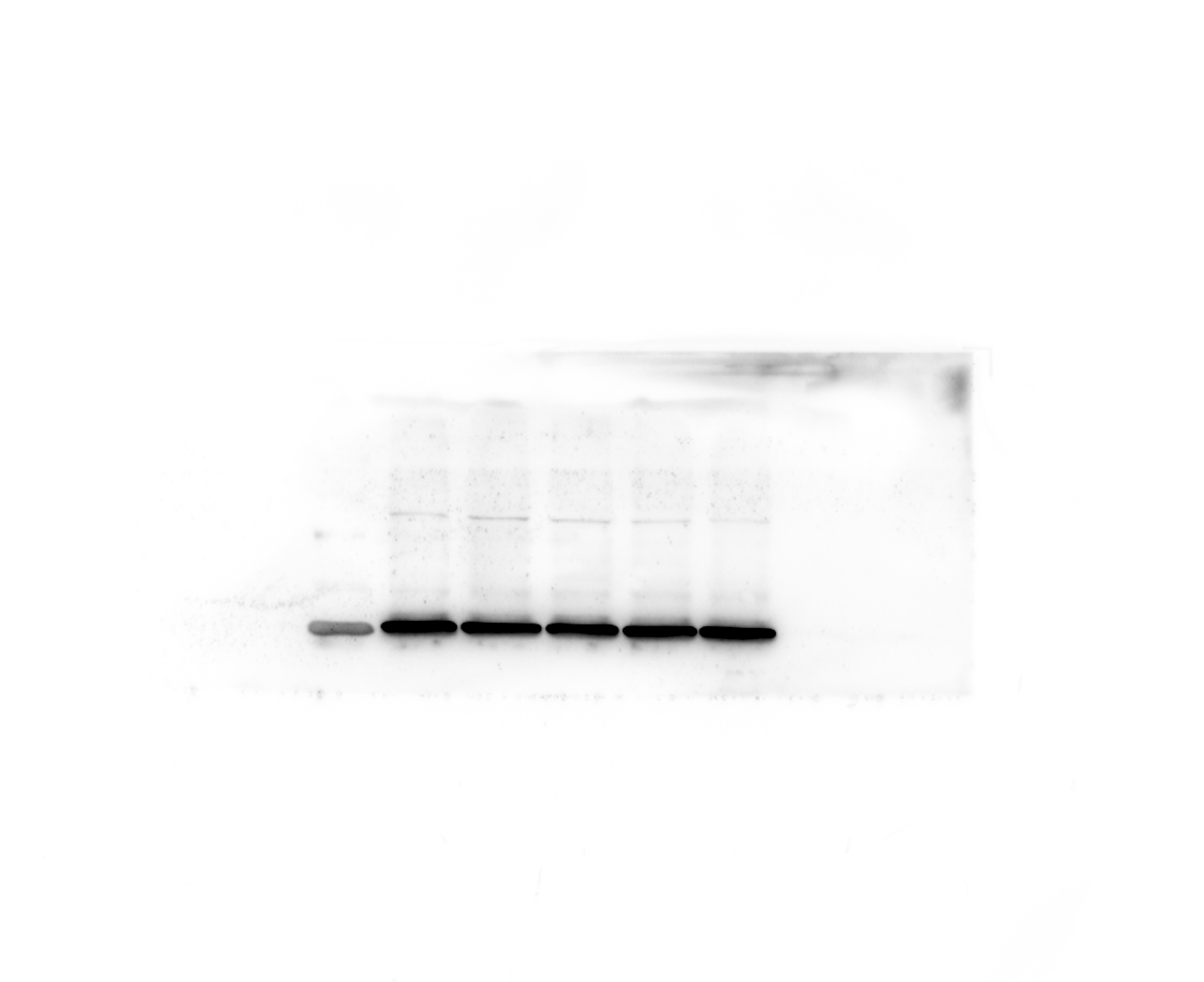

Supplement: Supplementary file 4 [file Data_Sheet_4.zip › uncropped-blot/1/NOS1 β-actin 1.Tif]

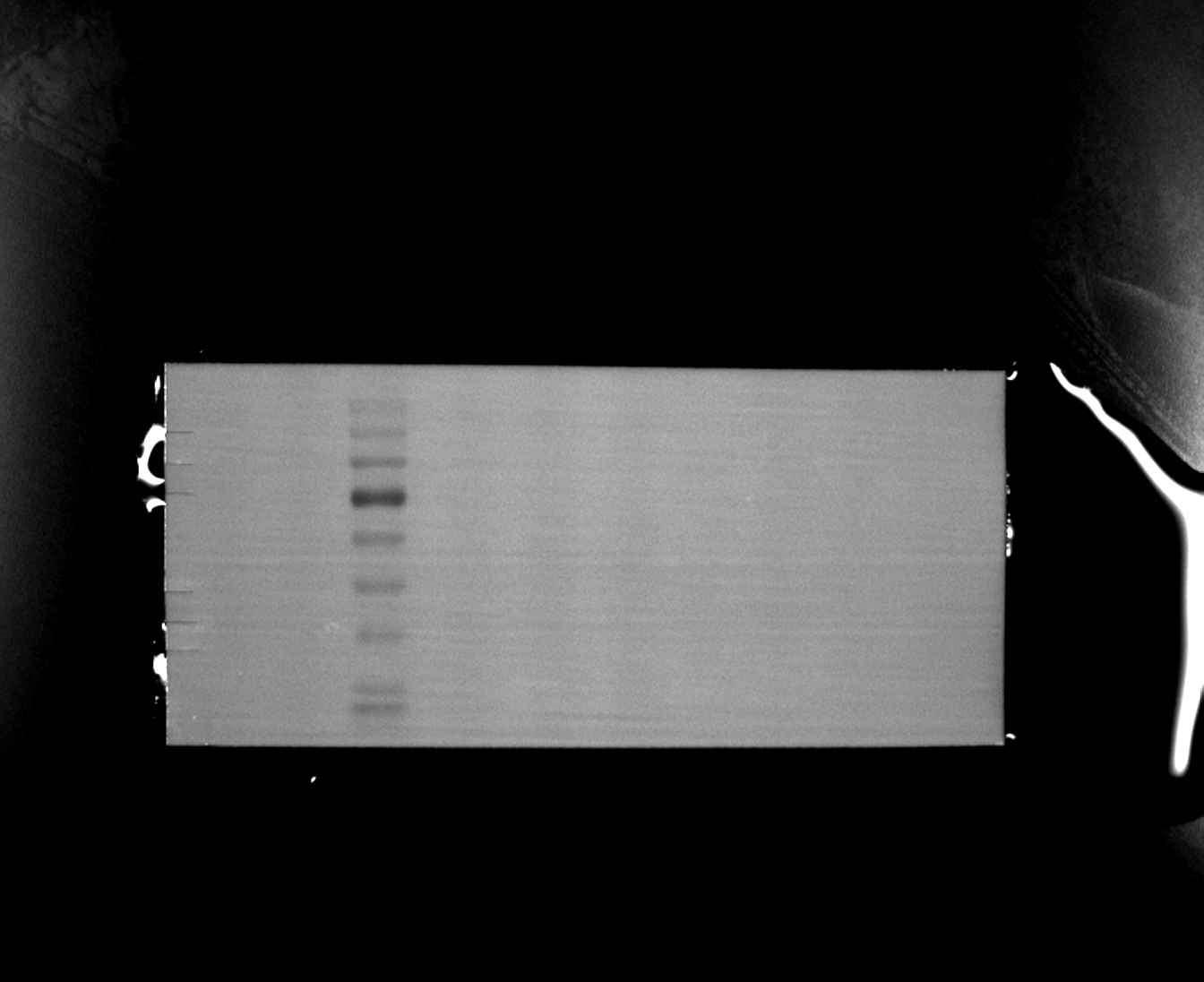

Supplement: Supplementary file 4 [file Data_Sheet_4.zip › uncropped-blot/2/NLRP3 2-1.Tif]

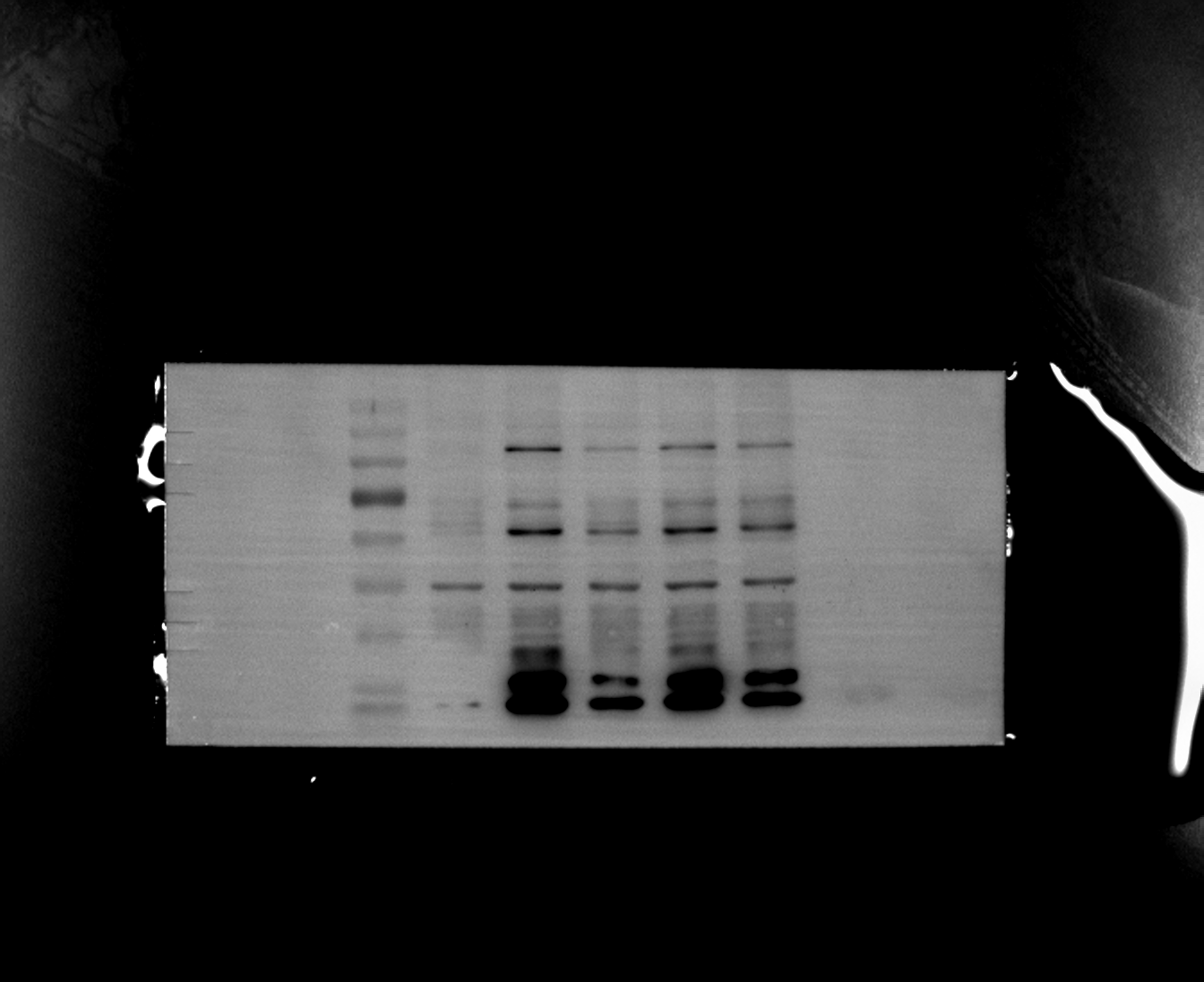

Supplement: Supplementary file 4 [file Data_Sheet_4.zip › uncropped-blot/2/NLRP3 2-2.tif]

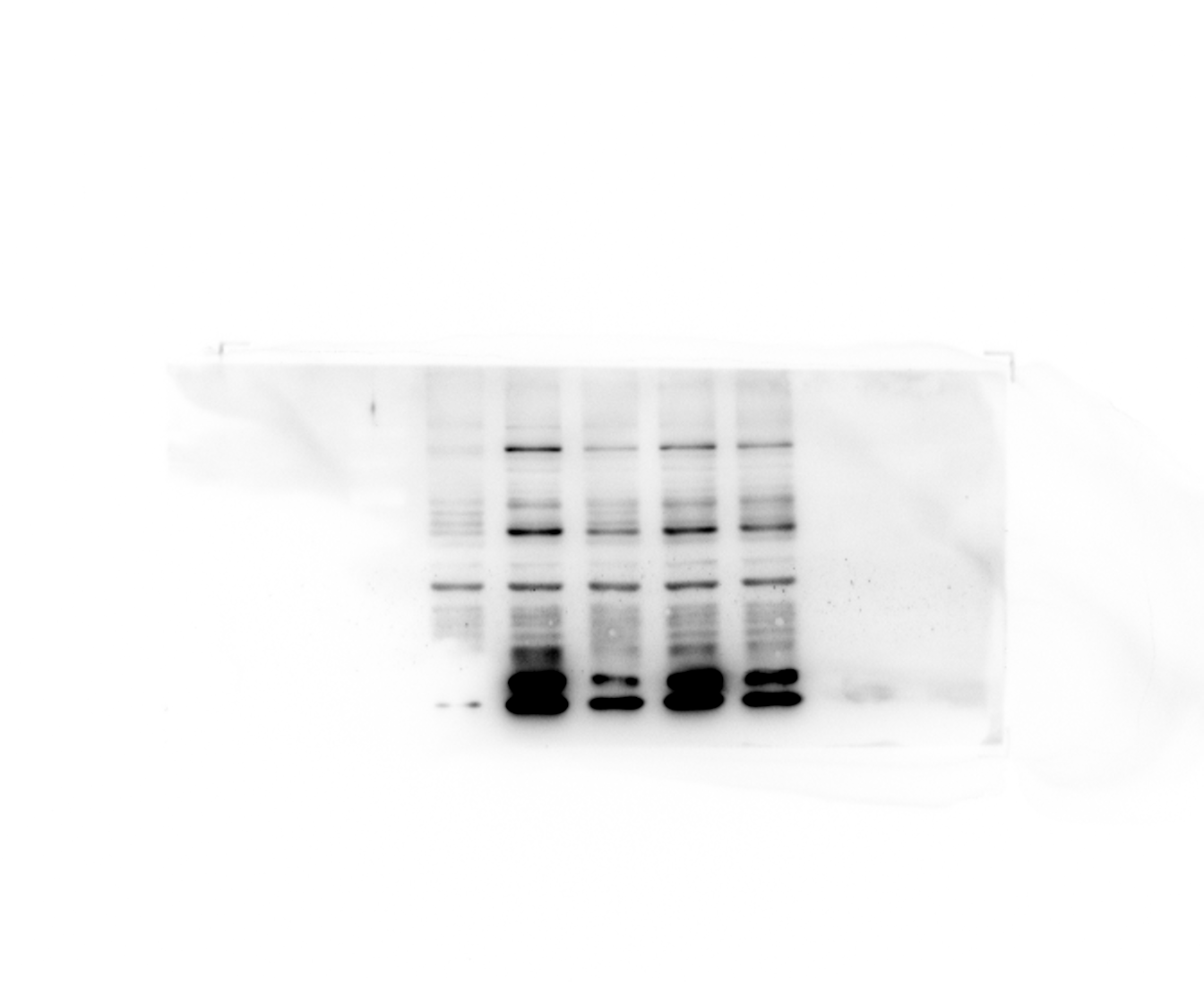

Supplement: Supplementary file 4 [file Data_Sheet_4.zip › uncropped-blot/2/NLRP3 2.Tif]

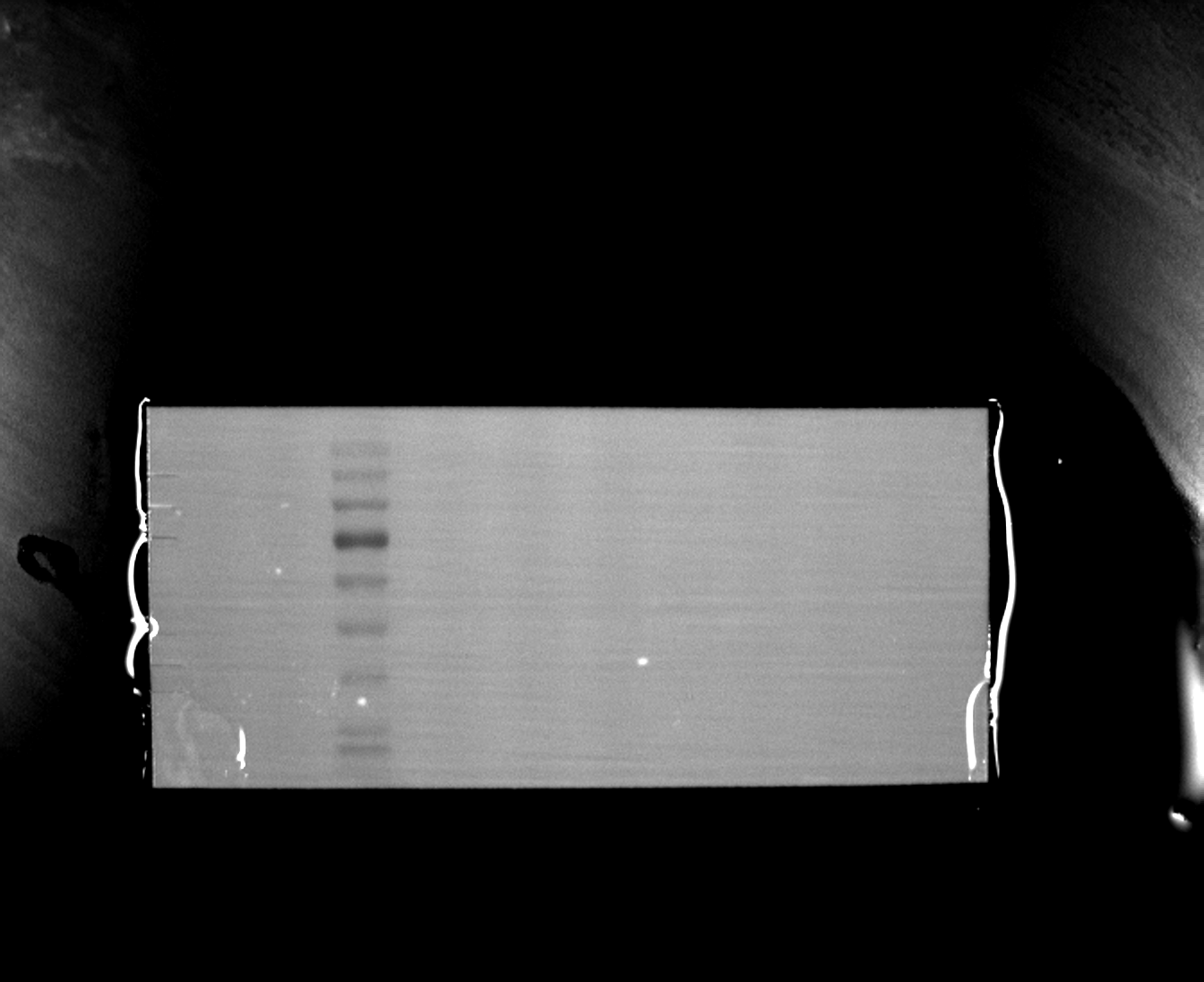

Supplement: Supplementary file 4 [file Data_Sheet_4.zip › uncropped-blot/2/NLRP3 β-actin 2-1.Tif]

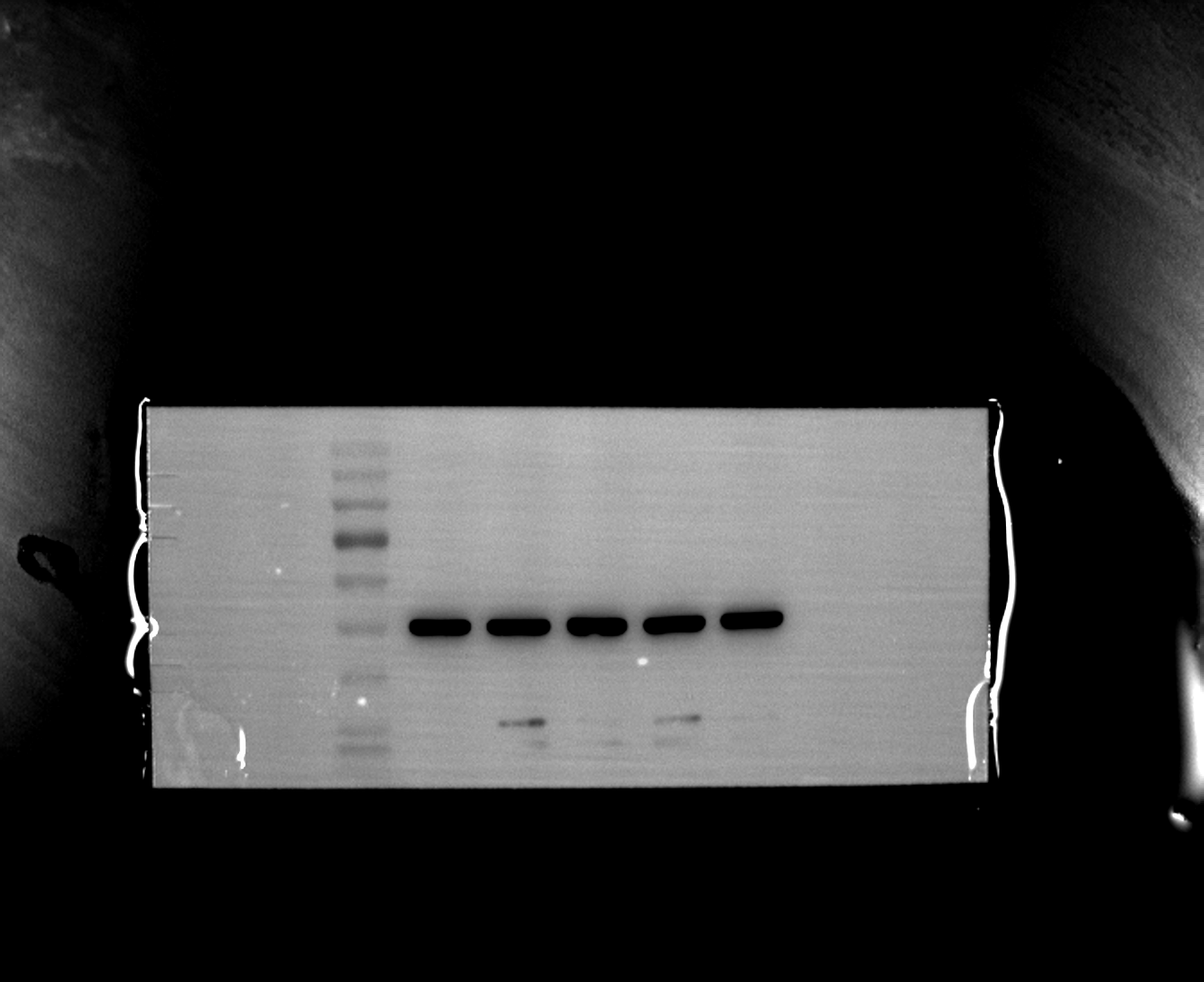

Supplement: Supplementary file 4 [file Data_Sheet_4.zip › uncropped-blot/2/NLRP3 β-actin 2-2.tif]

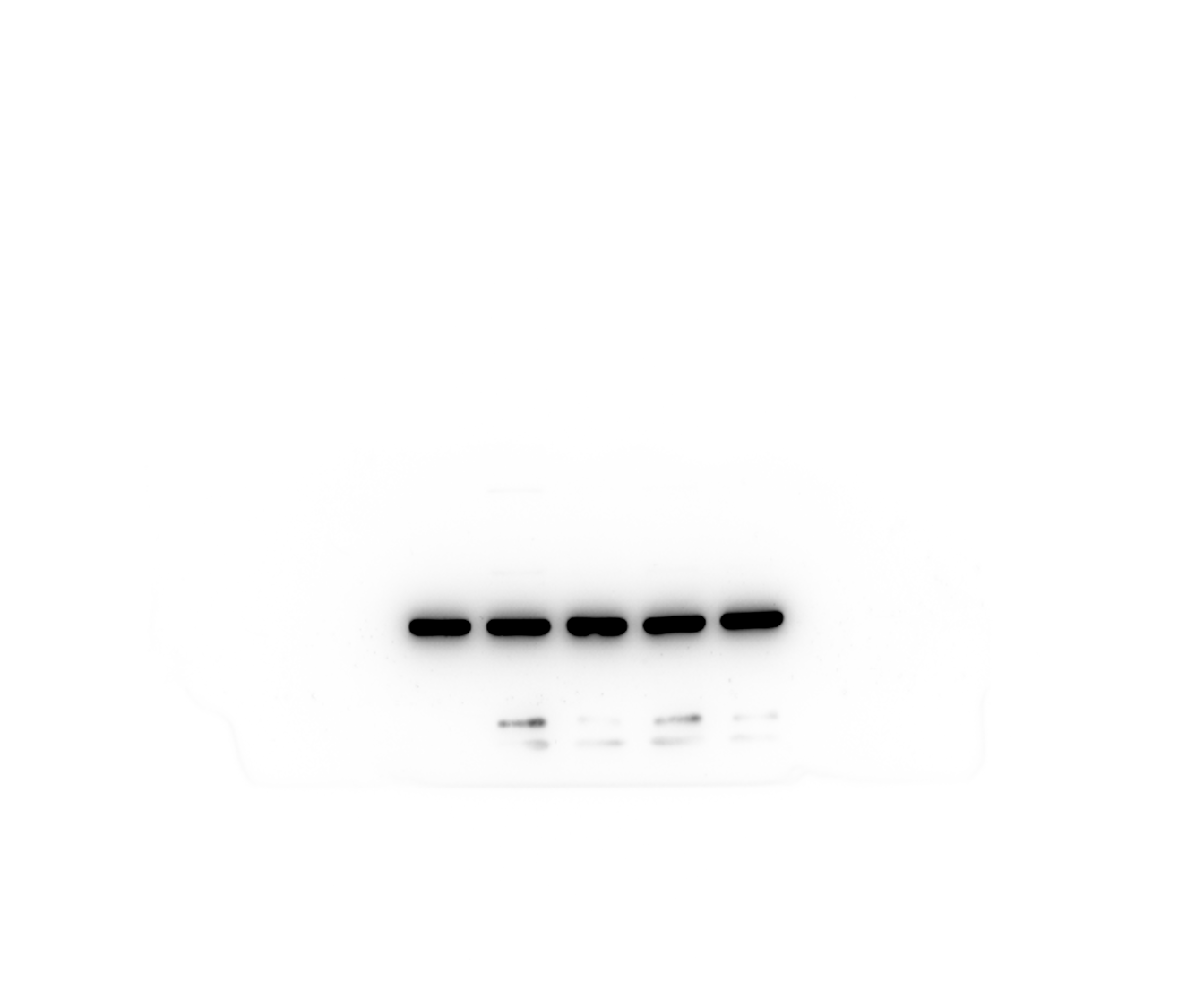

Supplement: Supplementary file 4 [file Data_Sheet_4.zip › uncropped-blot/2/NLRP3 β-actin 2.Tif]

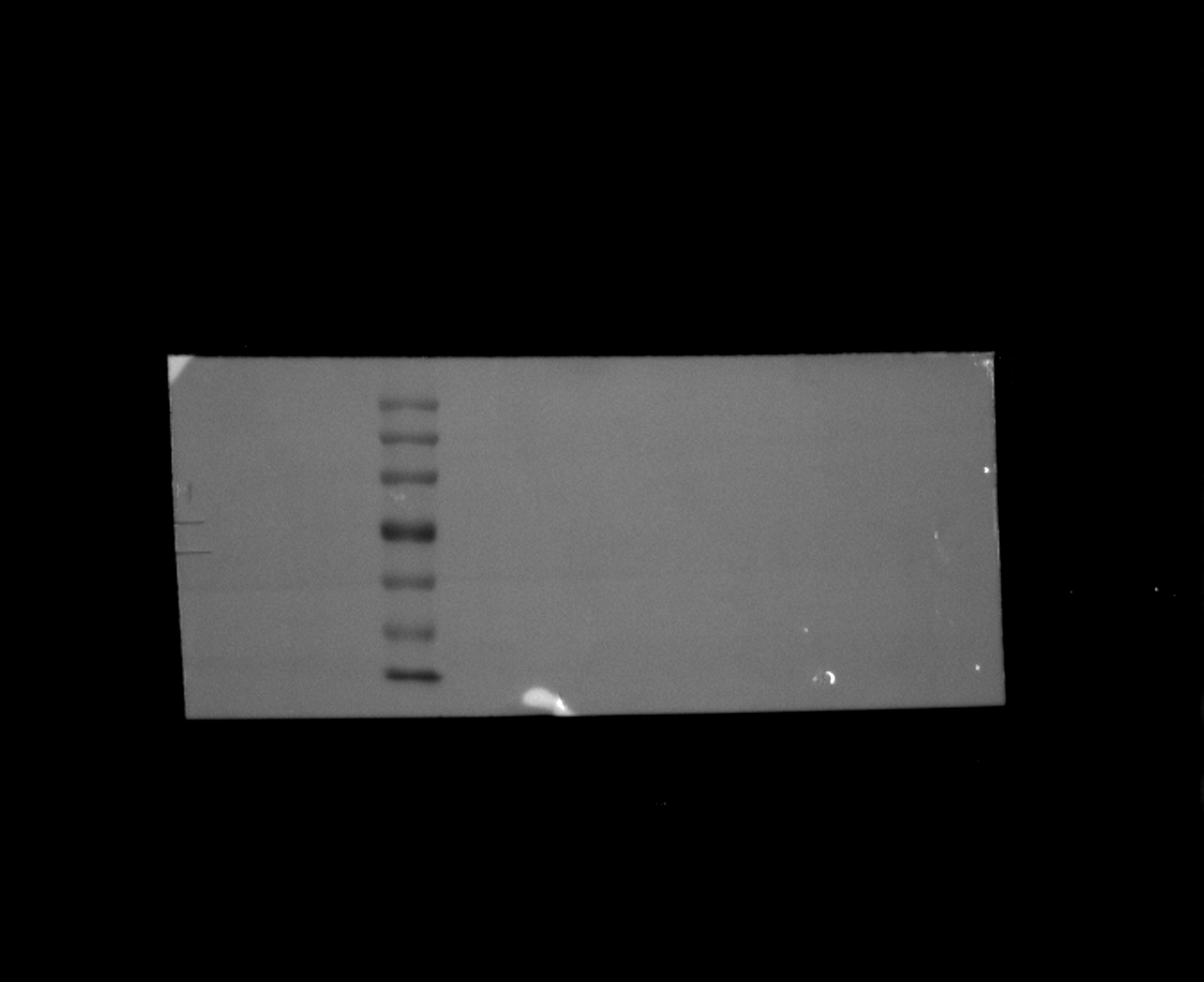

Supplement: Supplementary file 4 [file Data_Sheet_4.zip › uncropped-blot/2/NOS1 2-1.Tif]

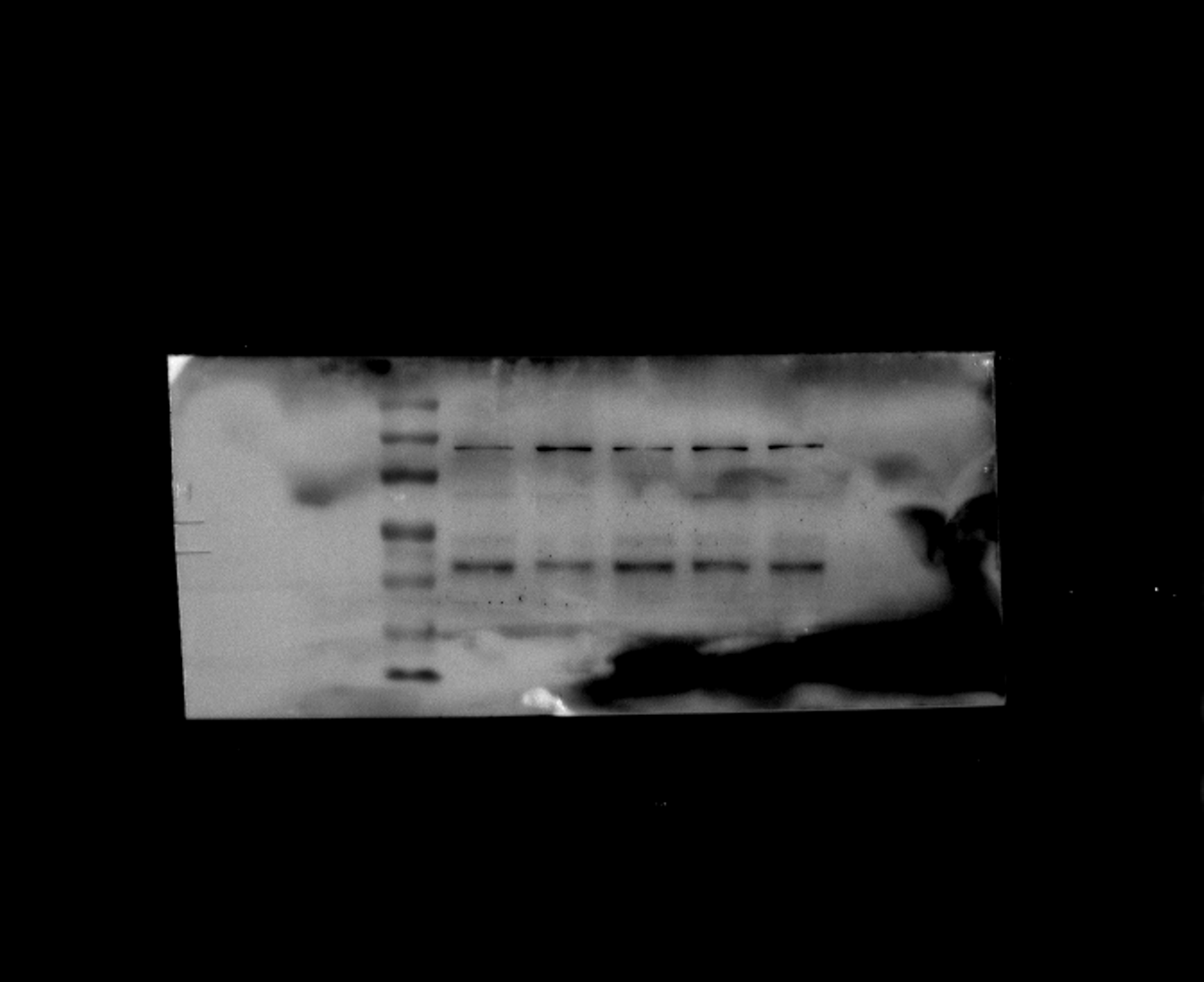

Supplement: Supplementary file 4 [file Data_Sheet_4.zip › uncropped-blot/2/NOS1 2-2.Tif]

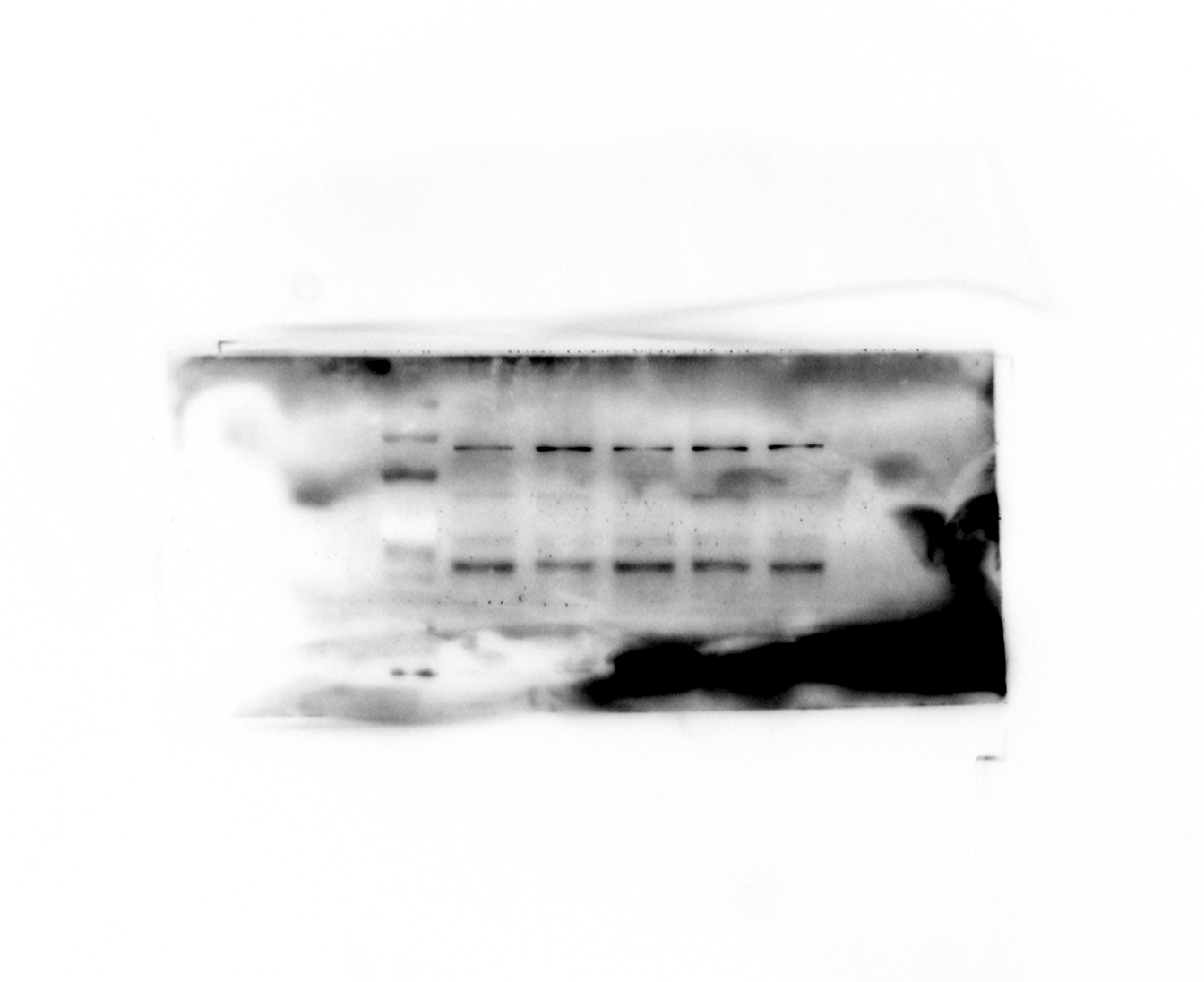

Supplement: Supplementary file 4 [file Data_Sheet_4.zip › uncropped-blot/2/NOS1 2.Tif]

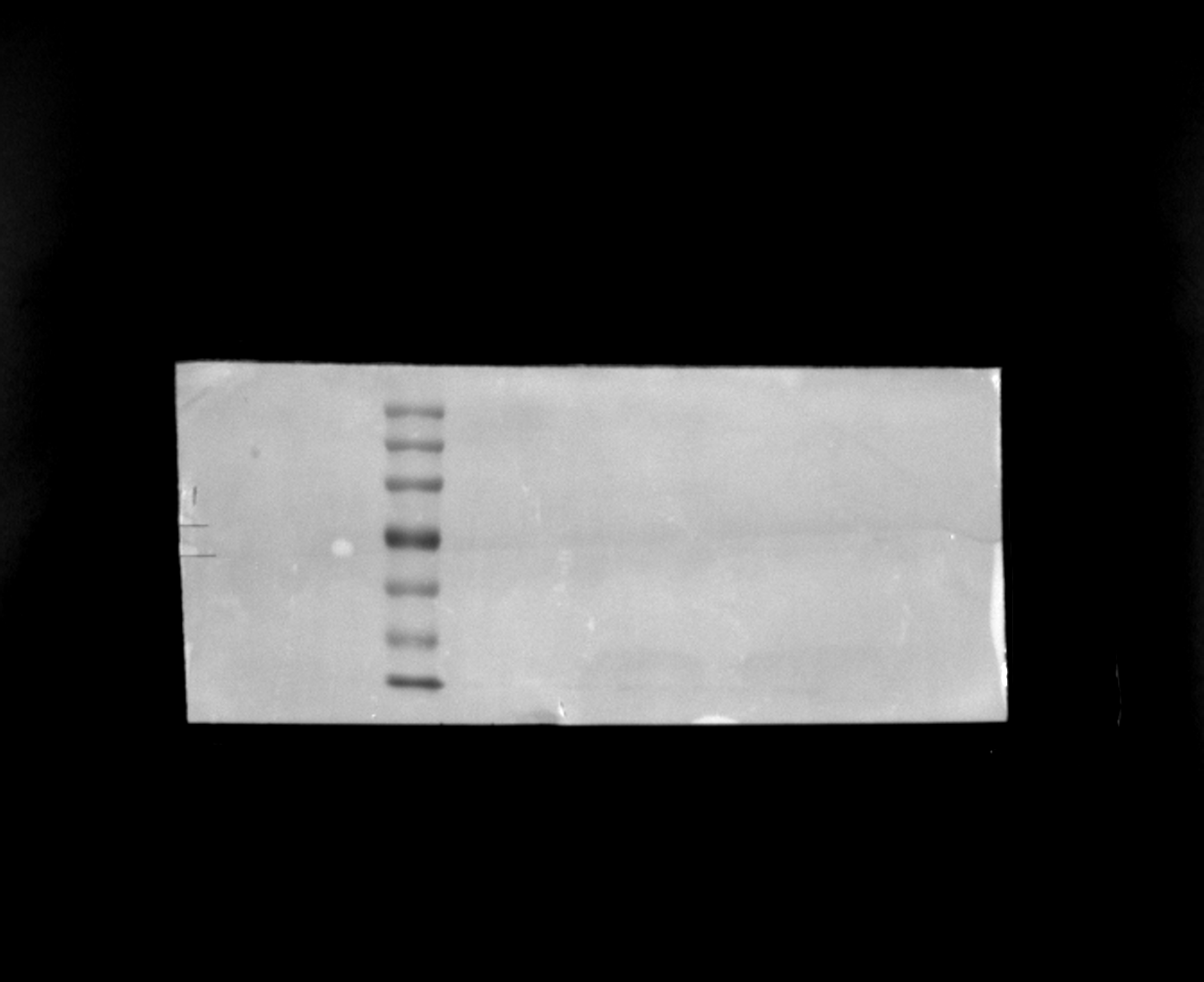

Supplement: Supplementary file 4 [file Data_Sheet_4.zip › uncropped-blot/2/NOS1 β-actin 2-1.Tif]

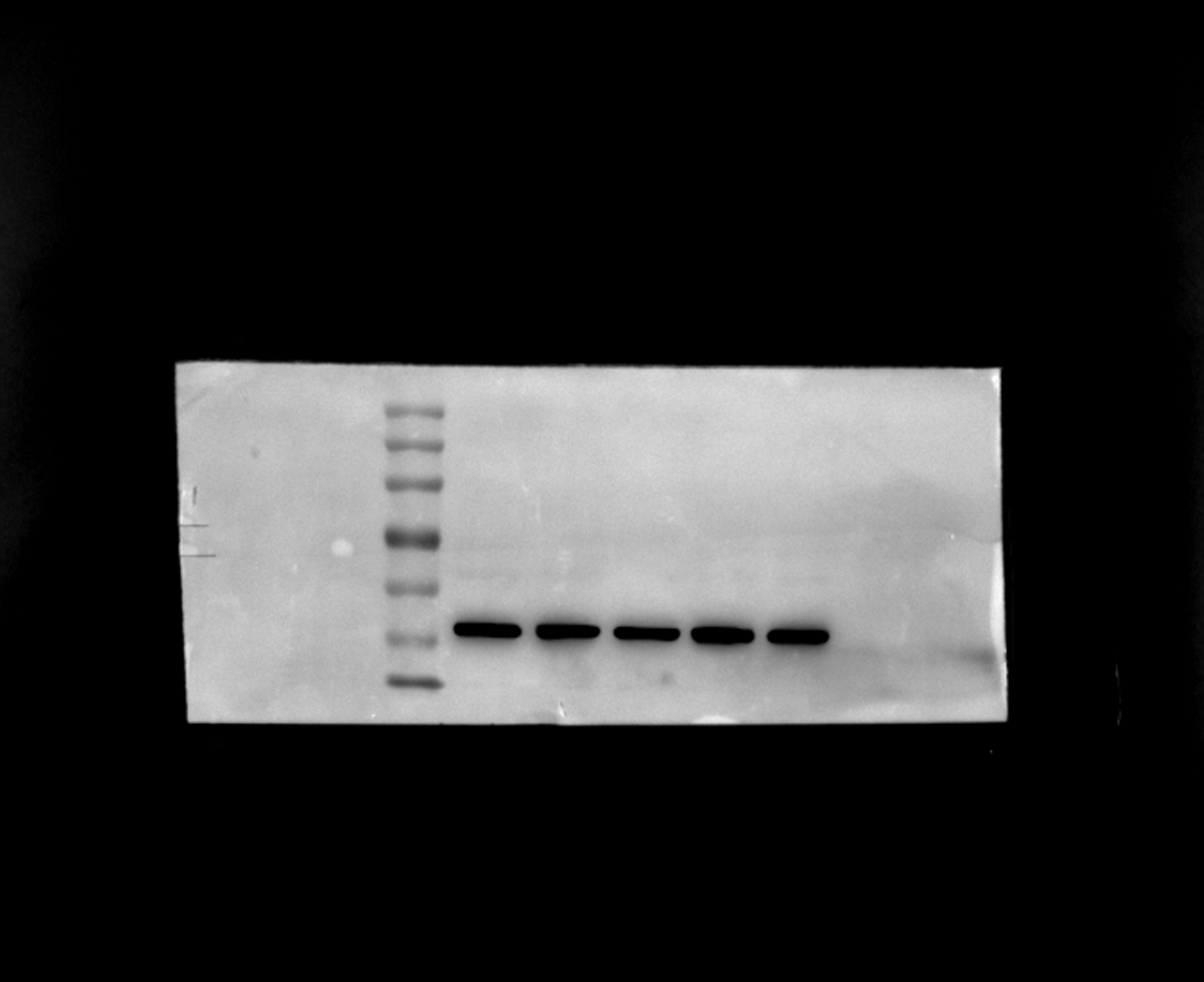

Supplement: Supplementary file 4 [file Data_Sheet_4.zip › uncropped-blot/2/NOS1 β-actin 2-2.Tif]

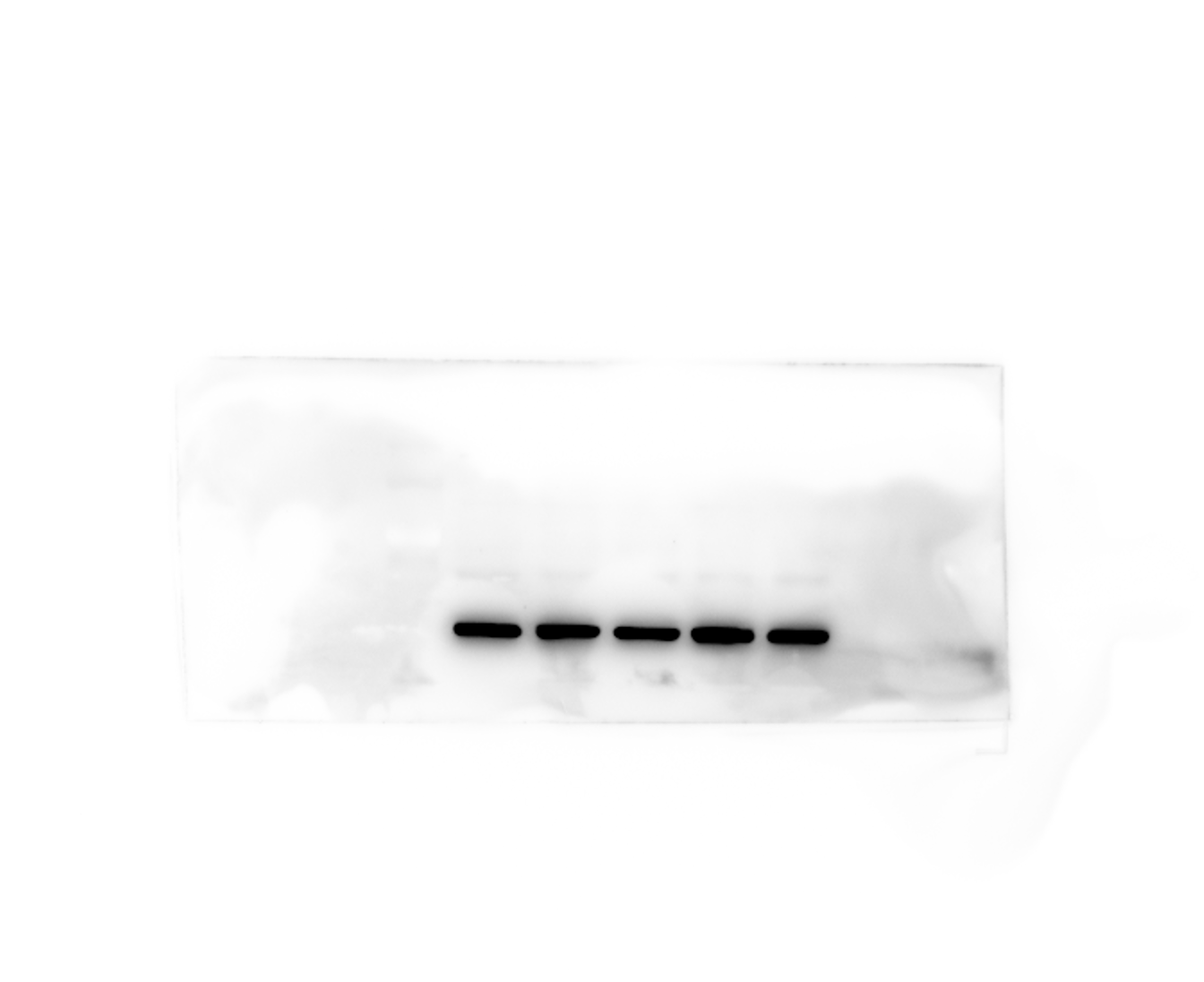

Supplement: Supplementary file 4 [file Data_Sheet_4.zip › uncropped-blot/2/NOS1 β-actin 2.Tif]

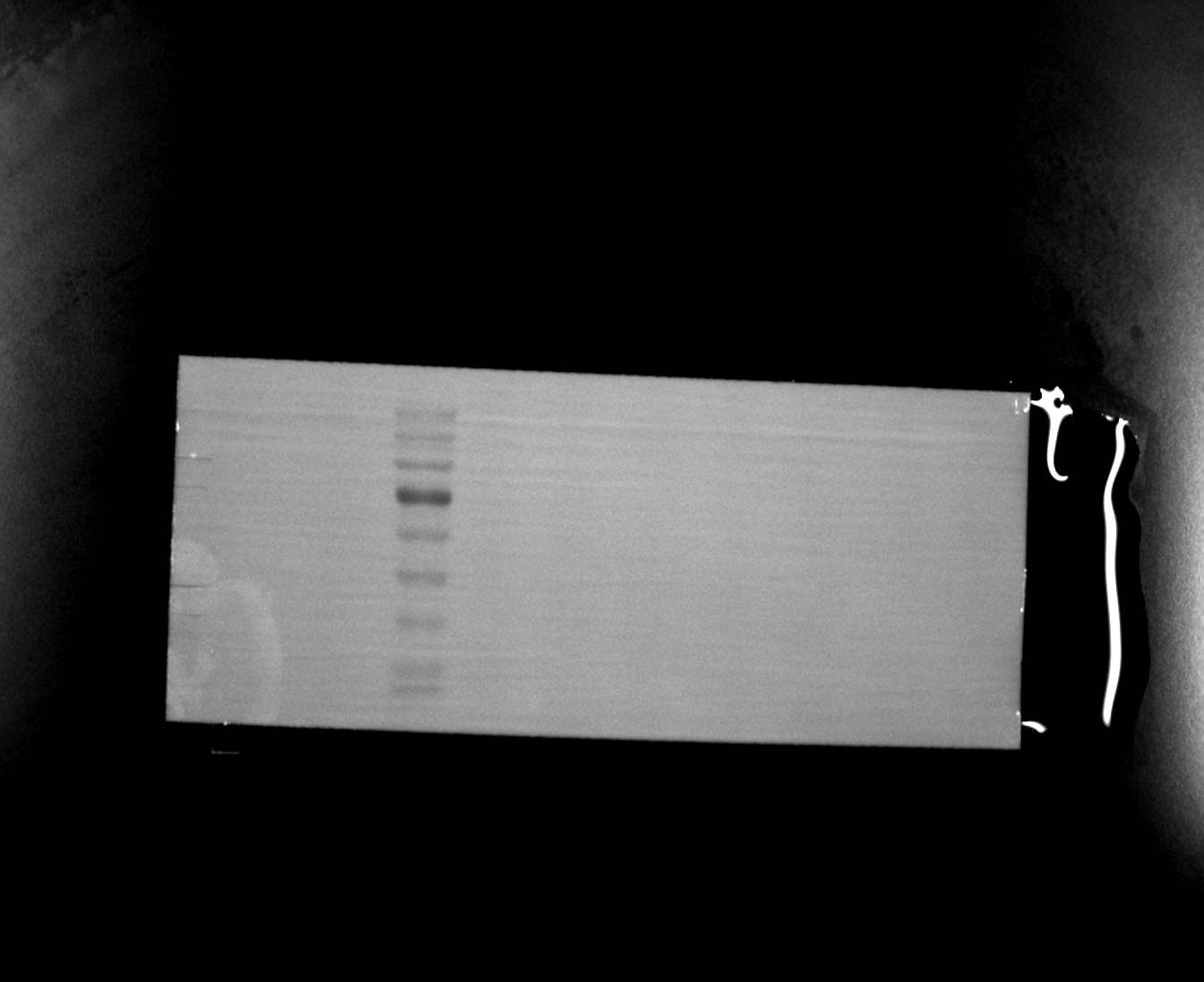

Supplement: Supplementary file 4 [file Data_Sheet_4.zip › uncropped-blot/3/NLRP3 3-1.Tif]

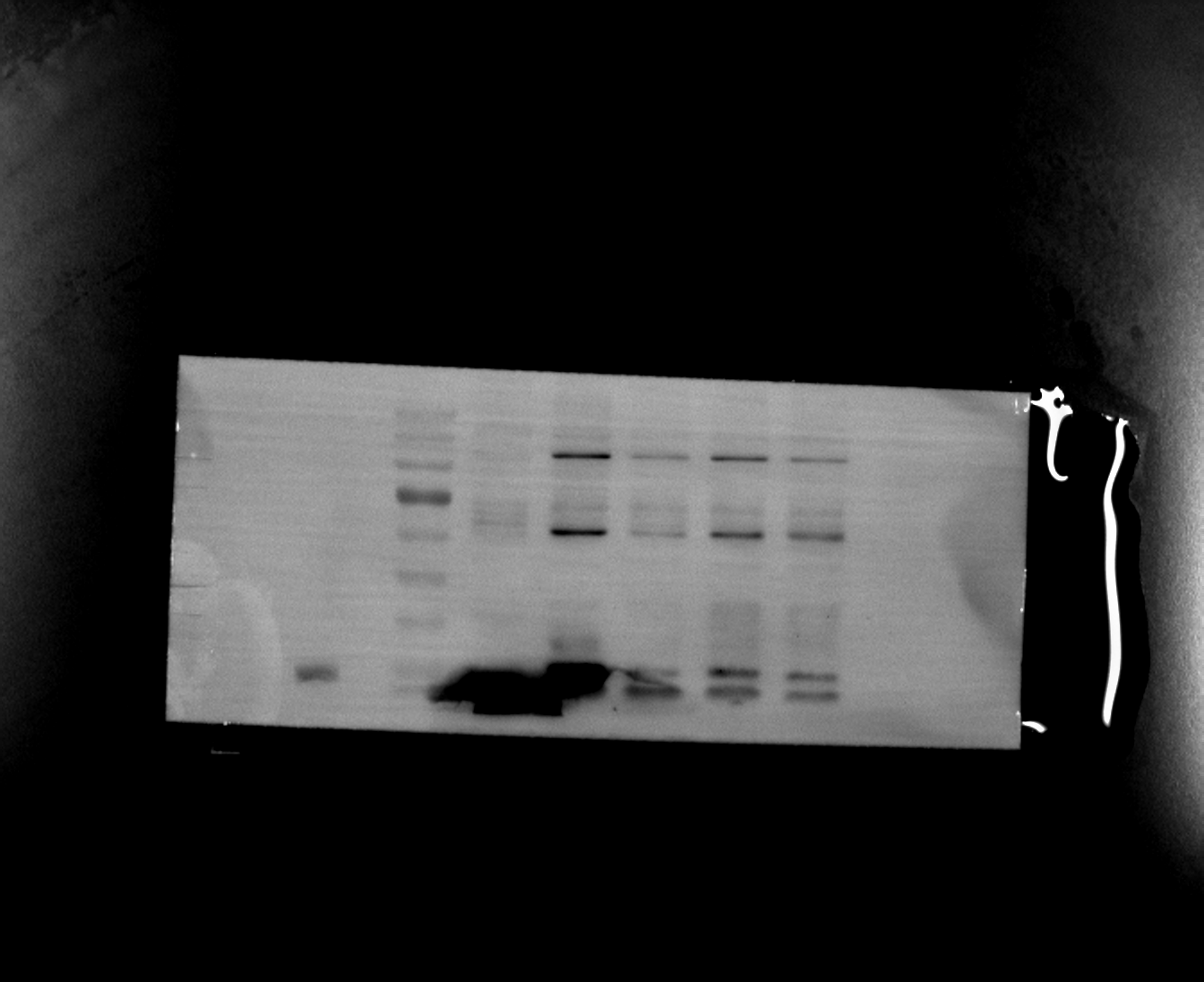

Supplement: Supplementary file 4 [file Data_Sheet_4.zip › uncropped-blot/3/NLRP3 3-2.tif]

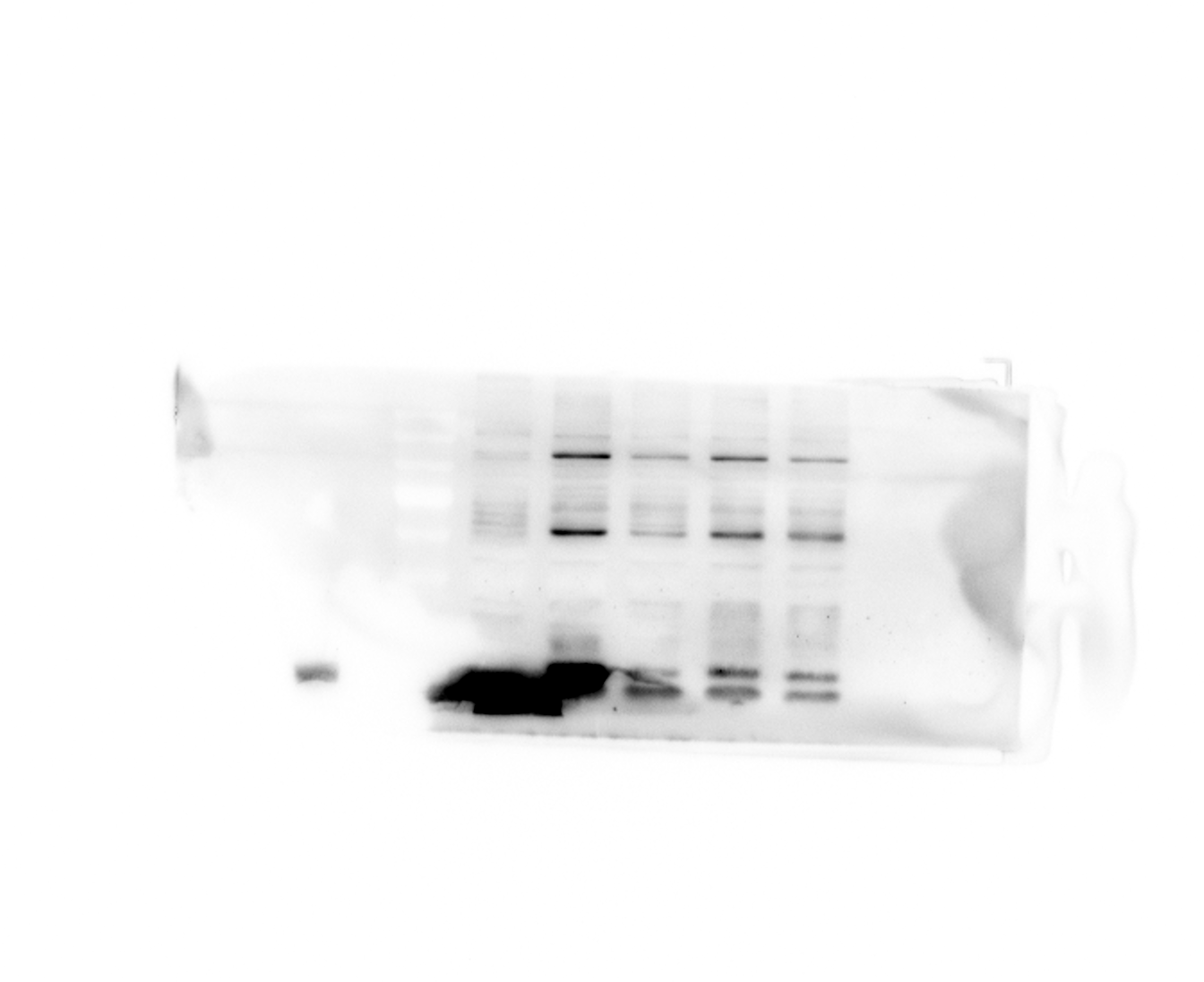

Supplement: Supplementary file 4 [file Data_Sheet_4.zip › uncropped-blot/3/NLRP3 3.Tif]

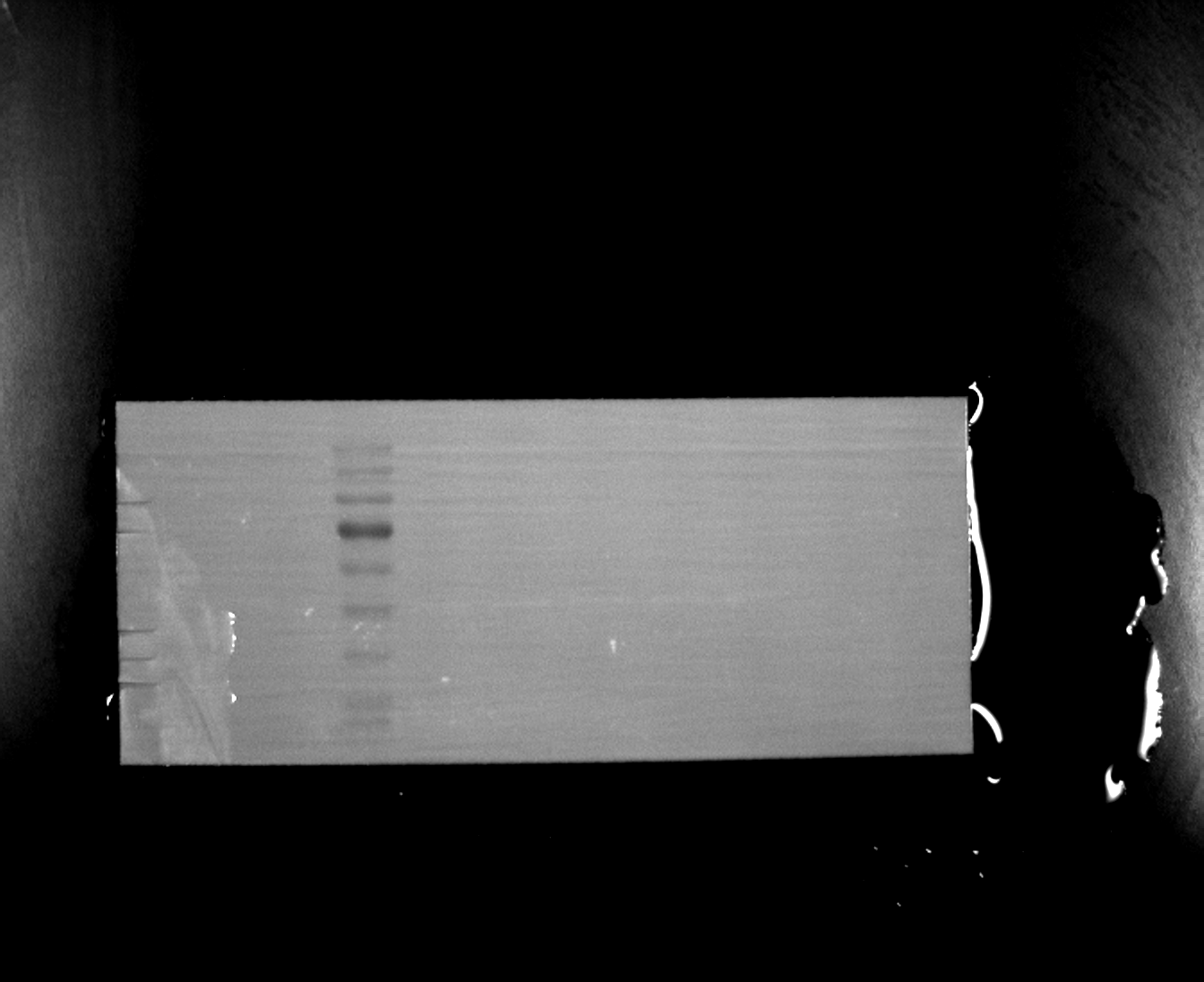

Supplement: Supplementary file 4 [file Data_Sheet_4.zip › uncropped-blot/3/NLRP3 β-actin 3-1.Tif]

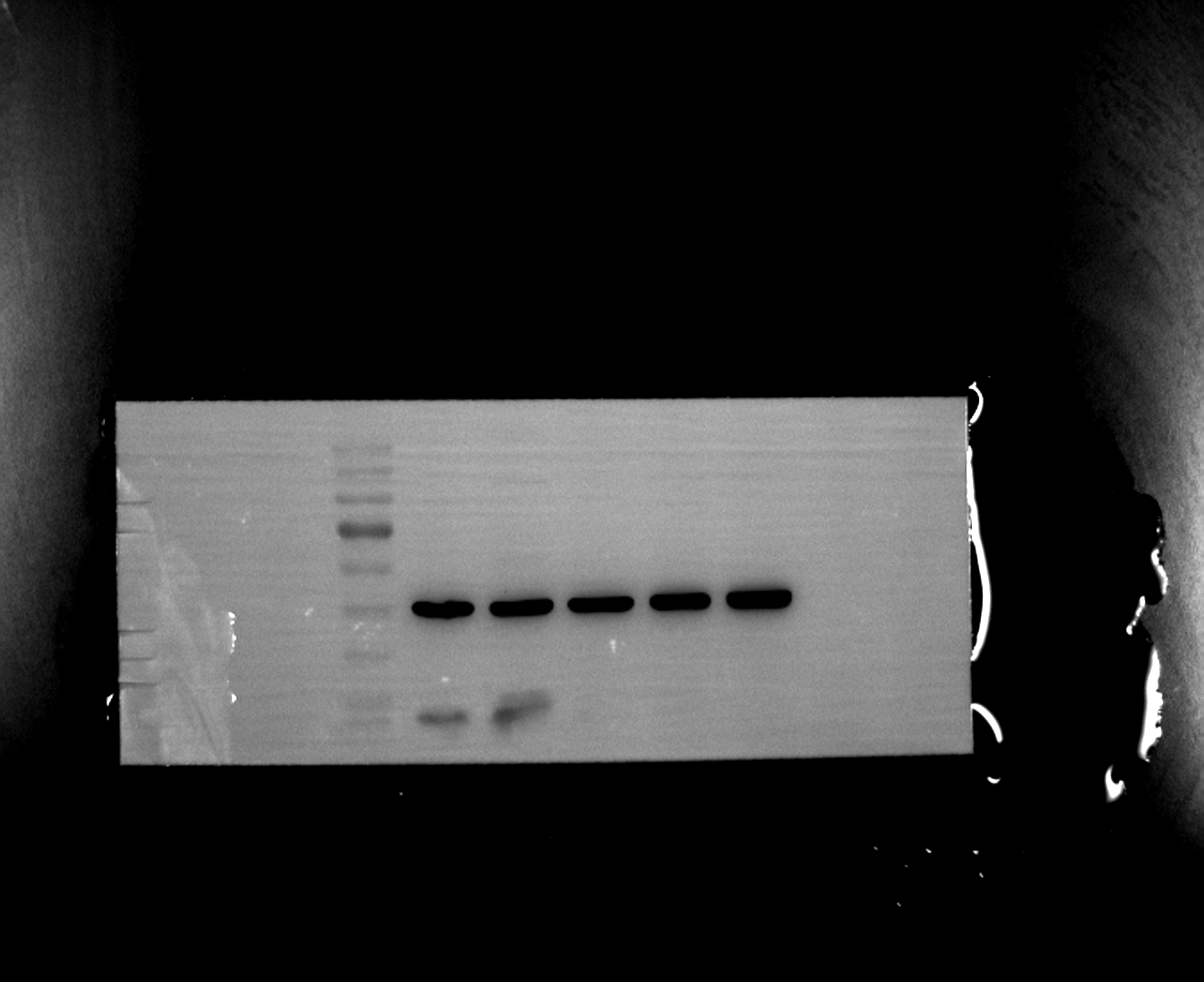

Supplement: Supplementary file 4 [file Data_Sheet_4.zip › uncropped-blot/3/NLRP3 β-actin 3-2.tif]

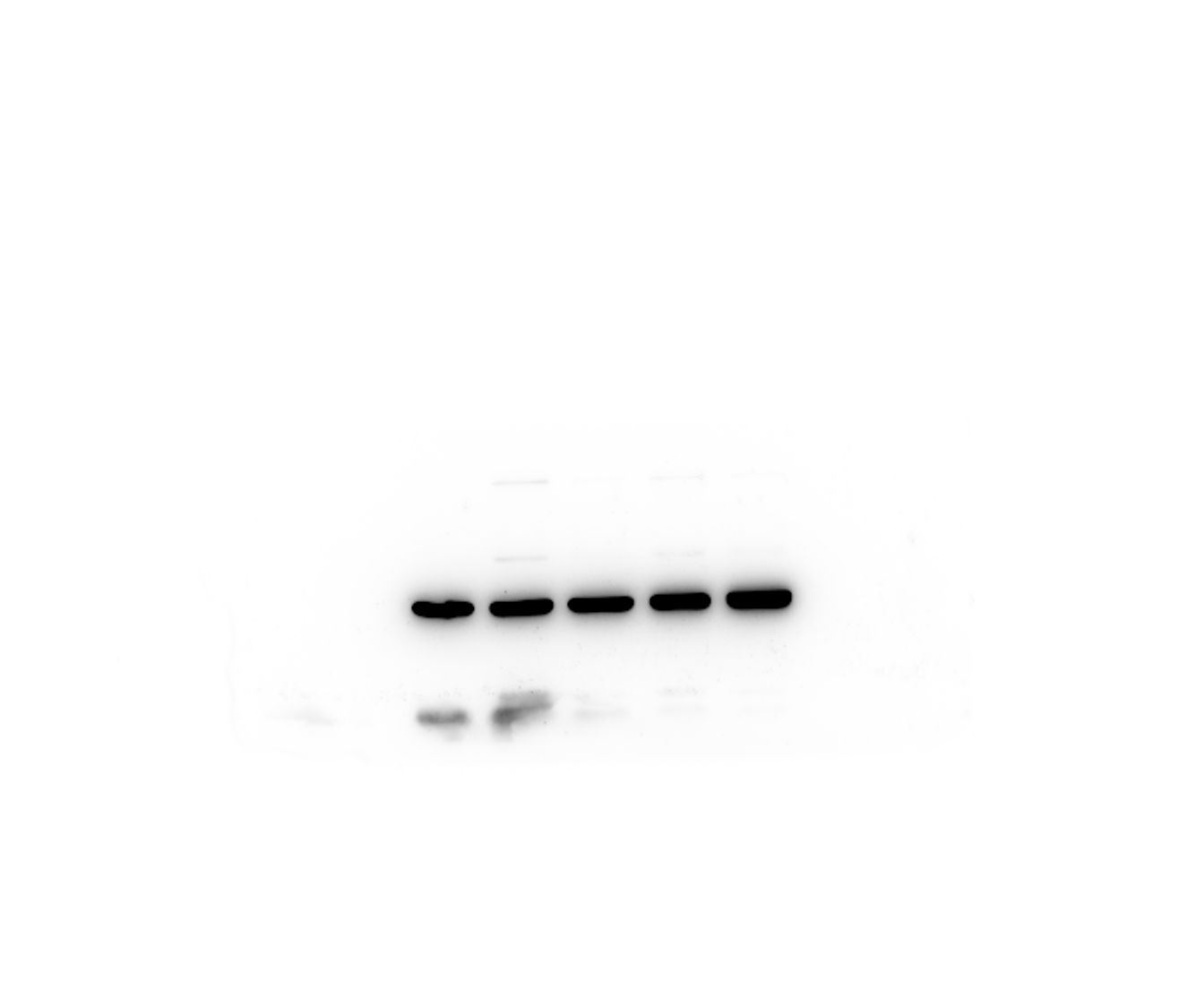

Supplement: Supplementary file 4 [file Data_Sheet_4.zip › uncropped-blot/3/NLRP3 β-actin 3.Tif]

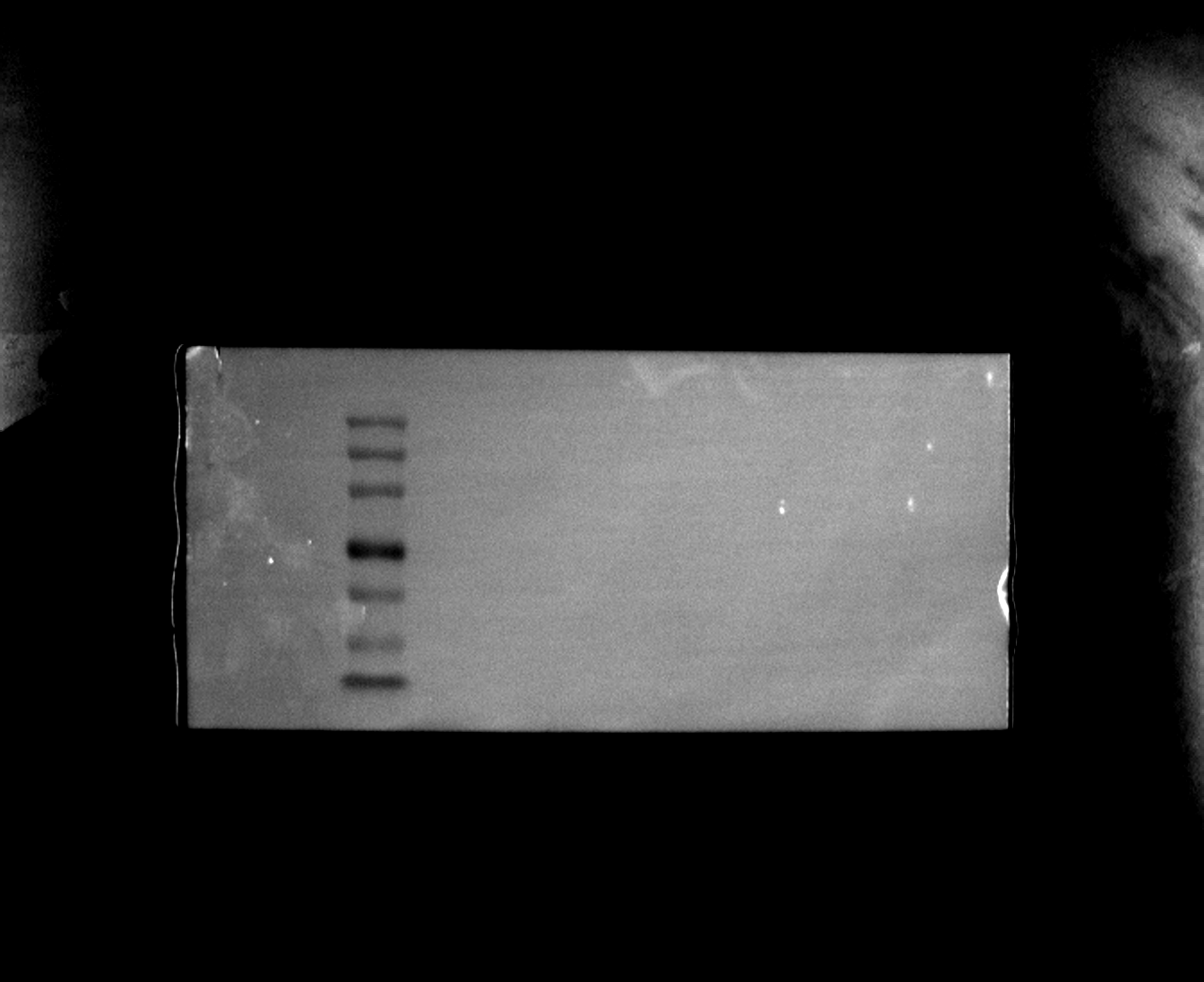

Supplement: Supplementary file 4 [file Data_Sheet_4.zip › uncropped-blot/3/NOS1 3-1.Tif]

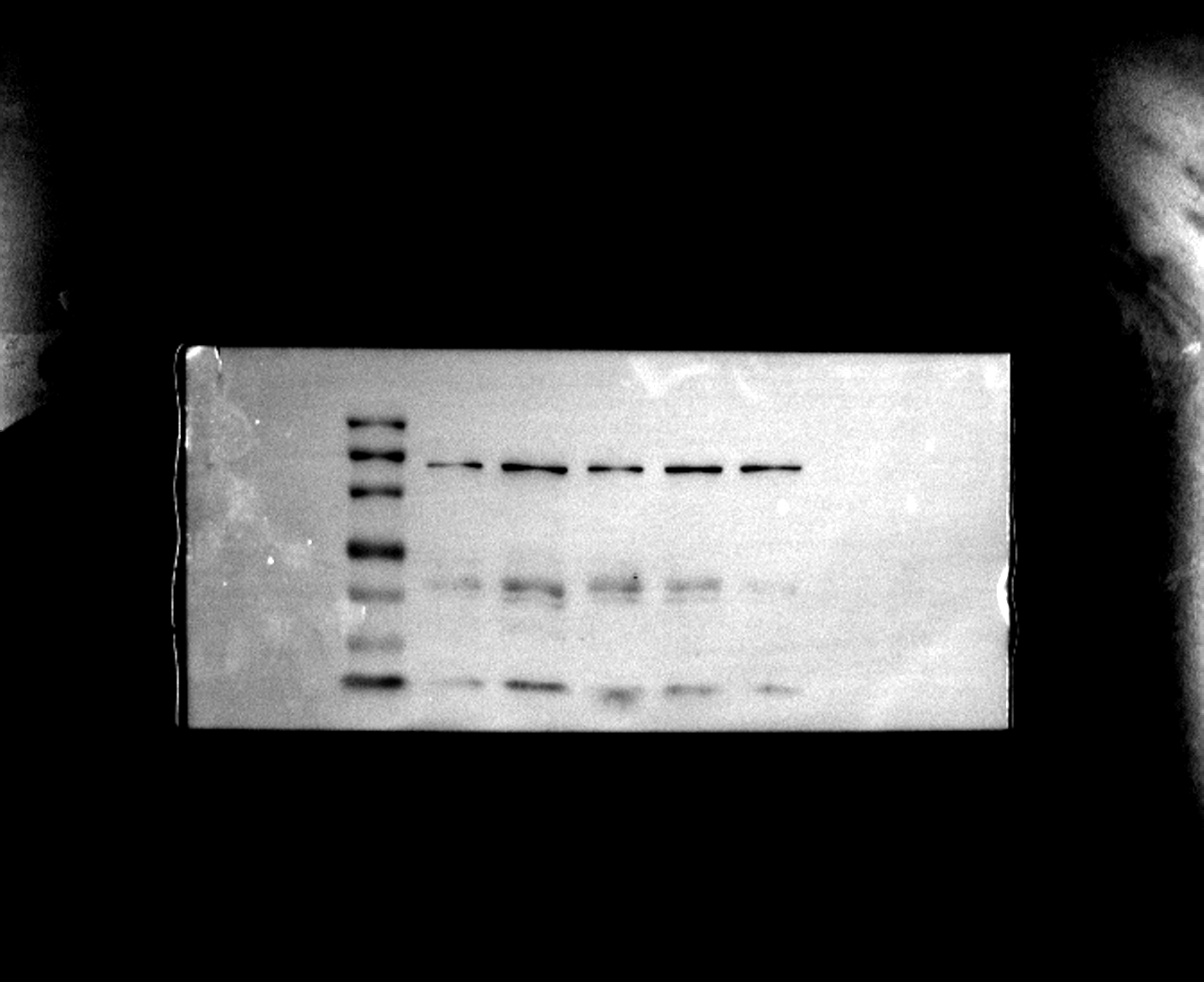

Supplement: Supplementary file 4 [file Data_Sheet_4.zip › uncropped-blot/3/NOS1 3-2.Tif]

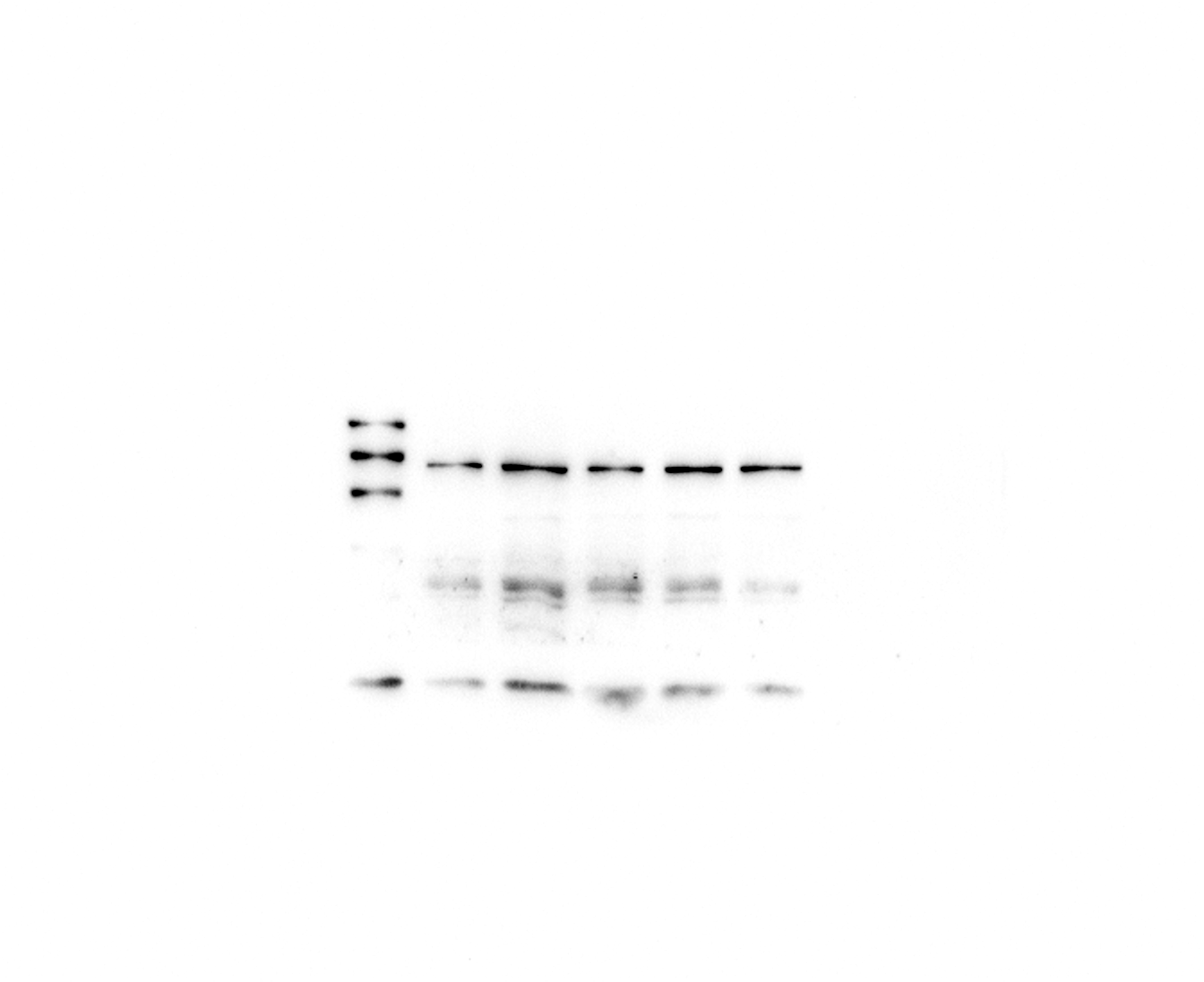

Supplement: Supplementary file 4 [file Data_Sheet_4.zip › uncropped-blot/3/NOS1 3.Tif]

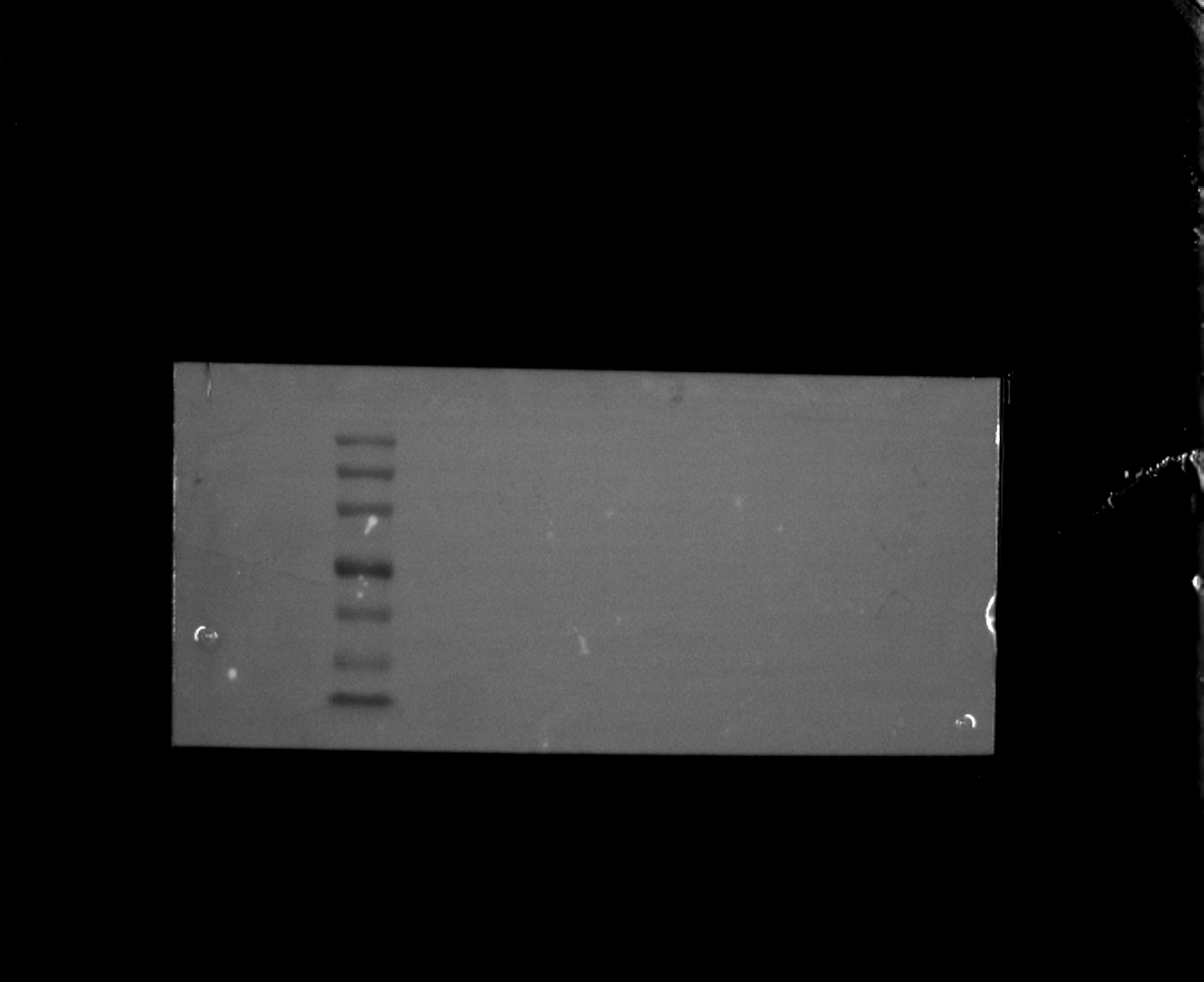

Supplement: Supplementary file 4 [file Data_Sheet_4.zip › uncropped-blot/3/NOS1 β-actin 3-1.Tif]

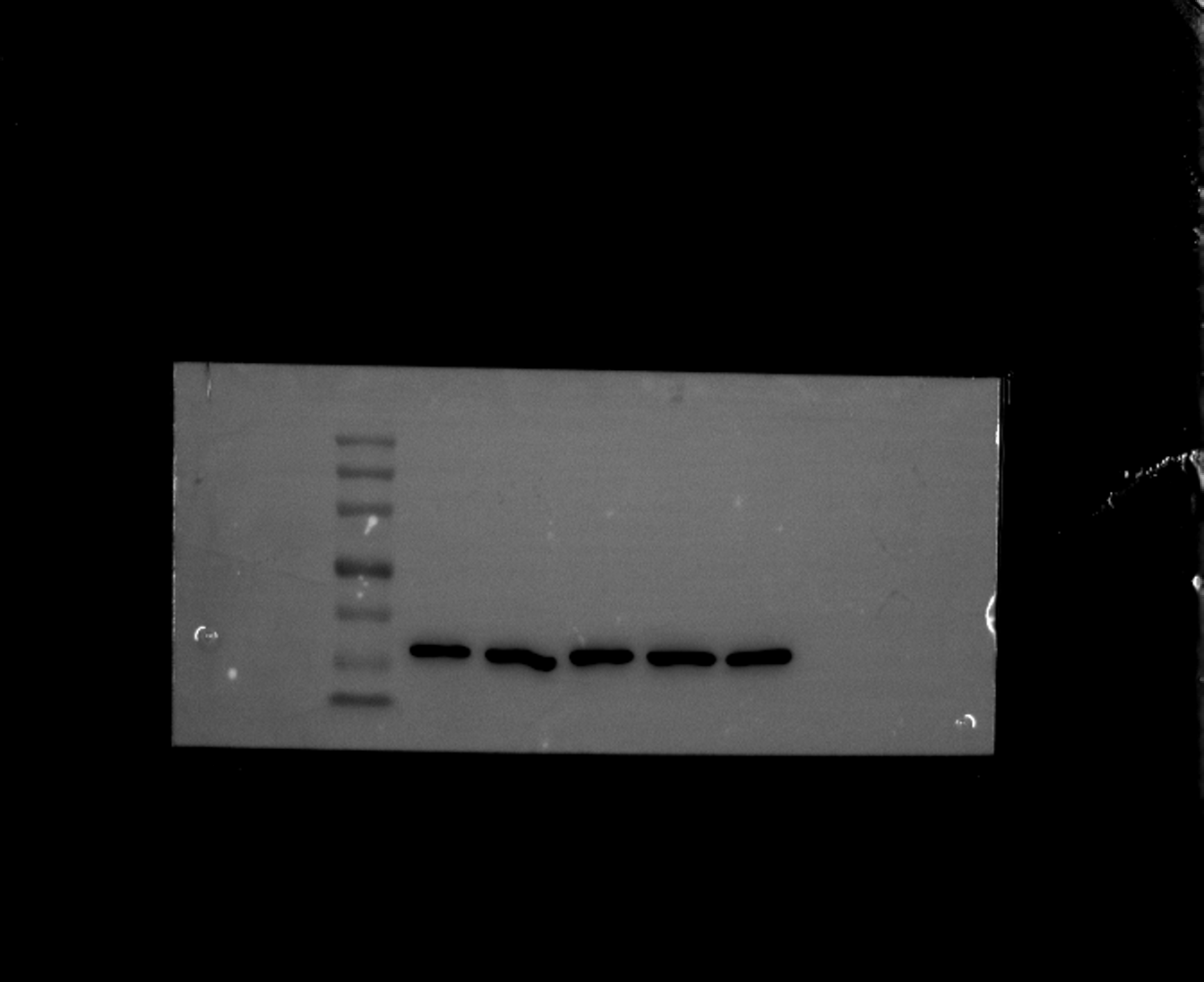

Supplement: Supplementary file 4 [file Data_Sheet_4.zip › uncropped-blot/3/NOS1 β-actin 3-2.Tif]

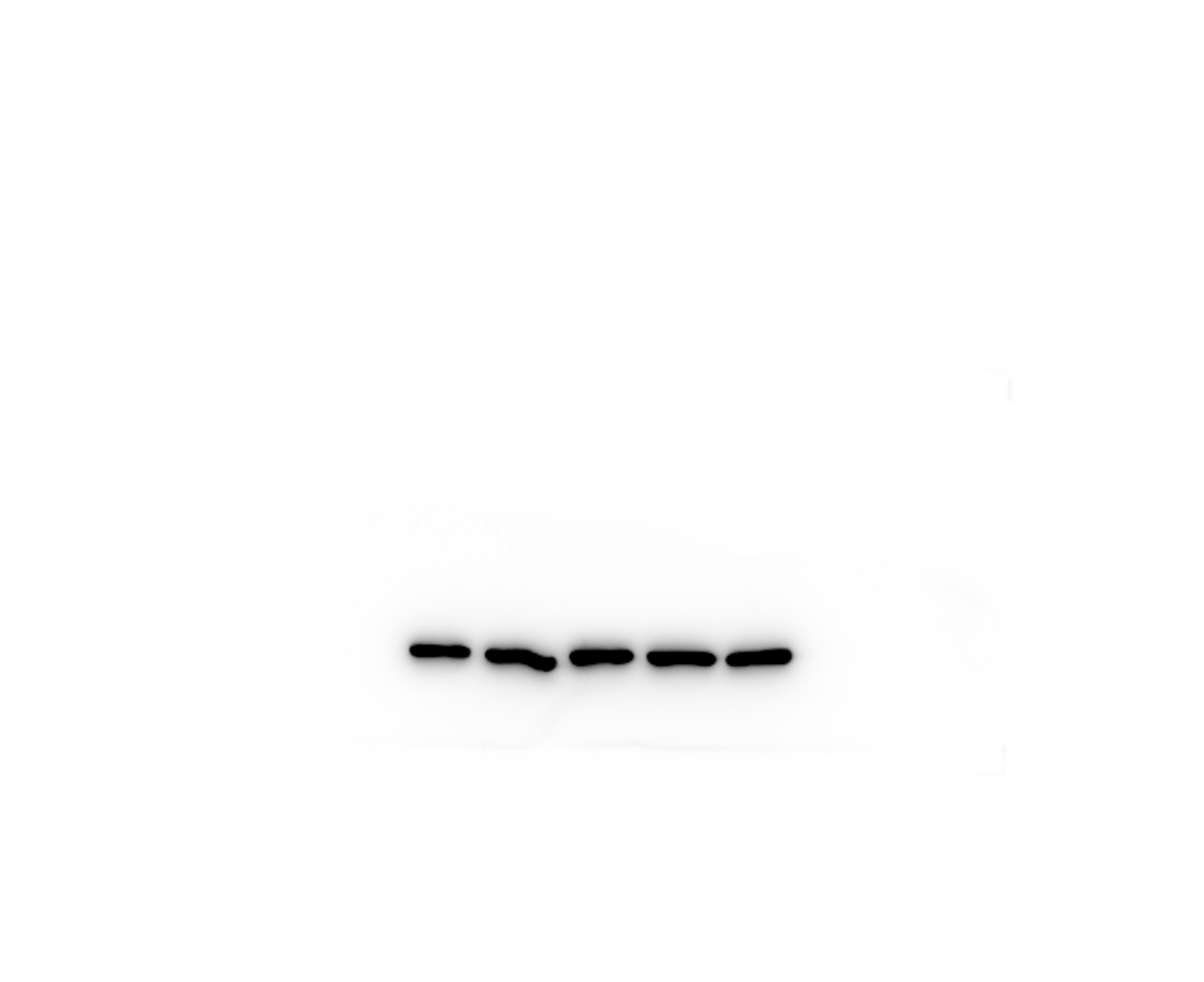

Supplement: Supplementary file 4 [file Data_Sheet_4.zip › uncropped-blot/3/NOS1 β-actin 3.Tif]
